# Chiral terpene auxiliaries V: Synthesis of new chiral γ-hydroxyphosphine oxides derived from α-pinene

**DOI:** 10.3762/bjoc.15.242

**Published:** 2019-10-22

**Authors:** Anna Kmieciak, Marek P Krzemiński

**Affiliations:** 1 Faculty of Chemistry, Nicolaus Copernicus University in Toruń, 7 Gagarin St., 87-100 Toruń, Poland

**Keywords:** isopinocamphone, monoterpenes, phosphines, [2,3]-sigmatropic rearrangement, verbanone

## Abstract

New chiral regioisomeric γ-hydroxyphosphine ligands were synthesized from α-pinene. The key transformation was the thermal [2,3]-sigmatropic rearrangement of allyldiphenylphosphinites, obtained from (1*R*,2*R*,4*S*,5*R*)-3-methyleneneoisoverbanol and (1*R*,2*R*,3*R*,5*R*)-4-methyleneneoisopinocampheol, to allylphosphine oxides. Hydroxy groups were introduced stereoselectively through a hydroboration–oxidation reaction proceeding from the less hindered site providing a *trans* relationship between the hydroxy and the phosphine substituents.

## Introduction

Chiral phosphorus compounds, despite many years of research, still enjoy unflagging interest of many research groups [1]. Compounds with a phosphorus atom attached to a stereogenic carbon center in acyclic and cyclic structures play an important role as chiral ligands in transition metal complexes [2]. They were applied to various catalytic asymmetric reactions [3–4], such as hydrogenations [3–6], conjugated additions to enones [7], and allylic alkylations [8–9]. Another direction of research is the use of phosphines in organocatalysis [10–11] and bifunctional catalysis [12].

Several methods were developed to introduce the phosphine functionality to organic molecules. The reaction of organometallics with chlorophosphines, the reaction of metal phosphides with haloalkanes, and transition-metal-catalyzed cross-coupling reactions to form C–P bonds are the most widely used methods for the synthesis of phosphines [13–14]. Since phosphines are easily oxidized to phosphine oxides, the addition of phosphine oxide P–H nucleophiles were also realized [15]. The phosphine oxide group can also be introduced starting from allylic alcohols employing the rearrangement of allylic diphenylphosphinites to allylphosphine oxides [16–17].

Recently, we have shown the synthesis and applications of chiral PHOX ligands that were obtained from readily available natural (1*S*)-β-pinene and (1*S*)-α-pinene [18]. We applied these ligands for the formation of the ruthenium complexes, which were successfully used as catalysts in asymmetric transfer hydrogenation of prochiral ketones.

In continuation of our studies on the synthesis of monoterpene derived ligands, we have utilized commercially available (1*R*)-α-pinene and (1*S*)-β-pinene to obtain regioisomeric exocyclic and endocyclic allylic alcohols, which were applied for the synthesis of γ-hydroxydiphenylphosphine ligands. To the best of our knowledge, only Knochel and co-workers synthesized diphosphines with a pinane framework [19].

## Results and Discussion

### Synthesis of allylic alcohols

**Scheme 1:**
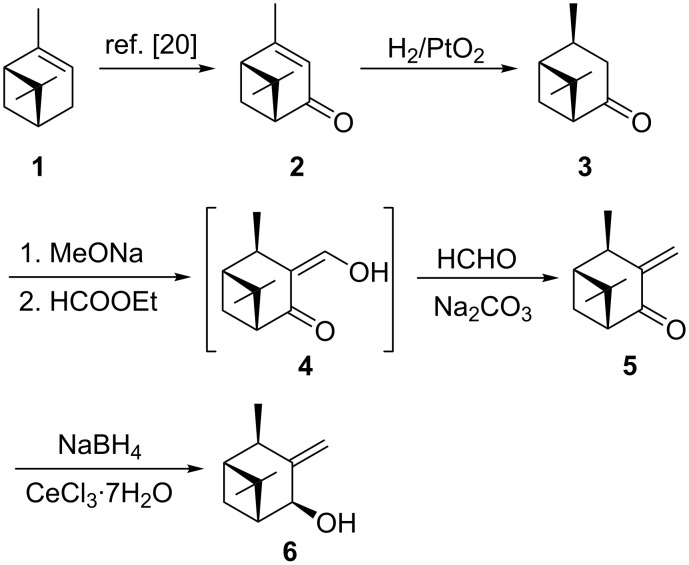
Synthesis of (1*R*,2*R*,4*S*,5*R*)-3-methyleneneoisoverbanol (**6**).

In the first step, commercially available natural (1*R*)-α-pinene (**1**) was oxidized with lead(IV) acetate to produce (+)-verbenone (**2**) in 58% yield (Scheme 1) [20]. Hydrogenation of **2** with Adams catalyst was carried out in cyclohexane with 1 atm of hydrogen. The hydrogen pressure was increased to 10 atm for the reaction performed in an autoclave on a larger scale maintaining the high selectivity of hydrogen addition from the less hindered side of the molecule. (1*R*,2*S*,5*R*)-(+)-Verbanone (**3**) was obtained in 93% yield. GC analysis of **3** showed the presence of *cis* and *trans* diastereoisomers in a ratio of 97:3. The hydrogen-addition selectivity is consistent with earlier literature reports and results from the shielding effect of the *gem*-dimethyl bridge [21–22].

In the next step, **3** reacted with sodium methoxide in toluene and the resulting enolate was condensed with ethyl formate to give a keto-aldehyde, which tautomerized into the more stable β-hydroxyenone **4** [23]. The intermediate **4** was sufficiently pure for the subsequent transaldolization reaction with formaldehyde in the presence of sodium carbonate to give (+)-3-methyleneverbanone (**5**) in 57% yield from **3**. α,β-Unsaturated ketone **5** was exclusively reduced to allylic alcohol (1*R*,2*R*,4*S*,5*R*)-3-methyleneneoisoverbanol (**6**), using the Luche method [24]. 1,2-Reduction of enone **5** was achieved with sodium borohydride in the presence of cerium(III) chloride in methanol in 88% yield (Scheme 1).

**Scheme 2:**
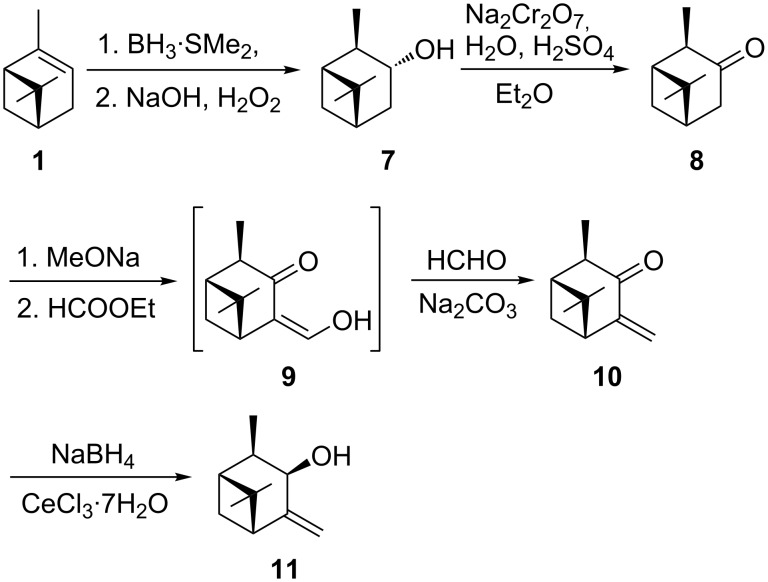
Synthesis of (1*R*,2*R*,3*R*,5*R*)-4-methyleneneoisopinocampheol (**11**).

The synthesis of allylic alcohol **11**, a regioisomer of **6**, started again from (1*R*)-α-pinene (**1**, Scheme 2). Hydroboration of (1*R*)-α-pinene with borane–dimethyl sulfide adduct (BMS) and crystallization of the product diisopinocampheylborane (*^d^*Ipc_2_BH, 84% yield) allowed to upgrade the enantiomeric purity of *^d^*Ipc_2_BH [25]. Oxidation of the resulting dialkylborane with hydrogen peroxide provided enantiomerically pure (−)-isopinocampheol (**7**) in 78% yield. The Brown–Garg protocol [26] was employed to oxidize **7** with an aqueous solution of sodium dichromate and sulfuric acid under biphasic conditions. (−)-Isopinocamphone (**8**) was purified by fractional distillation and isolated in 78% yield. Then, **8** was subjected to an analogous reaction sequence that was used for (+)-verbanone (**3**), i.e., the synthesis of the enone in the first step followed by its 1,2-reduction to the allylic alcohol. Thus, Claisen condensation of **8** with ethyl formate gave β-hydroxyenone **9**, which was subjected to transaldolization with formaldehyde producing the corresponding 4-methyleneisopinocamphone (**10**) in 83% yield. Luche reduction of the latter compound provided (1*R*,2*R*,3*R*,5*R*)-4-methyleneneoisopinocampheol (**11**) in 84% yield.

**Scheme 3:**
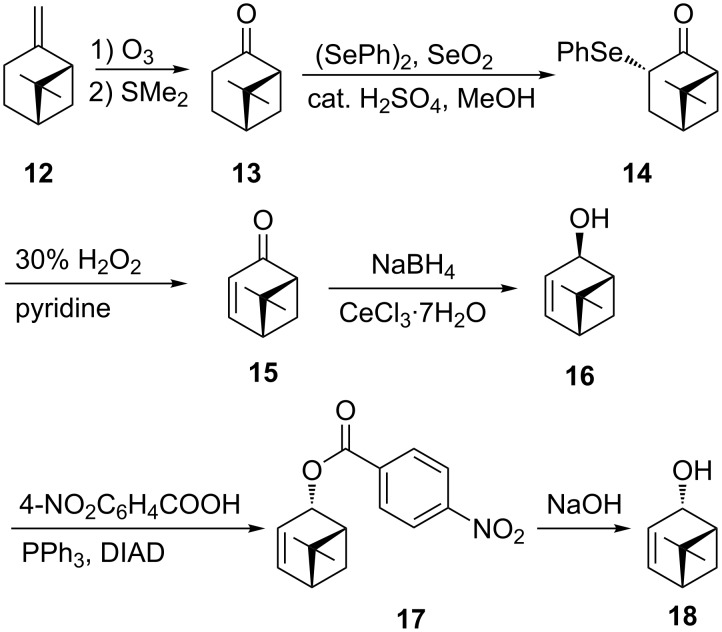
Synthesis of allylic alcohols **16** and **18** from β-pinene.

In order to obtain two endocyclic allylic alcohols, (−)-β-pinene was chosen as the starting material for this synthesis (Scheme 3). Thus, β-pinene (**12**) was reacted with ozone to give (+)-nopinone (**13**) in 90% yield [27]. In the next step, **13** reacted with diphenyl diselenide and selenium dioxide in methanol [28]. The obtained phenyl selenide **14** was oxidized with hydrogen peroxide in the presence of pyridine to selenoxide, which readily undergoes intramolecular *syn*-elimination to produce α,β-unsaturated (+)-apoverbenone (**15**) [28–29]. In the next step, Luche reduction of **15**, proceeding from the less hindered side of the carbonyl group, gave (1*R*,4*R*,5*R*)-apopinenol (**16**) in 95% yield. GC analysis of **16** has shown the presence of the expected major isomer (1*R*,4*R*,5*R*)-**16** (92%) and the minor isomer (1*R*,4*S*,5*R*)-**18** (8%). Diastereomers **16** and **18** can be separated by flash column chromatography on silica gel. For the purpose of this study, (1*R*,4*R*,5*R*)-apopinenol (**16**) was subjected to the Mitsunobu reaction to obtain the product with inverted configuration at C4. Alcohol **16** was reacted with diisopropyl azodicarboxylate, triphenylphosphine, and *p*-nitrobenzoic acid in THF [30]. ^1^H NMR analysis of the crude *p*-nitrobenzoate **17** revealed a mixture of the predicted *p*-nitrobenzoate of apopinenol **18** together with an ester of **16** in a ratio of 79:21. Attempts to separate *p*-nitrobenzoates of **18** and **16** by column chromatography on silica gel failed. The mixture of esters, after purification, was hydrolyzed with a 5% aqueous solution of NaOH. GC analysis of the isolated alcohol confirmed the presence of (1*R*,4*S*,5*R*)-apopinenol (**18**) and (1*R*,4*R*,5*R*)-**16** in a ratio of 79.5:20.5.

**Figure 1:**
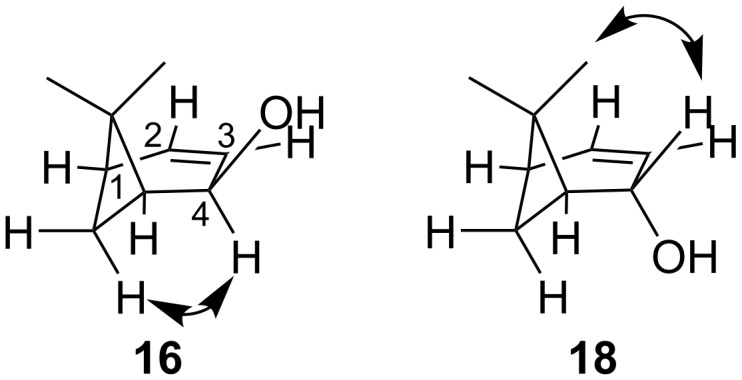
NOE effects in molecules **16** and **18**.

The structures and stereochemistry of both diastereomeric alcohols **16** and **18** were confirmed by 2D NMR spectra. All protons in **16** and **18** were assigned using ^1^H,^1^H-COSY spectra. The configurations at C4 were established by correlations observed in their ^1^H,^1^H-NOESY spectra (Figure 1, Supporting Information File 1).

### Synthesis of γ-hydroxyphosphines

**Scheme 4:**
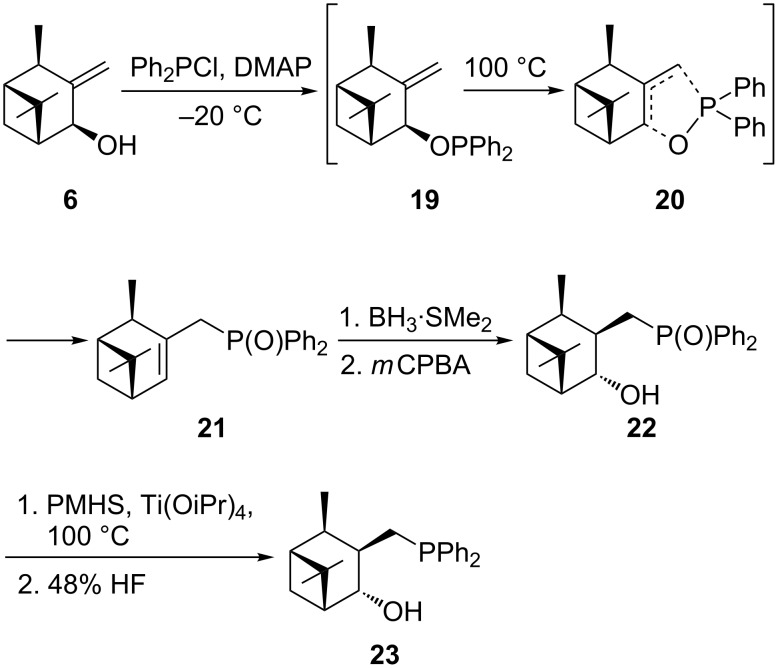
Synthesis of (1*R*,2*R*,3*R*,4*R*,5*R*)-3-((diphenylphosphanyl)methyl)isoverbanol (**23**).

The key step in the synthesis to introduce a phosphine functionality is the thermal [2,3]-sigmatropic rearrangement of an allylic diphenylphosphinite to the diphenylphosphine oxide (Scheme 4) [16].

Diphenylphosphinite **19** was formed in the reaction of allylic alcohol **6** with diphenylphosphine chloride in the presence of DMAP at −20 ºC. The temperature was raised to induce phosphinite’s [2,3]-sigmatropic rearrangement (**20**) as shown in Scheme 4. The reaction progress, conversion of **19** into **21**, was monitored by ^31^P NMR (ROPPh_2_ δ = 113 ppm; RP(O)Ph_2_ δ = 30 ppm). The phosphinite **19** disappeared after 48 h at 100 °C. The product was crystallized from heptane to give phosphine oxide **21** in 92% yield. The allylic diphenylphosphine oxide **21** was subjected to the hydroboration–oxidation reaction introducing stereoselectively the hydroxy group. Hydroboration was carried out with an excess of borane–dimethyl sulfide adduct followed by the oxidation step. The standard C–B bond oxidation protocol (H_2_O_2_/NaOH) proceeded with the low yield (32%). Application of *m*-chloroperbenzoic acid (*m*CPBA) as an oxidant, similarly to Knochels findings [19], gave the higher yield (56%) of (((1*R*,2*R*,3*R*,4*R*,5*R*)-4-hydroxypinan-3-yl)methyl)diphenylphosphine oxide (**22**).

Next, phosphine oxide **22** was reduced to the phosphine with poly(methylhydrosiloxane) in the presence of titanium(IV) isopropoxide (Scheme 4). The work-up of the reaction mixture with 25% HF allowed to remove silicon and titanium impurities and purification of **23** by column chromatography on silica gel yielded the product in 62%.

**Scheme 5:**
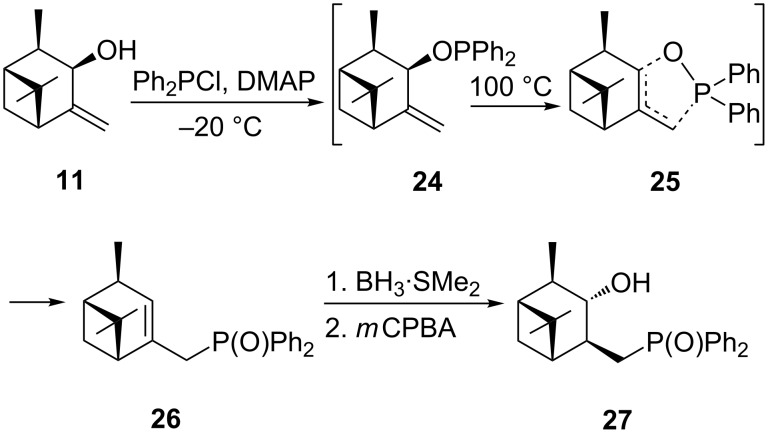
Synthesis of (((1*R*,2*R*,3*S*,4*S*,5*S*)-3-hydroxypinan-4-yl)methyl)diphenylphosphine oxide (**27**).

4-Methyleneneoisopinocampheol (**11**) was subjected to the same reaction sequence as **6** (Scheme 5). The allylphosphine oxide **26** was obtained after [2,3]-sigmatropic rearrangement of phosphinite **24**. After purification by column chromatography on silica gel, **26** was obtained in 78% yield. The hydroboration of **26** was carried out with borane–dimethyl sulfide adduct in THF at 50 ºC. Oxidation of the alkylborane with *m*CPBA gave the desired alcohol **27** in 51% overall yield.

**Scheme 6:**
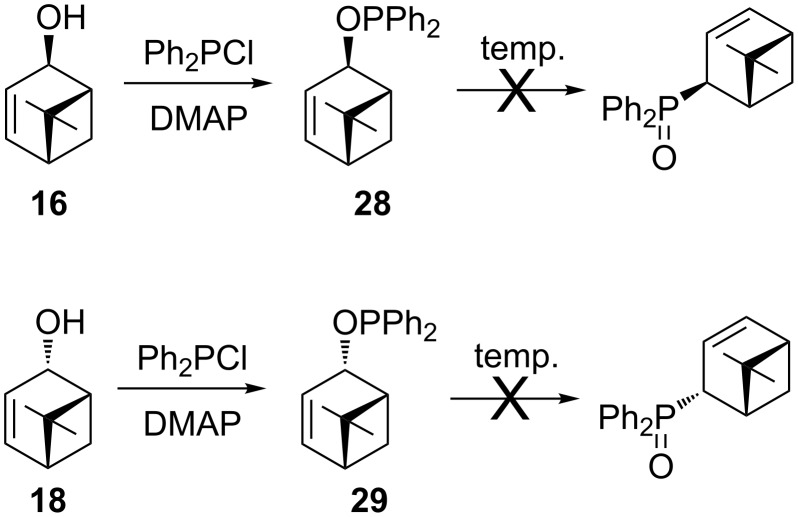
Attempted sigmatropic rearrangement of phosphinites **28** and **29**.

Finally, diastereomeric endocyclic allylic alcohols **16** and **18** were treated with chlorodiphenylphosphine in the presence of DMAP to produce diphenylphosphinites **28** and **29** (^31^P NMR: ROPPh_2_ δ = 107 ppm), respectively (Scheme 6). Attempts to carry out a sigmatropic rearrangement in toluene at 100 ºC as well as in xylene at 140 ºC failed. The formation of the phosphine oxide products was not observed by ^31^P NMR analysis. The probable reason for the lack of the rearrangement reaction may be the rigid bicyclic structure of the substrate and steric hindrance on one side of the molecule caused by the *gem*-dimethyl bridge.

## Conclusion

Regioisomeric exocyclic allylic alcohols were synthesized from natural α-pinene. (1*R*,2*R*,4*S*,5*R*)-3-Methyleneneoisoverbanol and (1*R*,2*R*,3*R*,5*R*)-4-methyleneneoisopinocampheol were synthesized using known procedures. They were used in the key transformation, the thermal [2,3]-sigmatropic rearrangement of allyldiphenylphosphinites to allyldiphenylphosphine oxides. Allylphosphine oxides were further functionalized through a hydroboration–oxidation reaction occurring from the less hindered side of the molecule to produce γ-hydroxyphosphine oxide derivatives. It was also shown that phosphine oxide can be reduced to phosphine without affecting the bicyclic pinane structure.

The synthesis of diastereomeric endocyclic allylic alcohols from (−)-β-pinene was also carried out. Unfortunately, due to the probably too rigid bicyclic structure of the diphenylphosphinites, they did not undergo the sigmatropic rearrangement to the corresponding phosphine oxides. Further research and application of the obtained ligands are in progress.

## Experimental

### Diphenyl(((1*R*,2*R*,5*S*)-δ-pinen-3-yl)methyl)phosphine oxide (**21**)

Allylic alcohol **6** (0.831 g, 5 mmol) and 4-dimethylaminopyridine (0.645 g, 5.3 mmol) were placed in a Schlenk flask. These compounds were dissolved in toluene (10 mL) under nitrogen. The solution was cooled below –20 °C and diphenylphosphine chloride (1.106 g, 5 mmol) was added dropwise. After the removal of the cooling bath, the solution was stirred for 24 h at 100 °C. After this time, the ^31^P NMR spectrum has shown incomplete conversion of the substrate. Consequently, heating was continued until the ^31^P NMR spectrum showed complete substrate conversion (48 h). Warm toluene (10 mL) was added to the reaction mixture and the solution was filtered through a pad of celite. The precipitate was washed with another portion of warm toluene (10 mL). The solvent was removed using a rotary evaporator to give the phosphine oxide. After crystallization from heptane, phosphine oxide **21** (1.612 g, 92%) was obtained as a white solid, mp 127–131 °C, 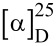 −98 (*c* 2.4, CHCl_3_); ^1^H NMR (700 MHz, CDCl_3_) δ 0.87 (d, *J* = 8.5 Hz, 1H), 0.89 (s, 3H, CH_3_), 1.14 (d, *J* = 7.5 Hz, 3H, CH_3_), 1.20 (s, 3H, CH_3_), 1.95–1.96 (m, 2H), 2.23 (dt, *J* = 8.4, 5.6 Hz, 1H), 2.56 (s, 1H), 3.08–3.12 (m, 1H), 3.20–3.25 (m, 1H), 5.97 (s, 1H), 7.45–7.48 (m, 4H), 7.49–7.53 (m, 2H), 7.76–7.80 (m, 4H); ^13^C NMR (100 MHz, CDCl_3_) δ 16.78 (d, *J* = 1.6 Hz), 23.73, 27.11, 33.52, 34.29 (d, *J* = 4.0 Hz), 38.22 (d, *J* = 4 Hz), 42.30 (d, *J* = 5.6 Hz), 42.31, 48.33, 128.33 (d, *J* = 11.9 Hz), 128.51 (d, *J* = 11.9 Hz), 129.09, 129.19, 130.84 (d, *J* = 9.5 Hz), 131.23 (d, *J* = 8.7 Hz), 131.60 (d, *J* = 8.7 Hz), 131.64 (d, *J* = 9.0 Hz), 135.87 (d, *J* = 8.0 Hz), 135.90 (d, *J* = 9.5 Hz); ^31^P NMR (162 MHz, CDCl_3_) δ 31.76; anal. calcd for C_23_H_27_OP: C, 78.83; H, 7.77; found: C, 78.73; H, 7.61.

### (((1*R*,2*R*,3*R*,4*R*,5*R*)-4-Hydroxypinan-3-yl)methyl)diphenylphosphine oxide (**22**)

Phosphine oxide **21** (1.402 g, 4 mmol) was dissolved in THF (13 mL) under nitrogen. BMS (10 M, 0.8 mL, 8 mmol) was added dropwise to the reaction mixture. The mixture was stirred for 24 h at 50 °C. After this time, the solution was cooled to room temperature and methanol (6 mL) was carefully added until the gas evolution ceased. Solvents were removed using a rotary evaporator and the resulting intermediate was dissolved in dichloromethane (6 mL). *meta*-Chloroperbenzoic acid (75%, 2.301 g, 10 mmol) was dissolved in dichloromethane (10 mL), cooled in a dry ice–acetone bath, and the intermediate solution was added dropwise. After 3 h, the mixture was filtered, 10% sodium metabisulphite (15 mL) was added to the filtrate, and the mixture was stirred for 10 min. The layers were separated, the organic layer was washed with 1 M NaOH (2 × 15 mL), brine (10 mL), and dried over anhydrous magnesium sulfate. The solution was filtered, the solvent evaporated on a rotary evaporator and the product was purified by flash chromatography on silica gel (dichloromethane/diethyl ether 10:90). Phosphine oxide **22** (0.825 g, 56%) was obtained as an oil.

In a second oxidation procedure, methanol (5 mL) and a 3 M solution of sodium hydroxide (2.7 mL, 8.1 mmol) were carefully added. After cooling to 0 °C, a 30% solution of hydrogen peroxide (1.2 mL, 12 mmol) was added dropwise to the reaction mixture. The solution was stirred for 30 minutes at rt and 1 h at 50 °C. After this time, potassium carbonate was added to saturate the solution. The layers were separated and the aqueous layer was extracted with diethyl ether (2 × 20 mL). The combined organic layers were washed with brine (15 mL), dried with anhydrous magnesium sulfate, filtered and the solvents were removed using a rotary evaporator. The product was purified by column chromatography on silica gel (dichloromethane/diethyl ether 10:90). **22** (0.472 g, 32%) was isolated as a white solid (mp 179–183 °C, 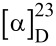
−39 (*c* 3.0, CHCl_3_); ^1^H NMR (400 MHz, CDCl_3_) δ 0.91 (s, 3H, CH_3_), 1.05 (d, *J* = 7.3 Hz, 3H, CH_3_), 1.22 (s, 3H, CH_3_), 1.38 (d, *J* = 9.3 Hz, 1H), 1.87–1.93 (m, 1H), 2.05–2.13 (m, 2H), 2.30–2.46 (m, 3H), 2.54–2.61 (m, 1H), 4.02 (d, *J* = 4.2 Hz, 1H), 7.46–7.58 (m, 6H), 7.74–7.82 (m, 4H); ^13^C NMR (100 MHz, CDCl_3_) δ 16.84, 23.18, 25.39, 27.79, 32.13 (d, *J* = 69.1 Hz), 36.32 (d, *J* = 12.7 Hz), 37.52 (d, *J* = 4.0 Hz), 39.48, 47.36, 48.80, 75.87, 128.74 (d, *J* = 9.5 Hz), 128.86 (d, *J* = 10.3 Hz), 130.45 (d, *J* = 9.5 Hz), 131.08 (d, *J* = 8.7 Hz), 131.22 (d, *J* = 97.7 Hz), 131.91 (d, *J* = 3.2 Hz) 131.94 (d, *J* = 3.2 Hz), 133.40 (d, *J* = 100.9 Hz); ^31^P NMR (162 MHz, CDCl_3_) δ 37.62; anal. calcd for C_23_H_29_O_2_P: C, 74.98; H, 7.93; found: C, 74.67; H, 8.07.

### (1*R*,2*R*,3*R*,4*R*,5*R*)-3-((Diphenylphosphanyl)methyl)isoverbanol (**23**)

Phosphine oxide **22** (0.221 g, 0.6 mmol) was dissolved in dry toluene (2 mL) in a Schlenk flask under nitrogen. Then, poly(methylhydrosiloxane) (PMHS, 0.3 mL) and titanium(IV) isopropoxide (0.672 g, 0.7 mL, 2.4 mmol) were added dropwise to the solution. The reaction mixture was stirred for 24 h at 100 °C, cooled, and poured into a solution of 48% hydrofluoric acid (3.6 mL) and water (3 mL). The mixture was stirred overnight and the layers were separated. The aqueous layer was extracted with toluene (2 × 10 mL). The combined organic layers were washed with 5% sodium bicarbonate (5 mL) and brine (5 mL). After drying the solution with anhydrous magnesium sulfate and filtration, the solvent was evaporated and the product was purified by flash chromatography on silica gel (hexane/ethyl acetate 80:20). Phosphine **23** (0.131 g, 62%) was obtained as a colorless oil. ^1^H NMR (700 MHz, CDCl_3_) δ 0.93 (s, 3H, CH_3_), 1.14 (d, *J* = 7.7 Hz, 3H, CH_3_), 1.23 (s, 3H, CH_3_), 1.38 (d, *J* = 10.1 Hz, 1H), 1.92–1.96 (m, 1H), 1.98 (m, 1H), 2.06–2.12 (m, 2H), 2.28–2.33 (m, 1H), 2.36–2.40 (m, 1H), 2.41–2.47 (m, 1H), 4.03 (d, *J* = 6.2 Hz, 1H), 7.33–7.39 (m, 6H), 7.46–7.51 (m, 4H); ^13^C NMR (176 MHz, CDCl_3_) δ 16.35 (d, *J* = 3.5 Hz), 23.02, 25.07, 27.97, 30.17 (d, *J* = 11.8 Hz), 35.34 (d, *J* = 6.9 Hz), 39.04 (d, *J* = 11.1 Hz), 39.80, 48.09, 48.88, 77.23, 128.49 (d, *J* = 6.9 Hz), 128.50 (d, *J* = 6.9 Hz), 128.70, 128.75, 128.88 (d, *J* = 11.8 Hz), 132.73 (d, *J* = 18.0 Hz), 132.81 (d, *J* = 18.0 Hz), 138.14 (d, *J* = 9.0 Hz); ^31^P NMR (283.5 MHz, CDCl_3_) δ −18.78; anal. calcd for C_23_H_29_OP: C, 78.38; H, 8.29; found: C, 78.55; H, 8.34.

### Diphenyl (((1*R*,2*S*,5*R*)-δ-pinen-4-yl)methyl)phosphine oxide (**26**)

Unsaturated phosphine oxide **26** was obtained applying the procedure described for **21**. Allylic alcohol **11** (0.333 g, 2 mmol), DMAP (0.280 g, 2,3 mmol), diphenylphosphine chloride (0.441 g, 2 mmol), and toluene (5 mL) were used for the reaction. The crude product was purified on silica gel (eluent: dichloromethane/diethyl ether 10:90) to give **26** (0.547 g, 78%), mp 62–66 °C, 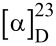 −14 (*c* 2.0, CHCl_3_); ^1^H NMR (700 MHz, CDCl_3_) δ 0.71 (s, 3H, CH_3_), 0.78 (d, *J* = 7.1 Hz, 3H, CH_3_), 0.87 (d, *J* = 9.0 Hz, 1H), 1.18 (s, 3H, CH_3_), 1.71 (m, 1H), 2.06 (dt, *J* = 9.0 Hz, 5.6 Hz, 1H), 2.20 (td, *J* = 5.5, 1.4 Hz, 1H), 2.30 (m, 1H), 3.03–3.13 (m, 2H), 5.16 (m, 1H), 7.42–7.46 (m, 4H), 7.47–7.51 (m, 2H), 7.72–7.78 (m, 4H); ^13^C NMR (176 MHz, CDCl_3_) δ 18.22 (d, *J* = 4.2 Hz) 20.59, 26.31, 27.60 (d, *J* = 2.1), 34.73 (d, *J* = 2.1 Hz), 38.91 (d, *J* = 68.0 Hz), 40.94 (d, *J* = 1.4 Hz), 46.41, 47.73 (d, *J* = 2.8 Hz), 128.27 (d, *J* = 11.8 Hz), 128.44 (d, *J* = 11.8 Hz), 128.83 (d, *J* = 11.8 Hz), 130.87 (d, *J* = 9.0 Hz), 131.17 (d, *J* = 9.0 Hz), 131.49 (d, *J* = 2.8 Hz), 131.54 (d, *J* = 2.1 Hz), 132.95 (d, *J* = 97.8 Hz), 133.70 (d, *J* = 96.4 Hz), 137.43 (d, *J* = 10.4 Hz); ^31^P NMR (283.5 MHz, CDCl_3_) δ 29.93; anal. calcd for C_23_H_27_OP: C, 78.83; H, 7.77; found: C, 78.97; H, 7.51.

### (((1*R*,2*R*,3*S*,4*S*,5*S*)-3-Hydroxypinan-4-yl)methyl)diphenylphosphine oxide (**27**)

Hydroboration–oxidation of **26** was carried out according to the procedure described for **22**. Unsaturated phosphine oxide **26** (0.350 g, 1 mmol) and BMS (0.2 mL, 10 M, 2 mmol) were used in the hydroboration reaction. *meta*-Chloroperbenzoic acid (75%, 0.575 g, 2.5 mmol) was used in the oxidation reaction. The product was purified on silica gel (eluent: dichloromethane/diethyl ether 50:50) to give **27** (0.188 g, 51%). ^1^H NMR (400 MHz, CDCl_3_) δ 0.99 (s, 3H, CH_3_), 1.04 (d, *J* = 7.1 Hz, 3H, CH_3_), 1.12 (d, *J* = 10.3 Hz, 1H), 1.21 (s, 3H, CH_3_), 1.72–1.80 (m, 2H), 2.08–2.20 (m, 2H), 2.31 (ddd, *J* = 15.2, 7.5, 1.8 Hz, 1H), 2.50–2.63 (m, 2H), 4,33 (dd, *J* = 9.8, 4.9 Hz, 1H), 7.43–7.56 (m, 6H), 7.69–7.79 (m, 4H); ^13^C NMR (100 MHz, CDCl_3_) δ 14.23, 23.27, 27.67, 29.54, 35.36, 37.56 (d, *J* = 69.1 Hz), 39.22, 48.38 (d, *J* = 3.2 Hz), 49.03, 49.76 (d, *J* = 13.5 Hz), 71.47 (d, *J* = 1.6 Hz), 128.71 (d, *J* = 5.6 Hz), 128.82 (d, *J* = 5.6 Hz), 130.39 (d, *J* = 9.5 Hz), 131.10 (d, *J* = 9.5 Hz), 131.61 (d, *J* = 98.6 Hz), 131.82 (d, *J* = 3.2 Hz), 131.88 (d, *J* = 2.4 Hz), 133.61 (d, *J* = 100.1 Hz); ^31^P NMR (162 MHz, CDCl_3_) δ 36.03; anal. calcd for C_23_H_29_O_2_P: C, 74.98; H, 7.93; found: C, 75.08; H, 7.77.

## Supporting Information

File 1 General information, experimental procedures and characterization data of the following compounds: **2**, **3**, **5**–**8**, **10**, **11**, and **14–18**.
