# Supplementary material for: Chiral terpene auxiliaries V: Synthesis of new chiral γ-hydroxyphosphine oxides derived from α-pinene
Source: Beilstein J Org Chem. 2019 Oct 22;15:2493–9. doi: 10.3762/bjoc.15.242 (PMC6839555; doi:10.3762/bjoc.15.242)
Supplement: File 1 — General information, experimental procedures and characterization data of the following compounds: 2, 3, 5–8, 10, 11, and 14–18. [file Beilstein_J_Org_Chem-15-2493-s001.pdf]

BEILSTEIN JOURNAL OF ORGANIC CHEMISTRY

Supporting Information

for

Chiral terpene auxiliaries V: Synthesis of new chiral γ-hydroxyphosphine oxides derived from α-pinene

Anna Kmieciak and Marek P. Krzemiński

Beilstein J. Org. Chem. **2019,** *15,* 2493–2499. doi:10.3762/bjoc.15.242

General information, experimental procedures and characterization data of the following compounds: 2, 3, 5–8, 10, 11, and 14–18

License and Terms: This is a supporting information file under the terms of the Creative Commons Attribution License (http://creativecommons.org/licenses/by/4.0). Please note that the reuse, redistribution and reproduction in particular requires that the authors and source are credited. The license is subject to the *Beilstein Journal of Organic Chemistry* terms and conditions: (https://www.beilstein-journals.org/bjoc)

1. General

Analytical methods

Experiments with air- and moisture-sensitive materials were carried out under an argon atmosphere using Schlenk techniques. Glassware was oven dried at 120 ºC, assembled hot, and cooled in a steam of argon. ^{1}H, ^{13}C and ^{31}P NMR spectra were recorded on a Bruker AMX 300 MHz, Avance III 400 MHz, and Avance 700 MHz instruments. Optical rotations were measured on an Optical Activity PolAAr 3000 automatic polarimeter. GC analyses were performed on a Perkin-Elmer Auto System XL chromatograph and HPLC analyses on a Shimadzu LC-10AT chromatograph. Melting points were determined in open glass capillaries and are uncorrected. Elemental analyses were performed on Vario MACRO CHN, ELEMENTAR Analysensysteme GmbH instrument.

Materials

Silica Gel 60, Merck, 230–400 mesh, was used for preparative column chromatography. Macherey-Nagel Polygram Sil G/UV254 0.2 mm plates were used for analytical TLC. Tetrahydrofuran was freshly distilled from sodium benzophenone ketyl. Lead(IV) acetate, diphenyl diselenide, α-pinene, β-pinene, platinum(IV) oxide, cerium(III) chloride heptahydrate, borane-dimethyl sulfide adduct, *meta*-chloroperoxybenzoic acid, sodium methoxide, diisopropyl azadicarboxylate were purchased from commercial sources. (+)-Nopinone was prepared according to the literature [1].

2. Synthetic procedures and analytical data

2.1. (1*R*,5*R*)-(+)-Verbenone (2)

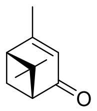

(+)-α-Pinene ($[\alpha]_D^{24} + 47.7$ (c 2.0, $CHCl_3$), 93% ee) (13.62 g, 0.1 mol) and toluene (200 mL) were placed under nitrogen atmosphere in a 500 mL three necked flask equipped with reflux condenser, magnetic stirrer, and thermometer. The solution was heated in an oil bath to 65 °C. Lead(IV) acetate (47.44 g, 0.107 mol) was added over 25 min and the stirring was continued for 75 min. The solution was cooled to room temperature,

filtered through a pad of celite and the precipitate was washed with toluene (4 × 25 mL). Water (150 mL) was added to the filtrate and the mixture was stirred for 1 h. The mixture was filtered through a pad of celite to remove a brown precipitate. The filtrate was transferred to a separatory funnel, layers were separated and the aqueous layer was extracted with diethyl ether (3 × 50 mL). The combined organic layers were dried over anhydrous magnesium sulfate. After filtration, the solvent was removed on rotary evaporator under reduced pressure and then a water-methanol solution of KOH (10%, 75 mL (15 mL of water and 60 mL of methanol)) was added to the residue. The solution was stirred for 20 hours, water (100 mL) was added and the mixture was extracted with diethyl ether (4 × 75 mL). The combined organic layers were dried over anhydrous magnesium sulfate. After filtration, the solvent was removed on rotary evaporator under reduced pressure. The resulting yellow-orange oil and ether (150 mL) were transferred into a two-necked flask equipped with a thermometer, dropping funnel, and a magnetic stirring bar. The solution was cooled to 0 °C and sodium dichromate dihydrate (13.80 g, 46.3 mmol) dissolved in water (53 mL) with concentrated sulfuric acid (6 mL) was added dropwise over 30 minutes. The brown solution was stirred for 1 h at 0 °C, followed by warming to an ambient temperature stirring was continued for 20 h. Water (100 mL) was added and the mixture was transferred to a separatory funnel. Layers were separated, the aqueous layer was extracted with diethyl ether (3 × 100 mL). The combined organic layers were washed with brine (100 mL) and dried over anhydrous magnesium sulfate. After filtration, the solvent was removed on a rotary evaporator. The product was distilled under reduced pressure to yield (+)-verbenone (8.71 g, 58%) as a colorless liquid, bp 75–78 °C / 5.5 mmHg, $[\alpha]_D^{22} = +231.5\ (c\ 2.0, \mathrm{CHCl_3})$, 93% ee.

^{1}H NMR (700 MHz, $CDCl_3$): δ = 1.01 (s, 3H, CH_3), 1.50 (s, 3H, CH_3), 2.04 (d, *J* = 1.5 Hz, 3H, CH_3), 2.08 (d, *J* = 9.3 Hz, 1H), 2.40–2.43 (m, 1H), 2.63–2.66 (td, *J* = 5.9, 1.7 Hz, 1H), 2.78–2.83 (dt, *J* = 9.2, 5.6 Hz, 1H), 5.73 (sxt, *J* = 1.6 Hz, 1H). ^{13}C NMR (176 MHz, $CDCl_3$): δ = 21.99, 23.50, 26.54, 40.78, 49.68, 53.97, 57.56, 121.10, 170.17, 203.91.

2.2. (1*R*,2*S*,5*R*)-(+)-Verbanone (3)

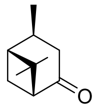

The autoclave was charged with (+)-verbenone (7.51 g, 50 mmol), cyclohexane (9 mL) and platinum(IV) oxide (0.020 g). The autoclave was closed and the air was removed by charging and releasing the hydrogen three times. Then, the autoclave was filled with hydrogen to a pressure of about 10 atm. The mixture was stirred overnight at rt. The reaction mixture was filtered through a pad of celite and cyclohexane was evaporated on a rotary evaporator. The crude product was distilled under reduced pressure to yield (+)-verbanone (7.08 g, 93%) as a colorless liquid, bp 56–58 °C / 0.5 mmHg, $[\alpha]_D^{22} = +59.2\ (c\ 2.0, \mathrm{CHCl_3})$, 93% ee.

^{1}H NMR (700 MHz, $CDCl_3$): δ = 1.01 (s, 3H, CH_3), 1.17 (d, *J* = 7.6 Hz, 3H, CH_3), 1.34 (s, 3H, CH_3), 1.40 (d, *J* = 10.6 Hz, 1H), 2.13–2.14 (m, 1H), 2.16 (dd, *J* = 20.0, 4.8 Hz, 1H), 2.33–2.24 (m, 1H), 2.55 (t, *J* = 5.6 Hz, 1H), 2.57–2.63 (m, 1H), 2.86 (dd, *J* = 20.0, 10.6 Hz, 1H). ^{13}C NMR (176 MHz, $CDCl_3$): δ = 20.9, 24.4, 26.8, 28.3, 30.9, 40.1, 41.3, 47.27, 57.84, 214.2. Anal. calcd for $C_{10}H_{16}O$: C, 78.90; H, 10.59; found: C, 78.34; H, 10.37.

2.3. (1*R*,2*R*,5*R*)-(+)-3-Methyleneverbanone (5)

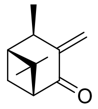

(+)-Verbanone (**3**) (6.09 g, 40 mmol) was added dropwise to a suspension of sodium methoxide (4.32 g, 80 mmol) and ethyl formate (5.93 g, 80 mmol) in toluene (200 mL), and the mixture was stirred at room temperature for 24 h. Hydrochloric acid solution (2 M, 50 mL) was added and the resulting mixture was extracted with diethyl ether (3 × 50 mL). The combined organic layers were washed with brine (50 mL), dried over anhydrous magnesium sulfate, and the solvent was removed on rotary evaporator to give keto aldehyde **4** (5.23 g, 29 mmol).

Keto aldehyde (**4**) (5.23 g, 29 mmol), sodium carbonate (15.9 g), and formaldehyde (37% solution in water, 16 mL) were vigorously stirred in diethyl ether (125 mL) at room temperature for 3 h. Water (150 mL) was then added and the product was extracted with ethyl acetate (2 × 75 mL). The combined organic layers were washed with brine

(50 mL), dried over anhydrous magnesium sulfate, and the solvent was removed on rotary evaporator. The crude product was purified by flash chromatography on silica gel (hexane/ethyl acetate = 90:10). Enone **5** (3.71 g, 78%) was obtained as a colorless oil, $[\alpha]_D^{23} + 25.2\ (c\ 1{,}4;\ \mathrm{CHCl_3})$.

^{1}H NMR (400 MHz, $CDCl_3$): δ = 0.95 (s, 3H, CH3), 1.30–1.32 (d, *J* = 7.3 Hz, 3H), 1.34 (s, 3H, CH3), 1.38–1.40 (d, *J* = 10.3 Hz, 1H), 2.08–2.12 (td, *J* = 5.9, 2.6 Hz, 1H), 2.57–2.65 (m, 2H), 2.85–2.92 (m, 1H), 5.45–5.46 (m, 1H), 6.41–6.42 (m, 1H). ^{13}C NMR (100 MHz, $CDCl_3$): δ = 20.42, 23.68, 27.08, 29.24, 37.81, 40.30, 46.36, 56.06, 122.05, 147.01, 202.20. Anal. calcd for $C_{11}H_{16}O$: C, 80.44; H, 9.82; found: C, 80.11; H, 9.49.

2.4. (1*R*,2*R*,4*S*,5*R*)-3-Methyleneverbanol (6)

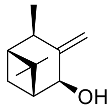

Enone **5** (3.28 g, 20 mmol) and cerium(III) chloride heptahydrate (7.45 g, 20 mmol) were dissolved in methanol (50 mL). The solution was cooled in an ice-water bath and then sodium borohydride (1.14 g, 30 mmol) was added in small portions. The reaction mixture was stirred overnight. Water (100 mL) and dichloromethane (50 mL) were added to the flask and pH of the mixture was adjusted to 5–6 with concentrated hydrochloric acid. Layers were separated and the aqueous layer was extracted with dichloromethane (2 × 50 mL). The combined organic layers were dried over anhydrous magnesium sulfate filtered, and the solvent was removed by rotary evaporation. The crude product was purified by flash chromatography on silica gel (hexane/ethyl acetate = 90:10). Allylic alcohol **6** (2.93 g, 88%) was obtained as a colorless solid, mp 72–74 °C, $[\alpha]_D^{25} + 59.4\ (c\ 1.4;\ \mathrm{CHCl_3})$.

^{1}H NMR (400 MHz, $CDCl_3$): δ = 0.82 (d, *J* = 10.5 Hz, 1H), 1.05 (s, 3H, CH_3), 1.23 (s, 3H, CH_3), 1.25 (s, 3H, CH_3), 1.80 (t, *J* = 5.4 Hz, 1H), 2.06–2.10 (m, 1H), 2.34–2.40 (m, 1H), 2.63–2.67 (m, 1H), 4.50–4.51 (m, 1H), 5.23 (t, *J* = 2.4 Hz, 1H), 5.48 (t, *J* = 2.6 Hz, 1H). ^{13}C NMR (100 MHz, $CDCl_3$): δ = 20.22, 22.86, 28.05, 31.26, 38.62, 40.41, 47.34, 47.66, 75.52, 111.40, 154.96.

Anal. calcd for $C_{11}H_{18}O$: C, 79.46; H, 10.91; found: C, 79.89; H, 10.31.

2.5. (–)-(1*R*,2*R*,3*R*,5*S*)-Isopinocampheol (7)

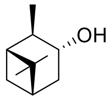

(+)-α-Pinene (17.68 g, 130 mmol) was dissolved in THF (15 mL) under nitrogen atmosphere. The solution was cooled to 0 °C and 10 M borane-dimethyl sulfide adduct (BMS, 5 mL, 50 mmol) was added with stirring. The mixture was allowed to crystallize at 0 °C for 20 h. The supernatant liquid was removed using a syringe with a long needle. The crystalline product was washed with dry hexane (3 × 10 mL) and dried under vacuum for 1 h to give pure Ipc_2BH (12.03 g, 84%).

Ipc_2BH was suspended in THF (40 mL), then water (5 mL) was carefully added followed by the addition of 3M sodium hydroxide solution (16.8 mL, 50.4 mmol). After cooling the mixture to 10 °C, 30% hydrogen peroxide solution (10 mL, 100 mmol) was added dropwise. After addition, the reaction mixture was stirred at room temperature for 30 min and at 50 °C for 1 h. The solution was saturated with sodium chloride and extracted with diethyl ether (3 × 50 mL). The combined organic layers were washed with brine and dried over anhydrous magnesium sulfate. After filtration, the solvent was removed on rotary evaporator to give isopinocampheol **7** (10.11 g, 78%) as a white solid, mp 54–55 °C, $[\alpha]_D^{25} - 34.6\ (c\ 2.0; \mathrm{EtOH})$.

^{1}H NMR (400 MHz, $CDCl_3$): δ = 0.93 (s, 3H, CH_3), 1.04 (d, *J* = 9.8 Hz, 1H), 1.14 (d, *J* = 7.3 Hz, 3H, CH_3), 1.23 (s, 3H, CH_3), 1.55 (br s, 1H), 1.71 (ddd, *J* = 13.9, 4.7, 2.6 Hz, 1H), 1.81 (td, *J* = 5.9, 1.9 Hz, 1H), 1.91–1.97 (m, 2H), 2.35–2.40 (m, 1H), 2.49–2.55 (m, 1H), 4.07 (dt, *J* = 9.5, 4.9 Hz, 1H). ^{13}C NMR (176 MHz, $CDCl_3$): δ = 20.73, 23.68, 27.67, 34.35, 38.15, 38.99, 41.76, 47.67, 47.82, 71.57. Anal. calcd for $C_{10}H_{18}O$: C, 77.87; H, 11.76; found: C, 77.26; H, 11.19.

2.6. (1*R*,2*R*,5*S*)-Isopinocamphone (8)

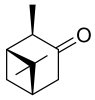

(–)-Isopinocampheol (9.26 g, 60 mmol) was dissolved in diethyl ether (30 mL). The solution of sodium dichromate (5.50 g, 21 mmol) in water (30 mL) and concentrated H_2SO_4 (4.7 mL) was added with vigorous stirring keeping the temperature below 30 °C. The solution was stirred for 2 h, then extracted with diethyl ether (3 × 20 mL). The

combined organic layers were washed with water (15 mL), 5% sodium bicarbonate solution (15 mL), brine (20 mL), and dried over anhydrous magnesium sulfate. After filtration, the solvent was removed on rotary evaporator. The crude product was distilled under reduced pressure to give **8** (7.12 g, 78%) as a colorless liquid, bp 64–66 °C / 4.6 mm Hg, $[\alpha]_D^{22} - 19.2\ (c\ 1.0, \mathrm{EtOH})$.

^{1}H NMR (400 MHz, $CDCl_3$): δ = 0.87 (s, 3H, CH_3), 1.19–1.23 (m, 4H), 1.31 (s, 3H, CH_3), 2.03–2.08 (m, 1H), 2.09–2.15 (m, 1H), 2.47 (m, 1H), 2.53 (dd, *J* = 2.3, 1.1 Hz, 1H), 2.60–2.64 (m, 2H). ^{13}C NMR (100 MHz, $CDCl_3$): δ = 16.68, 21.77, 26.90, 34.25, 38.84, 39.05, 44.59, 44.87, 51.14, 214.69. Anal. calcd for $C_{10}H_{16}O$: C, 78.90; H, 10.59; found: C, 78.37; H, 10.71.

2.7. (1*R*,2*R*,5*R*)-4-Methyleneisopinocamphone (10)

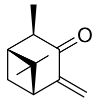

The synthesis of enone **10** was carried out according to procedure described for compound **5**. From **8** (6.09 g, 40 mmol), sodium methoxide (4.32 g, 80 mmol), ethyl formate (5.93 g, 80 mmol), and formaldehyde (37% solution in water, 16 mL), enone **10** (5.45 g, 83%) was obtained as a yellow oil.

^{1}H NMR (700 MHz, $CDCl_3$): δ = 0.81 (s, 3H, CH_3), 1.13 (d, *J* = 7.3 Hz, 3H, CH_3), 1.30 (d, *J* = 10.8 Hz, 1H), 1.35 (s, 3H, CH_3), 1.98 (td, *J* = 6.2, 2.4 Hz, 1H), 2.53–2.58 (m, 1H), 2.66 (qt, *J* = 7.3, 2.3 Hz, 1H), 2.73 (m, 1H), 4.97 (d, *J* = 1.7 Hz, 1H), 5.94 (d, *J* = 1.7 Hz, 1H). ^{13}C NMR (176 MHz, $CDCl_3$): δ = 15.26, 21.10, 26.15, 29.11, 41.08, 44.38, 45.00, 48.59, 117.22, 148.96, 203.40.

Anal. calcd for $C_{11}H_{16}O$: C, 80.44; H, 9.82; found: C, 79.97; H, 9.61.

2.8. (1*R*,2*R*,3*R*,5*R*)-4-Methyleneneoisopinocampheol (11)

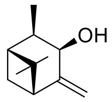

The synthesis of alcohol **11** was carried out according to procedure described for compound **6**. Enone **10** (1.643 g, 10 mmol) was reduced with sodium borohydride

(0.567 g, 15 mmol) in the presence of cerium chloride heptahydrate (3.726 g, 10 mmol). Allylic alcohol **11** (1.396 g, 84%) was obtained as a colorless oil.

^{1}H NMR (400 MHz, $CDCl_3$): δ = 0.76 (s, 3H, CH_3), 1.13 (d, *J* = 6.7 Hz, 3H, CH_3), 1.29 (s, 3H, CH_3), 1.44 (d, *J* = 10.3 Hz, 1H), 1.75–1.78 (m, 2H), 2.18–2.23 (m, 1H), 2.56–2.60 (m, 1H), 4.03 (m, 1H), 4.80 (m, 1H), 5.09 (m, 1H). ^{13}C NMR (100 MHz, $CDCl_3$): δ = 18.73, 21.34, 23.90, 25.93, 39.01, 41.72, 47.42, 51.15, 74.02, 105.77, 154.16. Anal. calcd for $C_{11}H_{18}O$: C, 79.46; H, 10.91; found: C, 79.27; H, 10.58.

2.9. (1*R*,5*R*)-3-Phenylselanylnopinone (14)

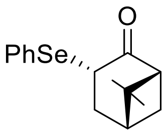

(+)-Nopinone (5.53 g, 40 mmol), selenium dioxide (5.33 g, 48 mmol), diphenyl diselenide (6.87 g, 22 mmol), and methanol (80 mL) were placed in a round-bottom flask. A few drops of sulfuric acid were added to the solution and stirring was continued at room temperature for 5 h. The reaction mixture was diluted with saturated sodium bicarbonate (100 mL) and extracted with ethyl acetate (3 × 100 mL). The combined organic layers were dried with anhydrous magnesium sulfate. The solvent was evaporated using a rotary evaporator under reduced pressure. The crude product was purified by column chromatography on silica gel to remove selenium impurities using hexane/ethyl acetate (90:10) as eluent and then hexane/ethyl acetate (80:20). Phenylselenide **14** (9.033 g) was obtained in 77% yield as a light yellow oil.

^{1}H NMR (700 MHz, $CDCl_3$) δ (ppm): 0.85 (s, 3H, CH_3), 1.36 (s, 3H, CH_3), 1.88 (d, *J* = 10.9 Hz, 1H), 2.20 – 2.25 (m, 2H), 2.53 – 2.61 (m, 2H), 2.70 (t, *J* = 5.3 Hz, 1H), 3.86 (dd, J = 9.5 Hz, 2.0 Hz, 1H), 7.29 – 7.33 (m, 3H), 7.65 – 7.69 (m, 2H). ^{13}C NMR (100 MHz, $CDCl_3$) δ (ppm): 21.97, 25.62, 25.79, 31.53, 40.34, 40.69, 42.07, 57.72, 128.15, 129.05 (2xCH), 130.46 (C), 134.62 (2xCH), 209.81.

2.10. (1*R*,5*R*)-Apoverbenone (15)

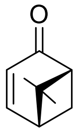

Phenyl selenide **14** (5.866 g, 20 mmol) dissolved in dichloromethane (100 mL) was placed in a round-bottom flask. Pyridine (4.84 mL, 60 mmol) was added to the solution followed by 30% H_2O_2 (4.0 mL, 40 mmol). The mixture was stirred for 30 minutes at room temperature. The reaction mixture was diluted with saturated sodium bicarbonate solution (150 mL). The two-phase mixture was transferred to a separatory funnel and separated. The aqueous layer was extracted with dichloromethane (3 × 100 mL). Organic layers were combined and dried with anhydrous magnesium sulfate. The solvent was evaporated on a rotary evaporator. The product was purified on silica gel by column chromatography (eluent: hexane: ethyl acetate 90:10) to obtain 1.770 g of a light yellow oil (65% yield), $[\alpha]_D^{23} + 284.5\ (c\ 1.1, \mathrm{CHCl_3})$.

^{1}H NMR (700 MHz, $CDCl_3$) δ (ppm): 1.04 (s, 3H, CH_3), 1.52 (s, 3H, CH_3), 2.14 (d, *J* = 9.2 Hz, 1H), 2.60 (m, 1H), 2.72 (td, *J* = 6.0 Hz, 1.8 Hz, 1H), 2.84 (dtd, *J* = 9.2 Hz, 5.5 Hz, 0.9 Hz, 1H), 5.95 (m, 1H), 7.52 (dd, *J* = 8.6 Hz, 6.7 Hz, 1H). ^{13}C NMR (176 MHz, $CDCl_3$) δ (ppm): 22.20, 26.44, 41.86, 43.75, 54.86, 58.57, 125.57, 156.94, 203.93.

2.11. (1*R*,4*R*,5*R*)-(–)-Apopinenol (16)

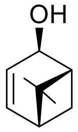

The reduction of enone **15** was carried out according to the procedure described for compound **6**. Enone **15** (1.362 g, 10 mmol) was reduced with sodium borohydride (0.416 g, 11 mmol) in the presence of cerium chloride heptahydrate (5.589 g, 15 mmol). Allylic alcohol **16** (1.313 g, 95%) was obtained as a crystalline product (m.p. 79-82 °C). GC analysis has shown the presence of two diastereoisomeric alcohols: (1*R*,4*R*,5*R*)-**16**: t_r = 8,387 (92,01%); (1*R*,4*S*,5*R*)-**18**: tr = 8,622 (7,99%).

Purification of an analytical sample of this mixture gave pure **16** with $[\alpha]_D^{21} - 21.2\ (c\ 1.0, CHCl_3)$. Lit. [2]: $[\alpha]_D^{25} - 22.5\ (c\ 1.0, C_6H_6)$.

^{1}H NMR (700 MHz, $CDCl_3$) δ (ppm) : 1.10 (s, 3H), 1.34 (s, 3H), 1.37 (d, *J* = 9.03 Hz, 1H), 1.76 (s, 1H, OH), 2.15 (q, *J* = 5.9 Hz, 1H), 2.32 (tdd, *J* = 5.9 Hz, 3.5 Hz, 2.2 Hz, 1H), 2.48 (dddd, *J* = 9.1 Hz, 6.2 Hz, 5.4 Hz, 0.6 Hz, 1H), 4.50 (m, 1H), 5.69 (m, 1H), 6.31 (ddt, *J* = 8.7 Hz, 6.5 Hz, 1.0 Hz, 1H). ^{13}C NMR (176 MHz, $CDCl_3$) δ (ppm): 22.67; 26.52; 36.35; 38.38; 42.00; 48.41; 73.66; 126.04; 137.88.

2.12. (1*R*,4*S*,5*R*)-(+)-Apopinenol (18)

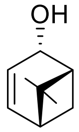

Allylic alcohol **16** (0.691 g, 5 mmol), *p*-nitrobenzoic acid (3.359 g, 20.1 mmol), triphenylphosphine (5.232 g, 19.95 mmol) and tetrahydrofuran (40 mL) were added to the flask under an inert gas atmosphere. The flask was placed in an ice-water bath and diisopropyl azadicarboxylate (4.054 g, 3.95 mL, 20.05 mmol) was added dropwise at a rate such that the temperature of the solution did not exceed 10 °C. The ice-water bath was removed and the solution was stirred overnight at room temperature followed by a further 3 hours at 40 °C. The reaction mixture was cooled to room temperature, diethyl ether (40 mL) was added, and washed with sodium hydrogen carbonate solution (2 × 25 mL). The aqueous layers were combined and washed with diethyl ether (25 mL). The combined organic layers were dried over anhydrous magnesium sulfate. Solvents were evaporated under reduced pressure on a rotary evaporator. The residue was purified by flash column chromatography on silica gel using hexane/ethyl acetate (90:10) as an eluent to obtain *p*-nitro-benzoate ester **17** (1.092 g, 76%) as a mixture of 4*R*:4*S* diastereoisomers in the ratio of 79:21 from ^{1}H NMR spectra. It was not possible to separate these diastereoisomers by TLC analysis using different elution systems.

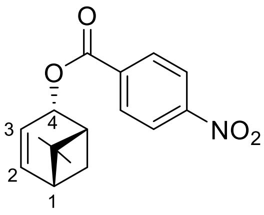

4*R*-ester

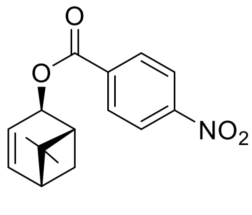

4*S*-ester

17: ^{1}H NMR (700 MHz, $CDCl_3$) δ (ppm): 1.03 (s, 3H, 4*R*), 1.18 (s, 3H, 4*S*), 1.40 (s, 3H, 4*R* + 4*S*), 1.57 (d, 1H, J=9.25 Hz, 4*S*), 1.60-1.63 (m, 1H, 4*R*), 2.26-2.30 (m, 1H, 4*S*), 2.30-2.34 (m, 1H, 4*R*), 2.40-2.43 (m, 2H, 4*R*), 2.48-2.52 (m, 1H, 4*S*), 2.59-2.63 (m, 1H, 4*S*), 5.69 (m, 1H, 4*R*), 5.74-5.77 (m, 1H, 4*R* + 4*S*), 5.86 (m, 1H, 4*S*), 6.52 (m, 1H, 4*S*), 6.56 (dd, J=8.60, 6.20 Hz, 1H, 4*R*), 8.18-8.22 (m, 2H, 4*R* + 4*S*), 8.26-8.30 (m, 2H, 4*R* + 4*S*).

The obtained mixture of esters was dissolved in THF (30 mL) and 5% NaOH aqueous solution (20 mL) was added. The solution was stirred at room temperature to disappear the starting material on TLC analysis (≈5h). Diethyl ether (40 mL) was added, the organic layer was separated, and the aqueous layer was extracted with diethyl ether (2 × 15 mL). The organic layers were combined, washed with brine (15 mL), and dried with anhydrous magnesium sulfate. Solvents were evaporated on a rotary evaporator under reduced pressure to obtain 0.431 g (82%) of the product. GC analysis has shown the existence of two diastereomeric alcohols: (1*R*,4*S*,5*R*)-**18**: t_r = 9,950 (79,45%) and (1*R*,4*R*,5*R*)-**16**: t_r = 9,692 (20,54%).

Purification of an analytical sample of this mixture by column chromatography on silica gel with hexane/diethyl ether 70/30 as an eluent gave pure **18** as a colorless oil with $[\alpha]_D^{20} + 37.2\ (c\ 1.3, \mathrm{CHCl_3})$. Lit. [2]: $[\alpha]_D^{25} + 14.7\ (c\ 1.0, \mathrm{C_6H_6})$.

^{1}H NMR (700 MHz, $CDCl_3$) δ (ppm): 0.91 (s, 3H), 1.33 (d, *J* = 9.03 Hz, 1H), 1.34 (s, 3H), 1.53 (s, 1H, OH), 2.19-2.23 (m, 2H), 2.29 (dtd, *J* = 9.1Hz, 5.5 Hz, 0.6 Hz, 1H), 4.33 (m, 1H), 5.65-5.67 (m, 1H), 6.35-6.37 (m, 1H). ^{13}C NMR (100 MHz, $CDCl_3$) δ (ppm): 20.90; 26.69; 29.28, 42.71;46.77; 47.56; 70.75; 125.94; 139.45.

3. Literature

[1] Binder, C. M.; Bautista, A.; Zaidlewicz, M.; Krzemiński, M. P.; Oliver, A.; Singaram, B. *J. Org. Chem.* **2009**, *74*, 2337-2343.

[2] Nishino, C.; Takayanagi, H. *Agric. Biol. Chern.* **1980**, *44*, 1649-1652.

Chromatograms of (–)-(1*R*,4*R*,5*R*)-apopinenol (**16**) and (+)-(1*R*,4*S*,5*R*)-apopinenol (**18**)

GC analysis: column: Phenomenex ZB-WAX, 15 m x 0.53 mm x 1.00 μm

Initial temperature 60 ºC

Initial time 2 min

Rate 10 ºC/min

Final temperature 240 ºC

NUMER PIKU	CZAS RETENCJI [min]	POWIERZCHNIA [μV 1/100 min]	UDZIAŁ % POWIERZCHNI
A/1	8,387	185061	92,009
A/2	8,622	16072	7,991
SUMA		201133	

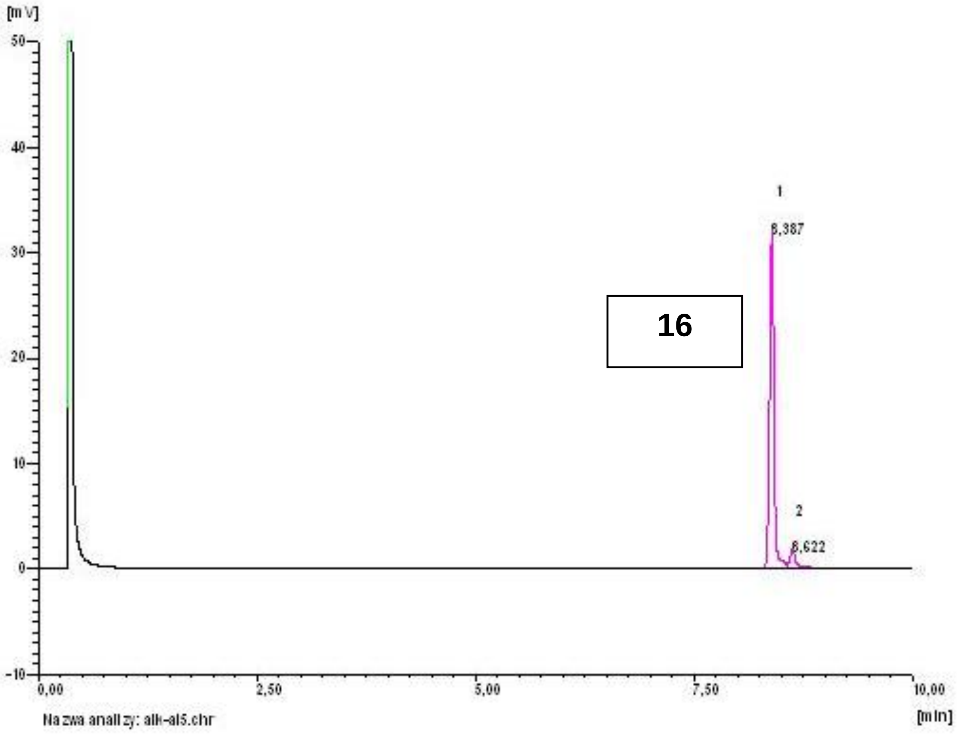

NUMER PIKU	CZAS RETENCJI [min]	POWIERZCHNIA [µV 1/100 min]	UDZIAŁ % POWIERZCHNI
A/1	8,375	23944	20,235
A/2	8,625	94386	79,765
SUMA		118330	

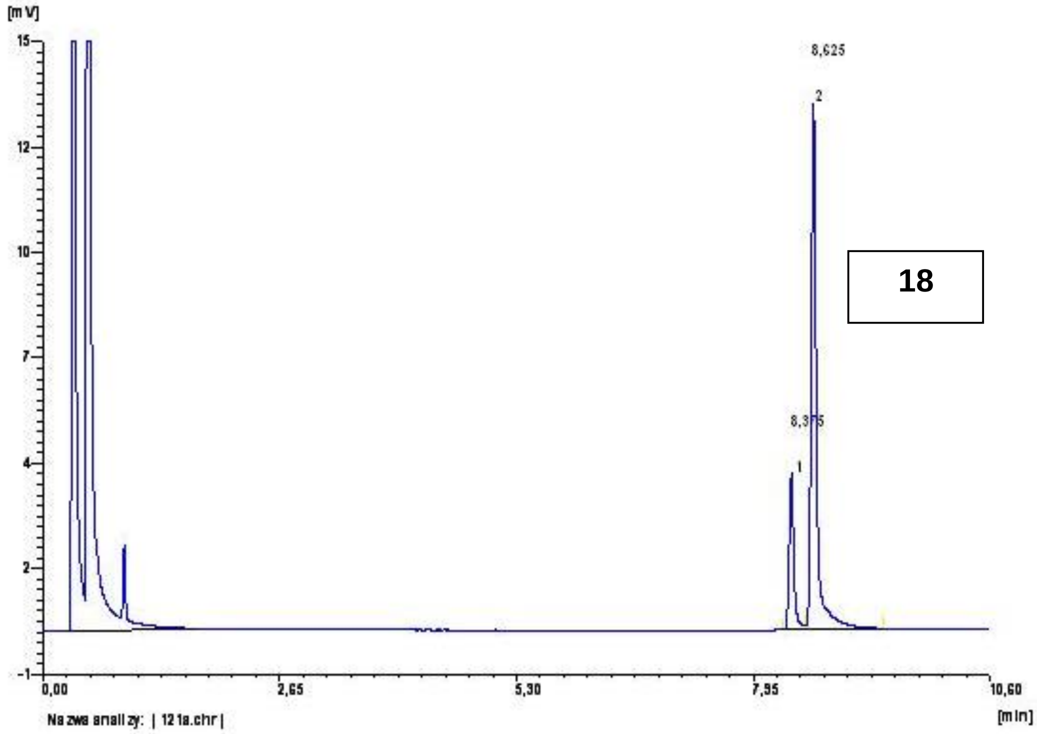

18

(1*R*,5*R*)-(+)-Verbenone (2)

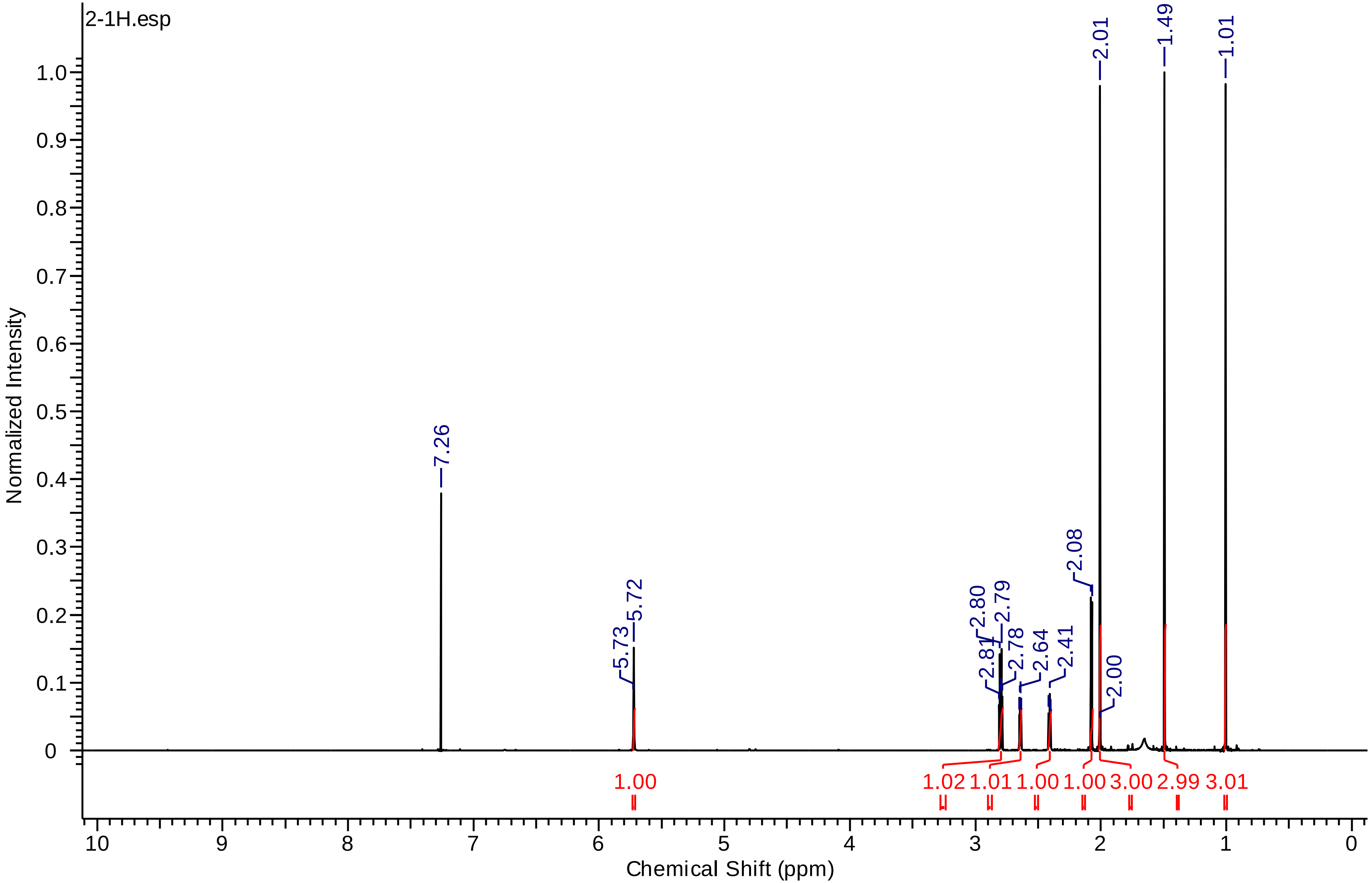

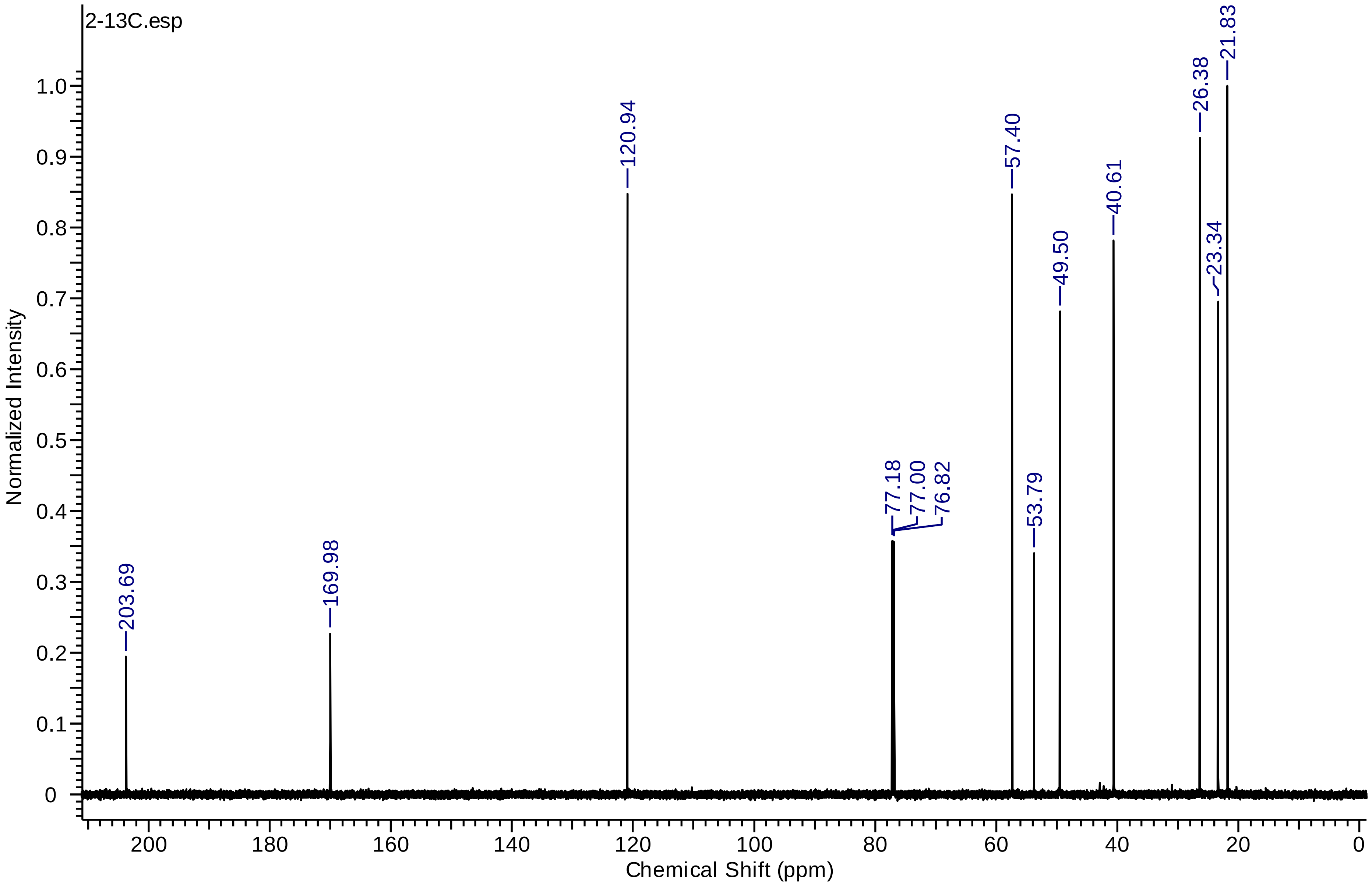

2-13C.esp
Normalized Intensity
Chemical Shift (ppm)
203.69
169.98
120.94
77.18
77.00
76.82
57.40
53.79
49.50
40.61
26.38
23.34
21.83

(1*R*,2*S*,5*R*)-(+)-Verbanone (3)

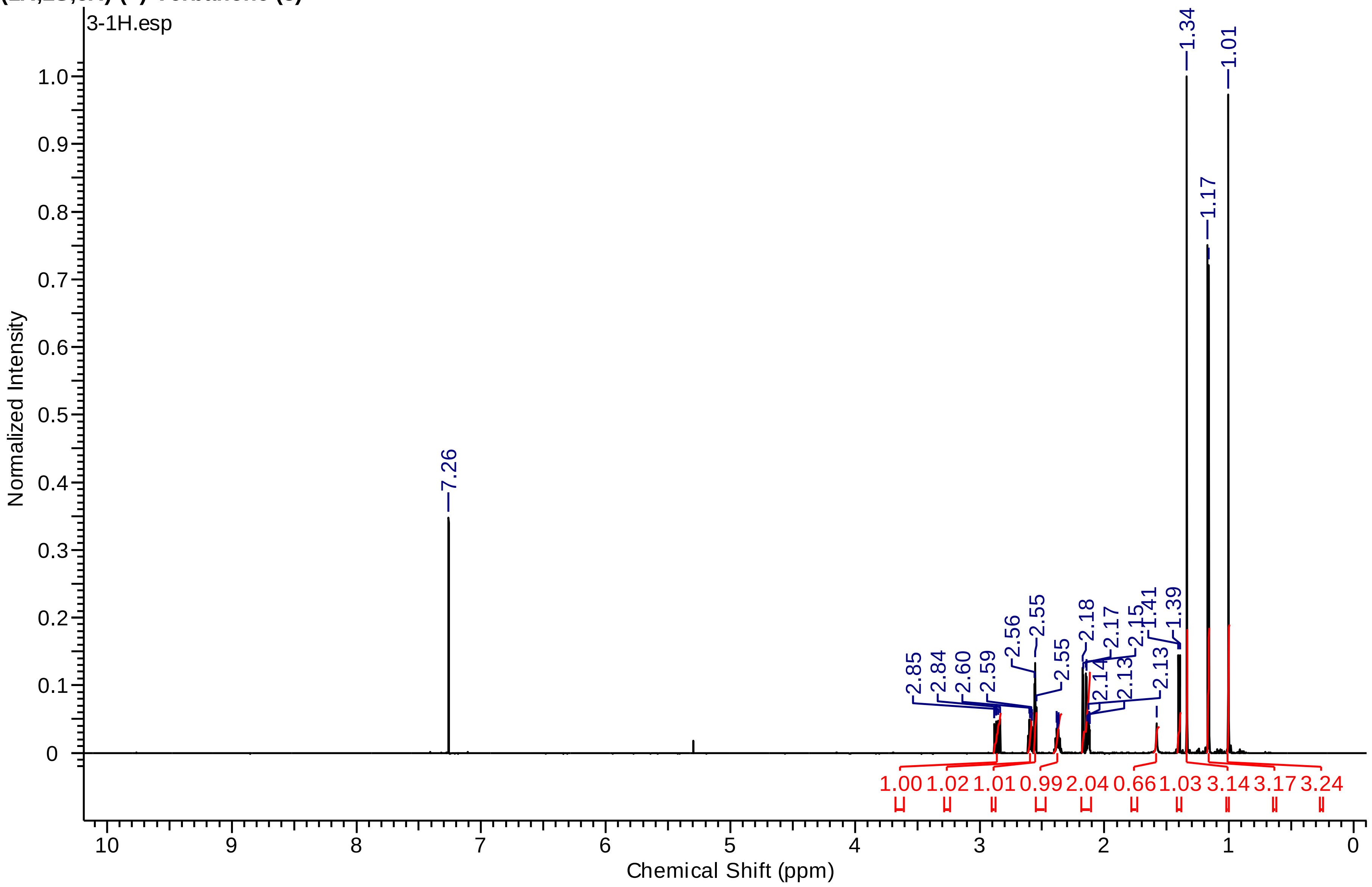

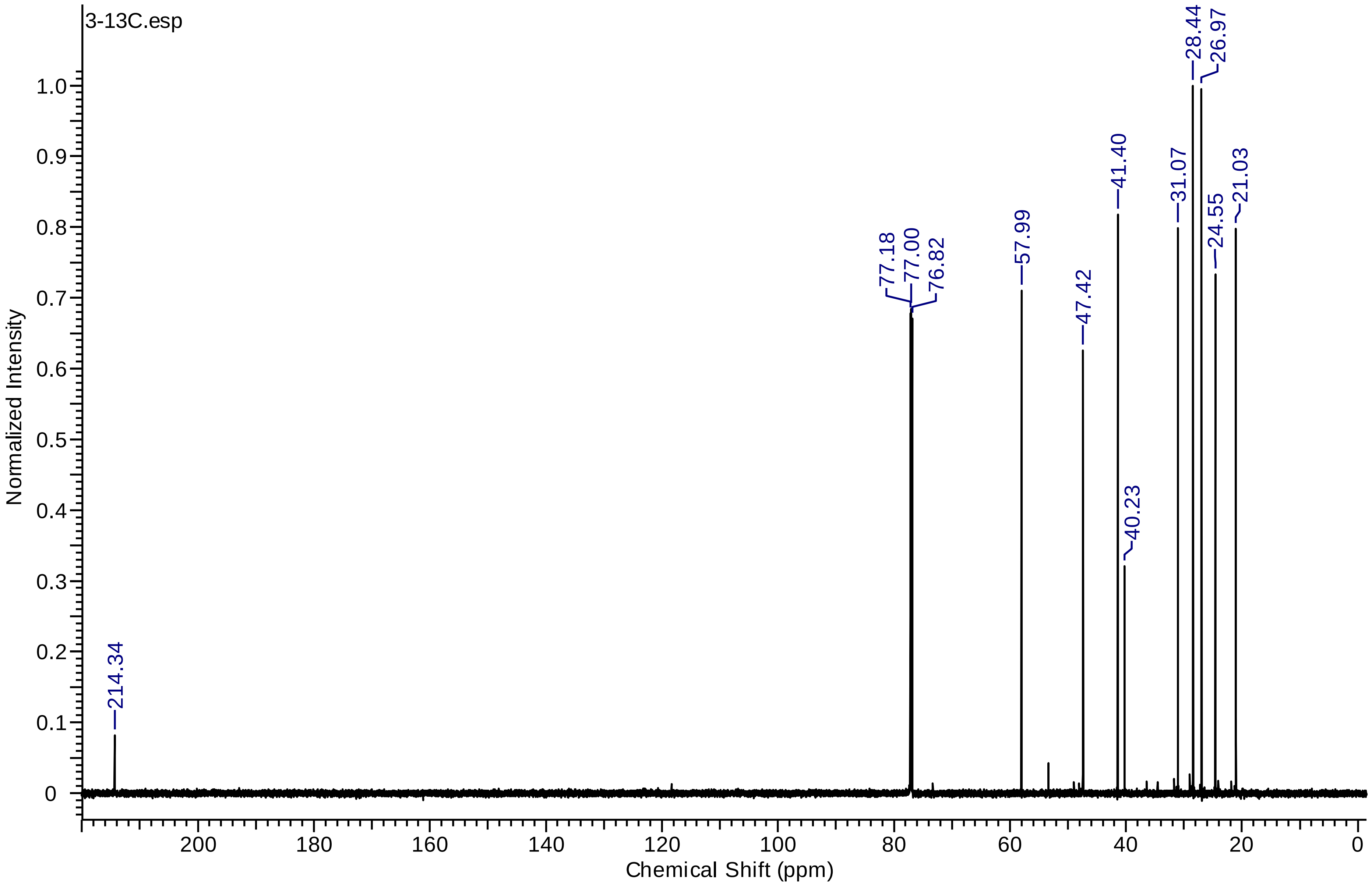

3-13C.esp
Normalized Intensity
Chemical Shift (ppm)
214.34
77.18
77.00
76.82
57.99
47.42
41.40
40.23
31.07
28.44
26.97
24.55
21.03

(1*R*,2*R*,5*R*)-(+)-3-Methyleneverbanone (5)

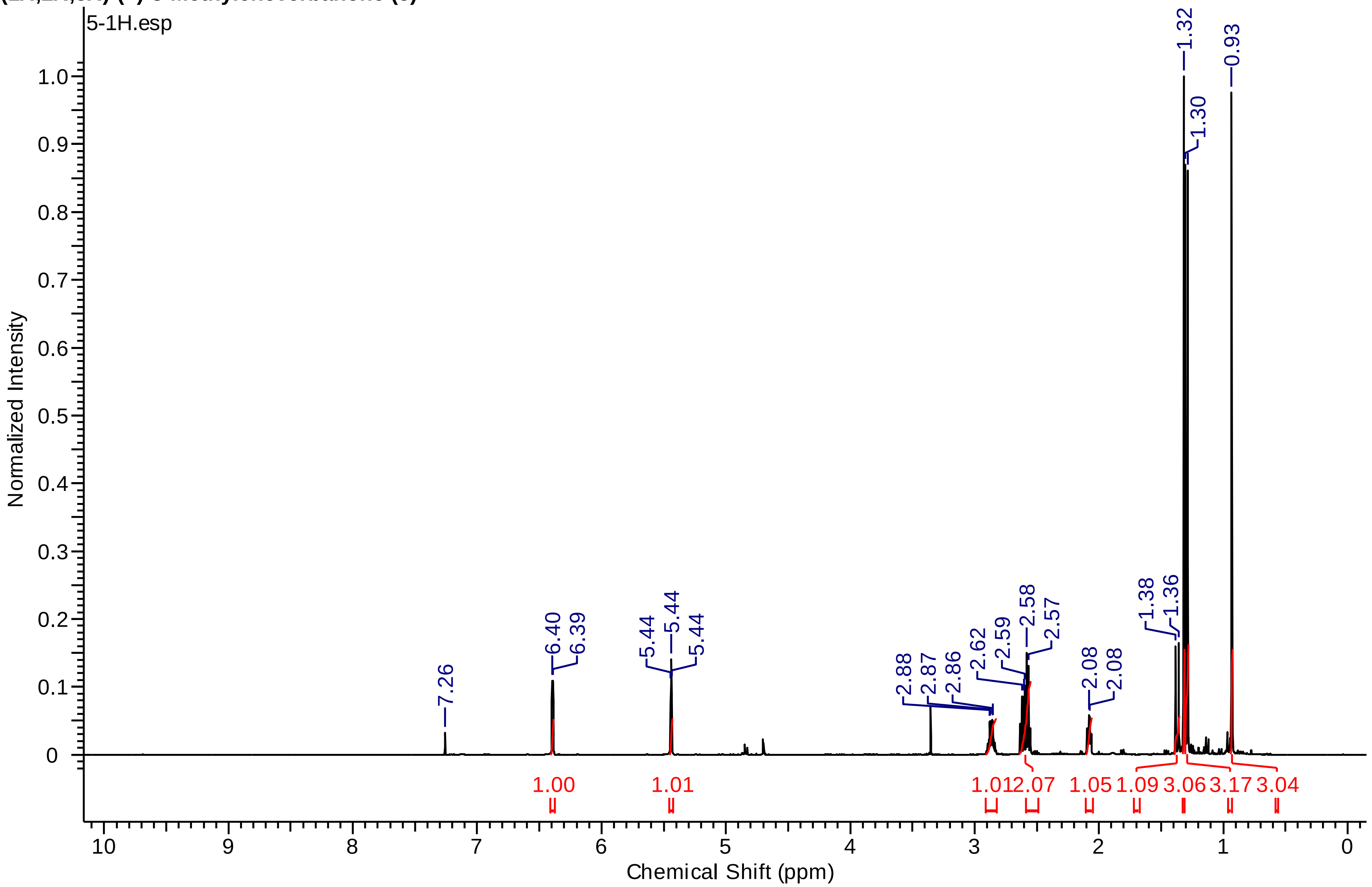

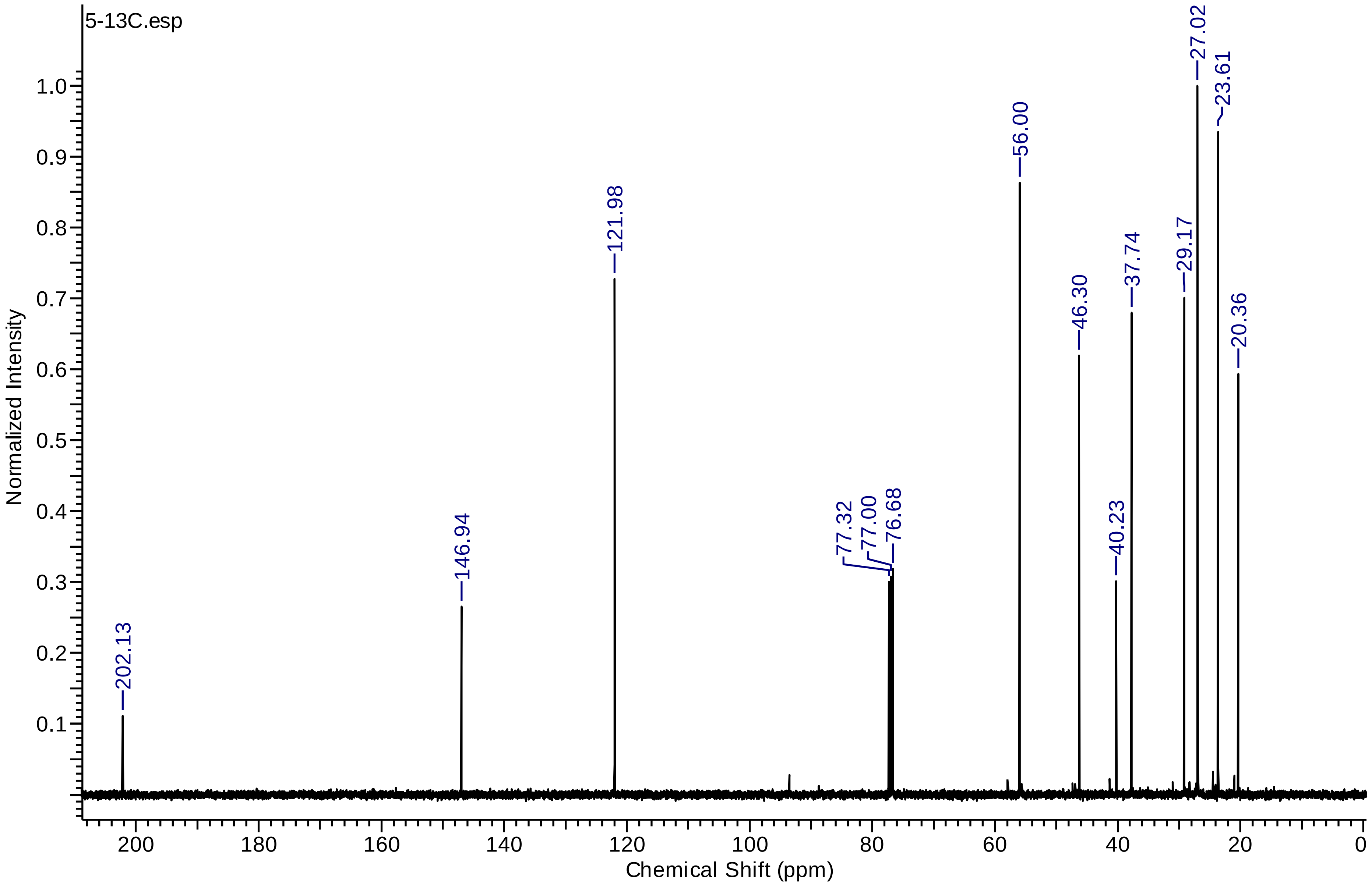
5-13C.esp
Normalized Intensity
Chemical Shift (ppm)
202.13
146.94
121.98
77.32
77.00
76.68
56.00
46.30
40.23
37.74
29.17
27.02
23.61
20.36

(1*R*,2*R*,4*S*,5*R*)-3-Methyleneverbanol (6)

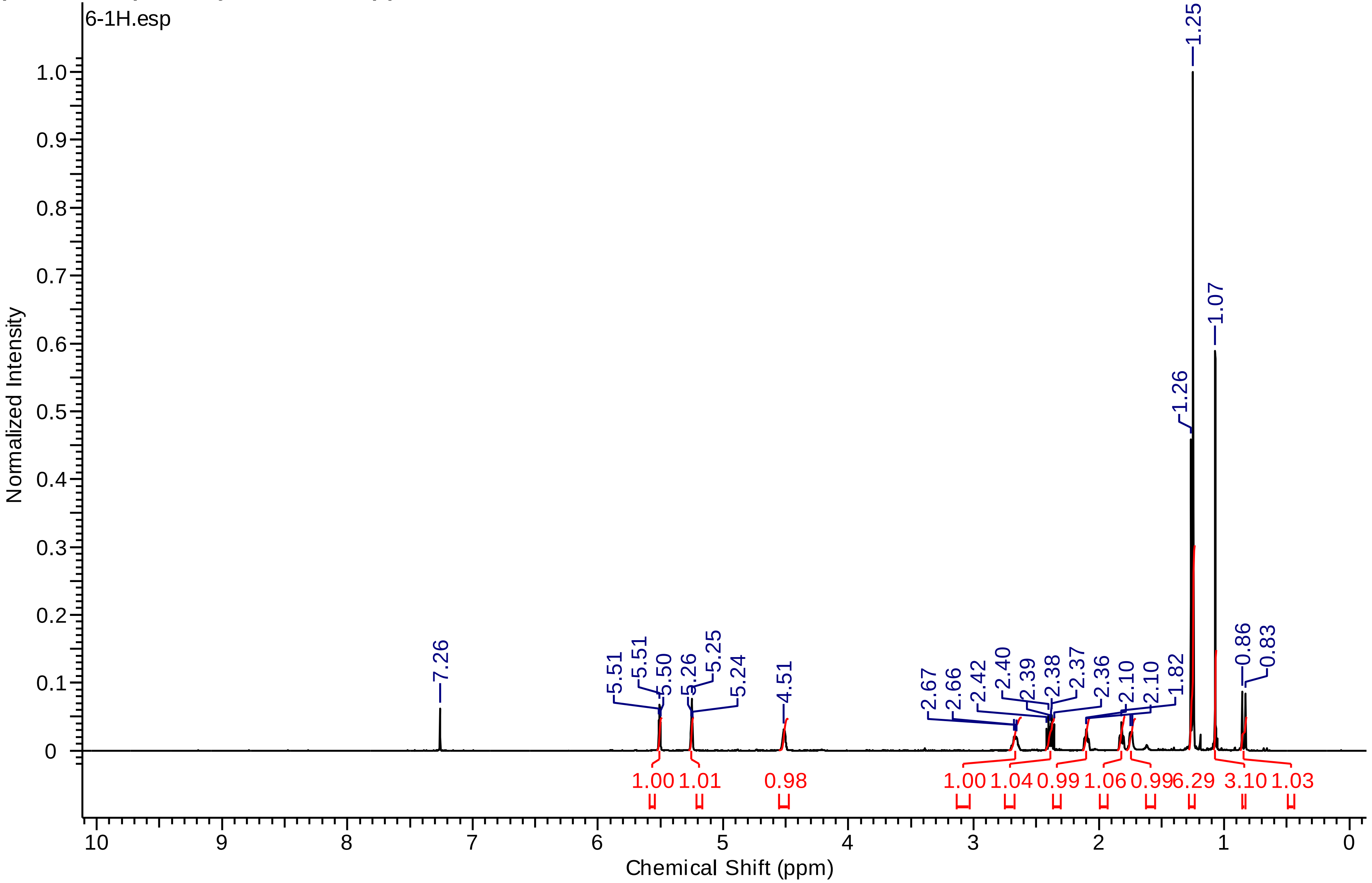

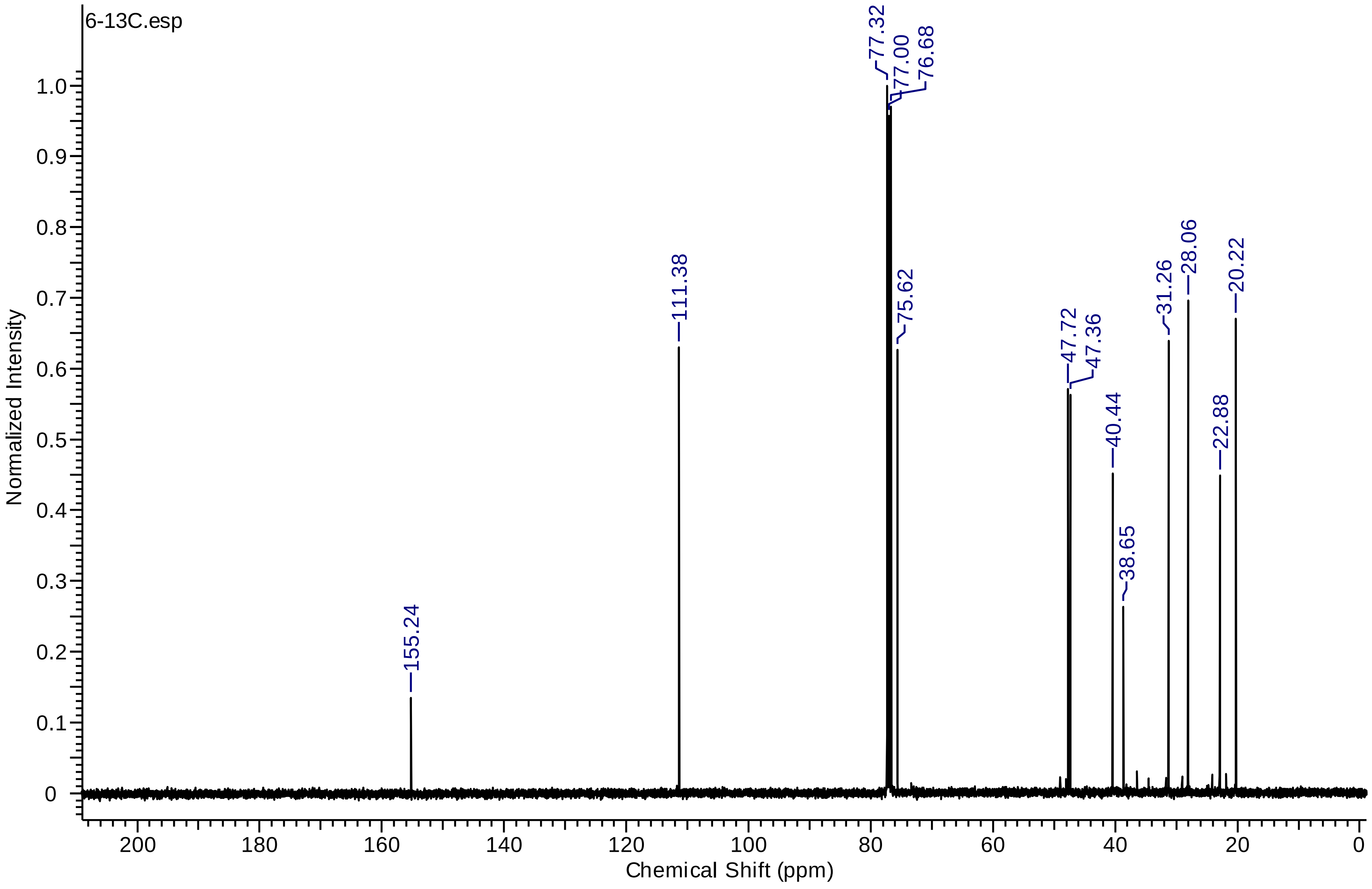
6-13C.esp
Normalized Intensity
Chemical Shift (ppm)
155.24
111.38
77.32
77.00
76.68
75.62
47.72
47.36
40.44
38.65
31.26
28.06
22.88
20.22

(–)-(1*R*,2*R*,3*R*,5*S*)-Isopinocampheol (7)

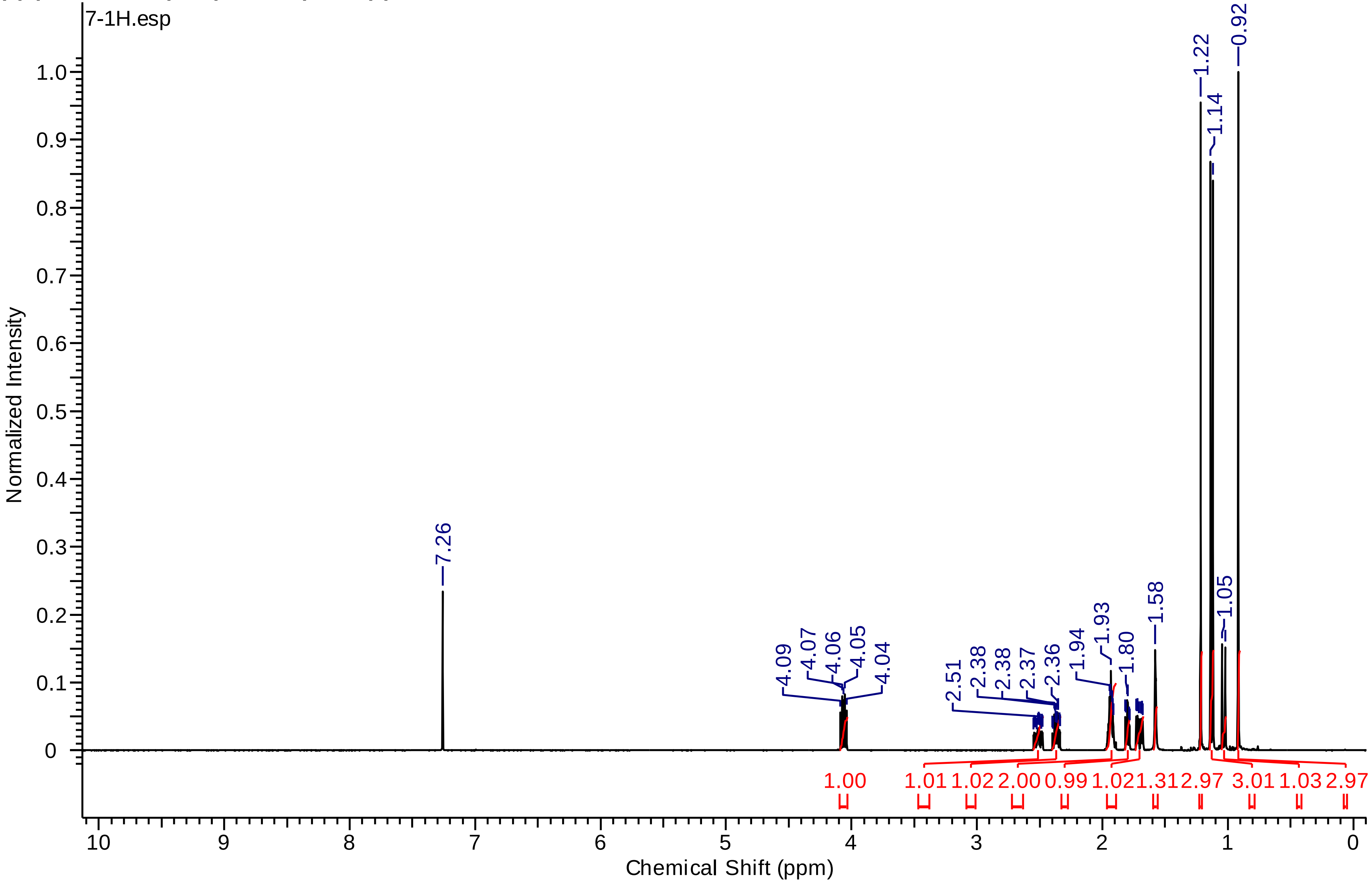

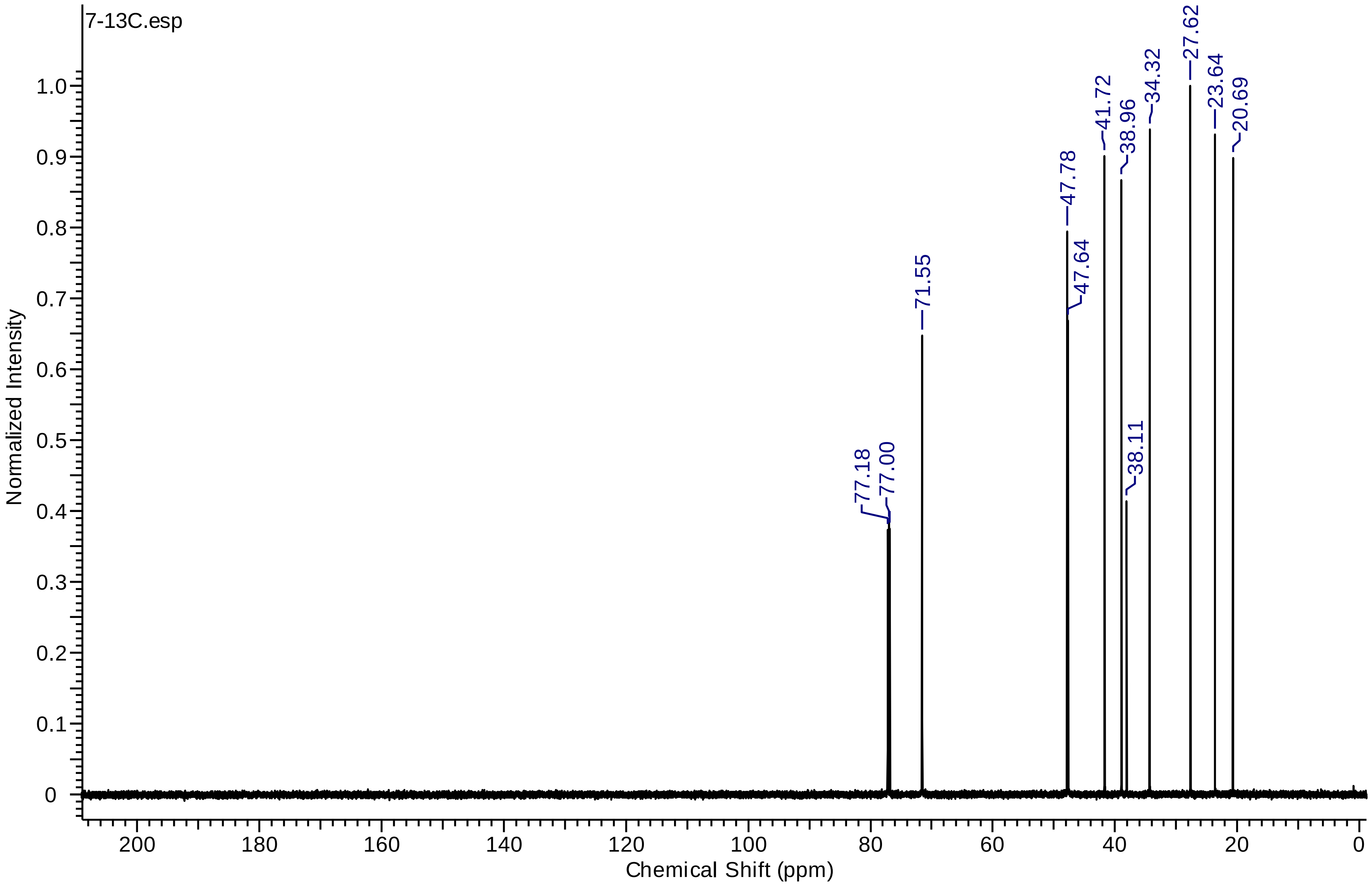

7-13C.esp
77.18
77.00
71.55
47.78
47.64
41.72
38.96
38.11
34.32
27.62
23.64
20.69
Normalized Intensity
Chemical Shift (ppm)

(1*R*,2*R*,5*S*)-Isopinocamphone (8) (contains 12% of unoxidezed isopinocampheol **7**)

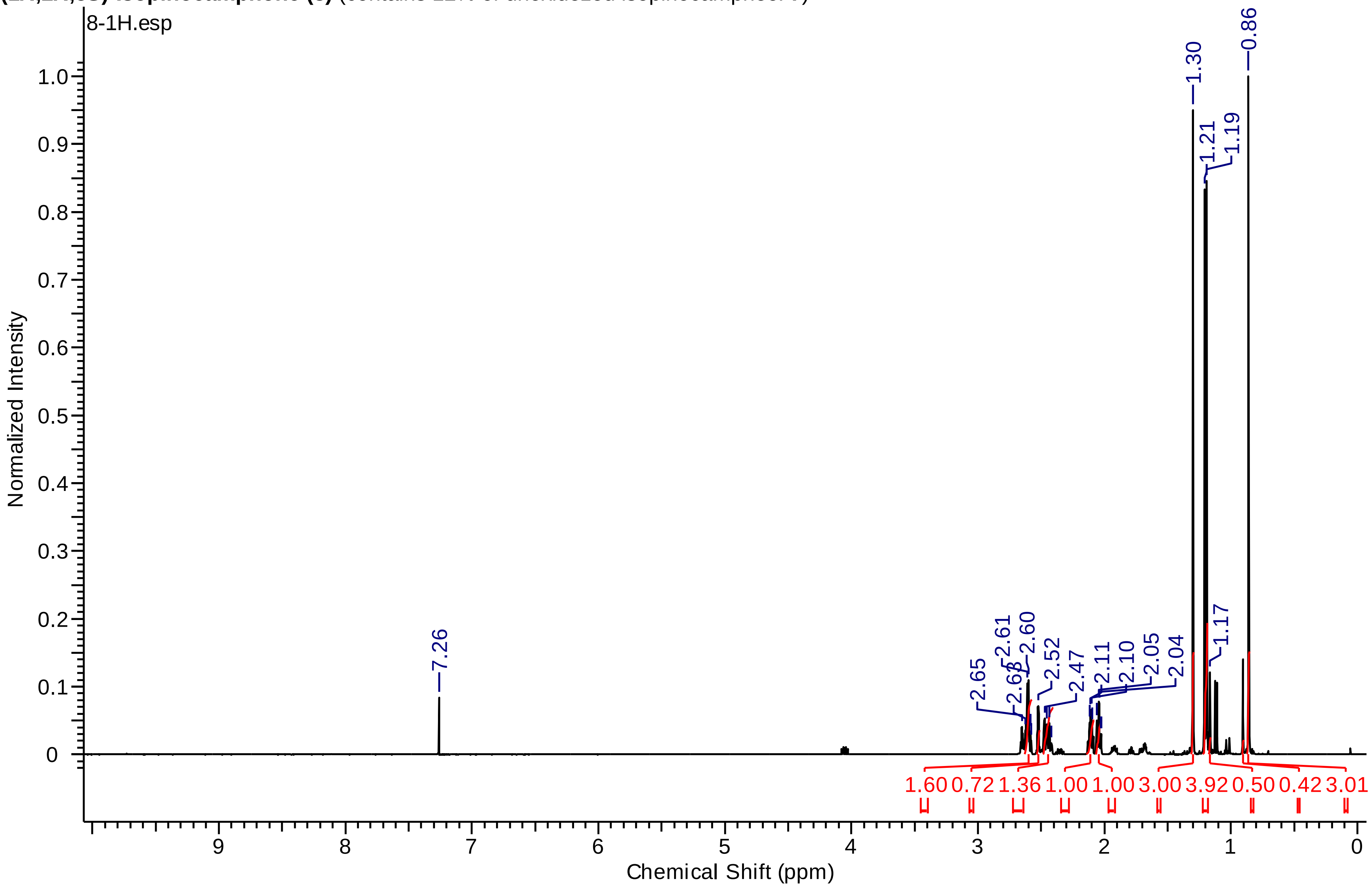

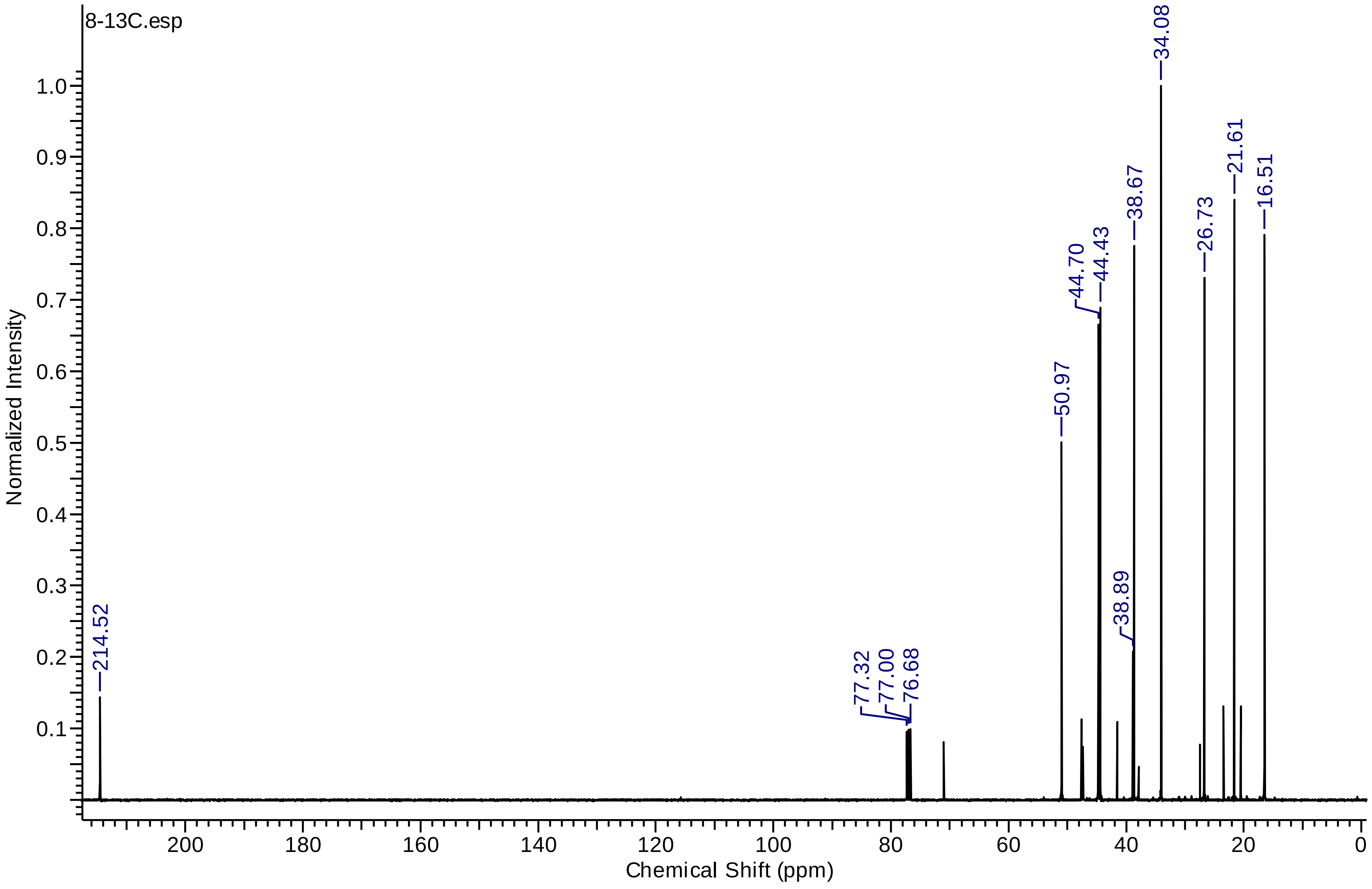
8-13C.esp
214.52
77.32
77.00
76.68
50.97
44.70
44.43
38.89
38.67
34.08
26.73
21.61
16.51
Normalized Intensity
Chemical Shift (ppm)

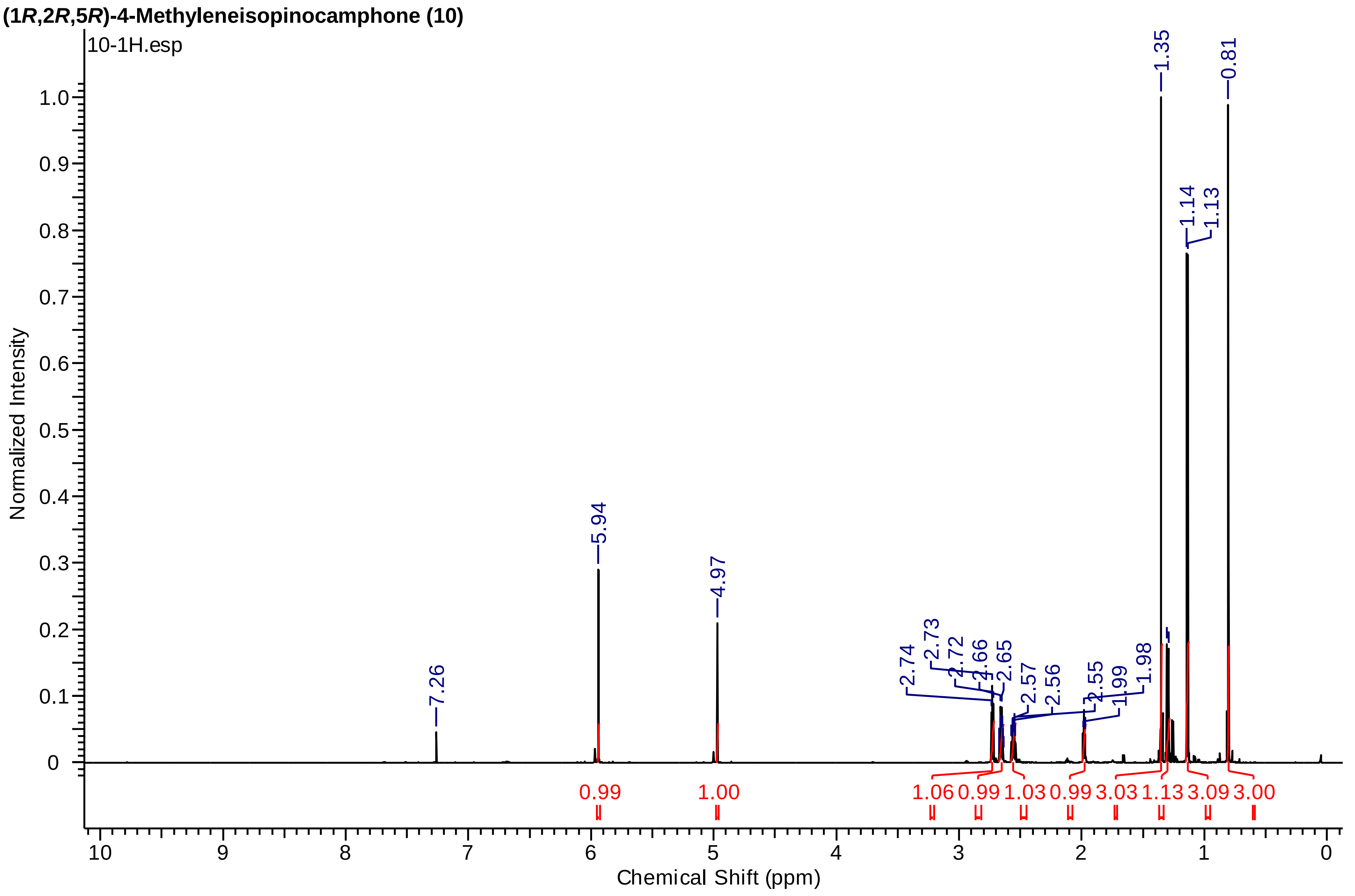

(1R,2R,5R)-4-Methyleneisopinocamphone (10)
10-1H.esp
Normalized Intensity
Chemical Shift (ppm)
7.26
5.94
4.97
2.74
2.73
2.72
2.66
2.65
2.57
2.56
2.55
1.99
1.98
1.35
1.14
1.13
0.81
0.99
1.00
1.06
0.99
1.03
0.99
3.03
1.13
3.09
3.00

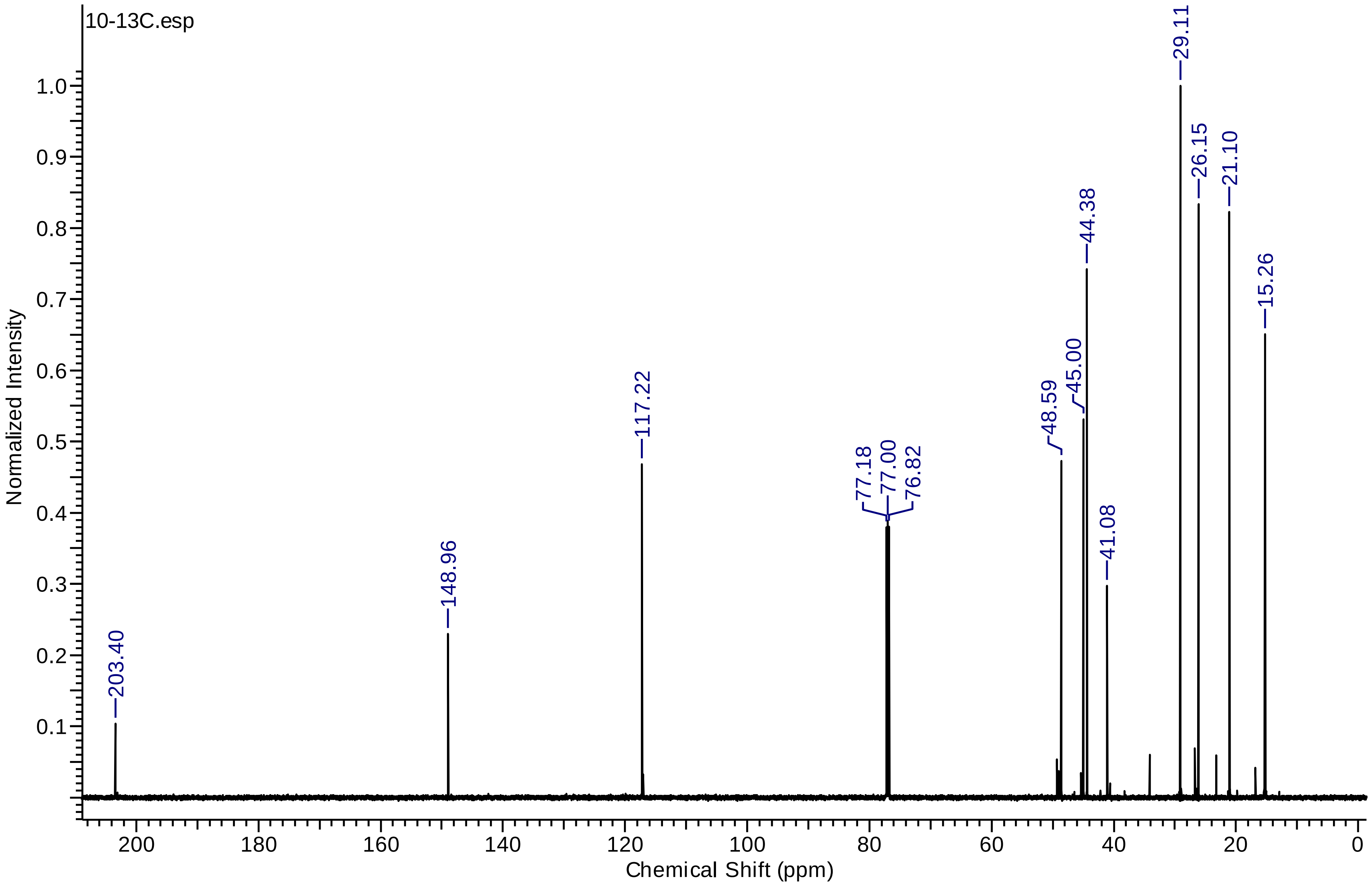
10-13C.esp
203.40
148.96
117.22
77.18
77.00
76.82
48.59
45.00
44.38
41.08
29.11
26.15
21.10
15.26
Normalized Intensity
Chemical Shift (ppm)

(1*R*,2*R*,3*R*,5*R*)-4-Methyleneneoisopinocampheol (11)

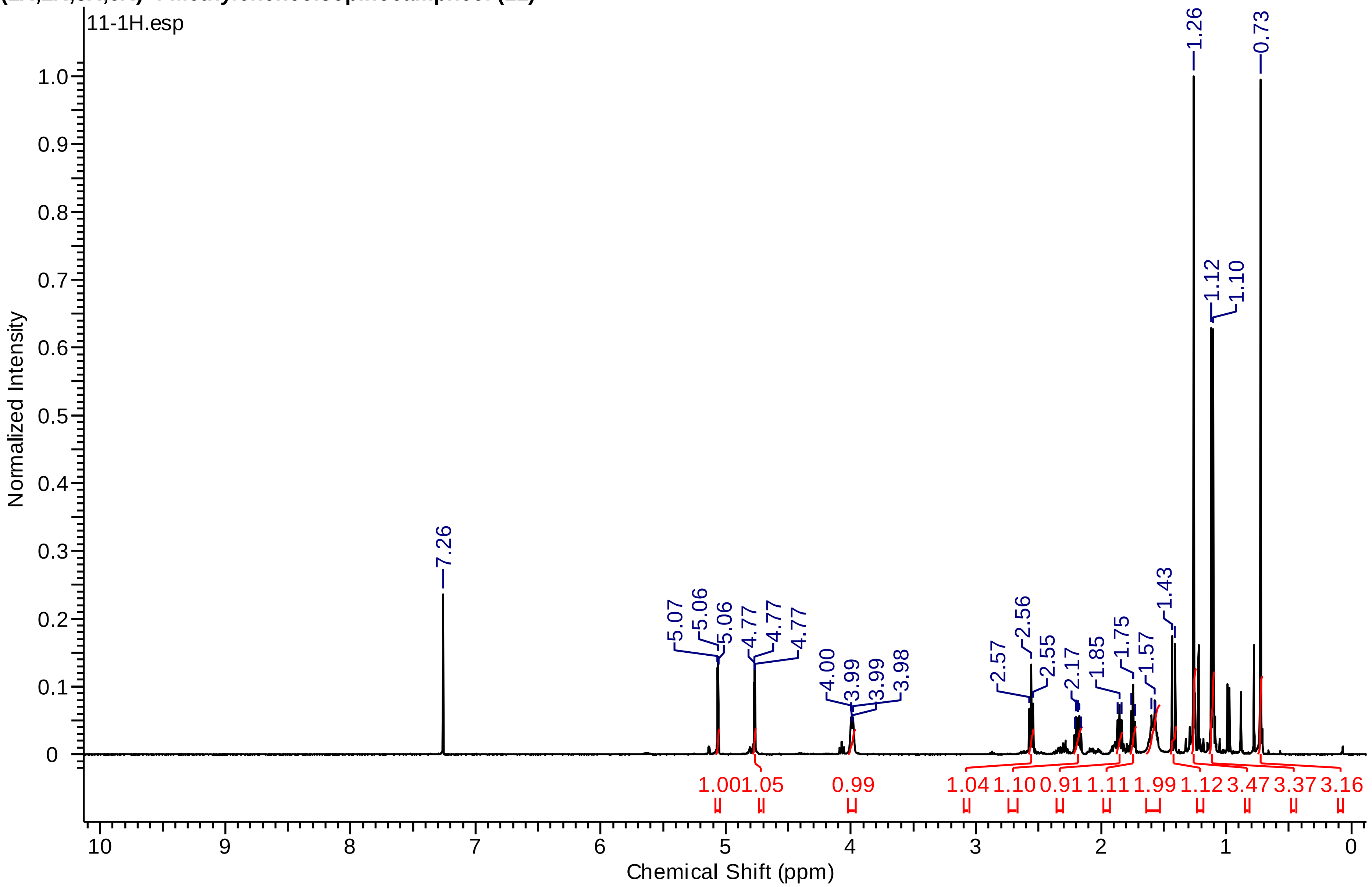

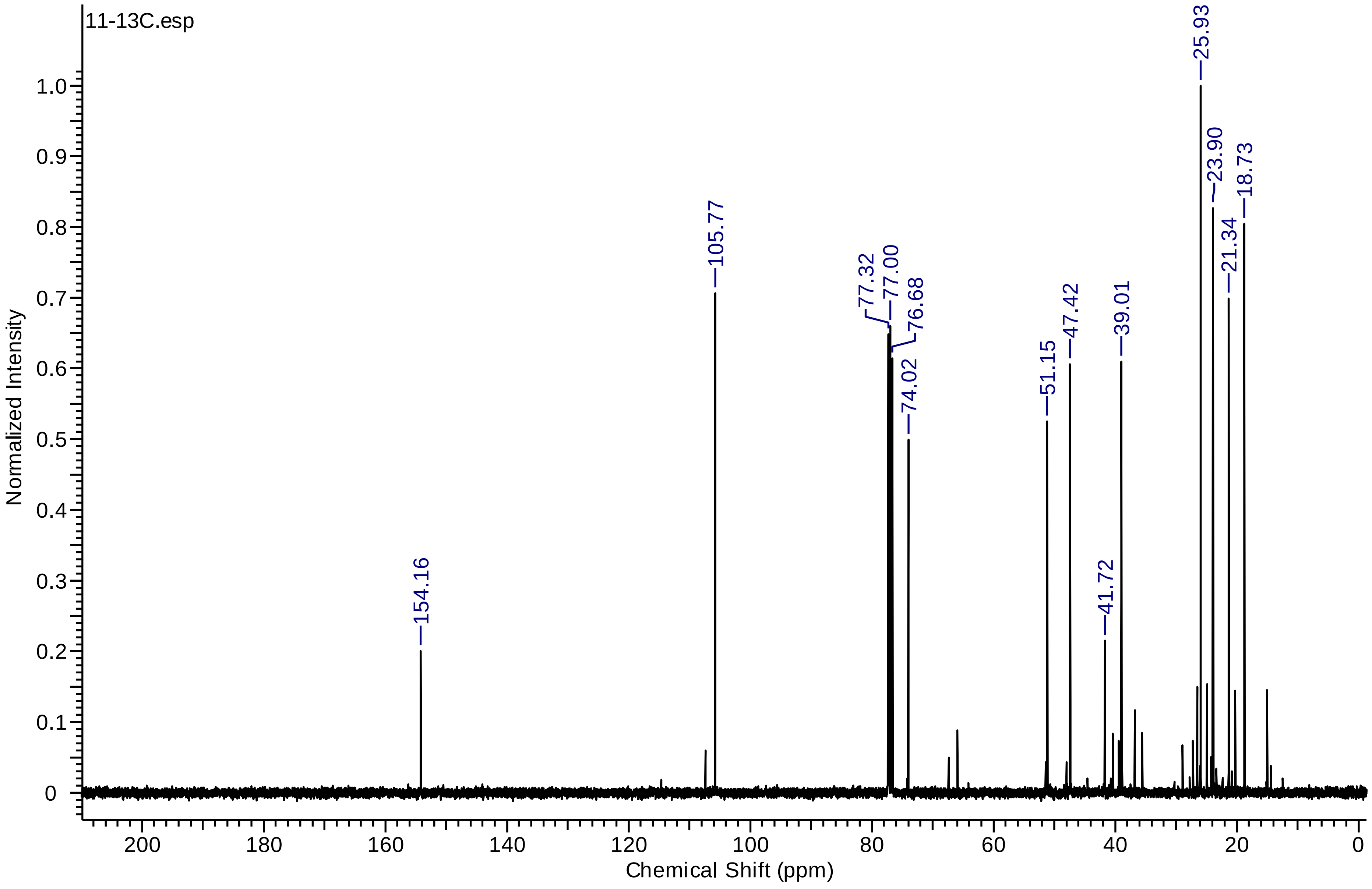
11-13C.esp
154.16
105.77
77.32
77.00
76.68
74.02
51.15
47.42
41.72
39.01
25.93
23.90
21.34
18.73
Normalized Intensity
Chemical Shift (ppm)

(1*R*,5*R*)-3- Phenylselanylnopinone (14)

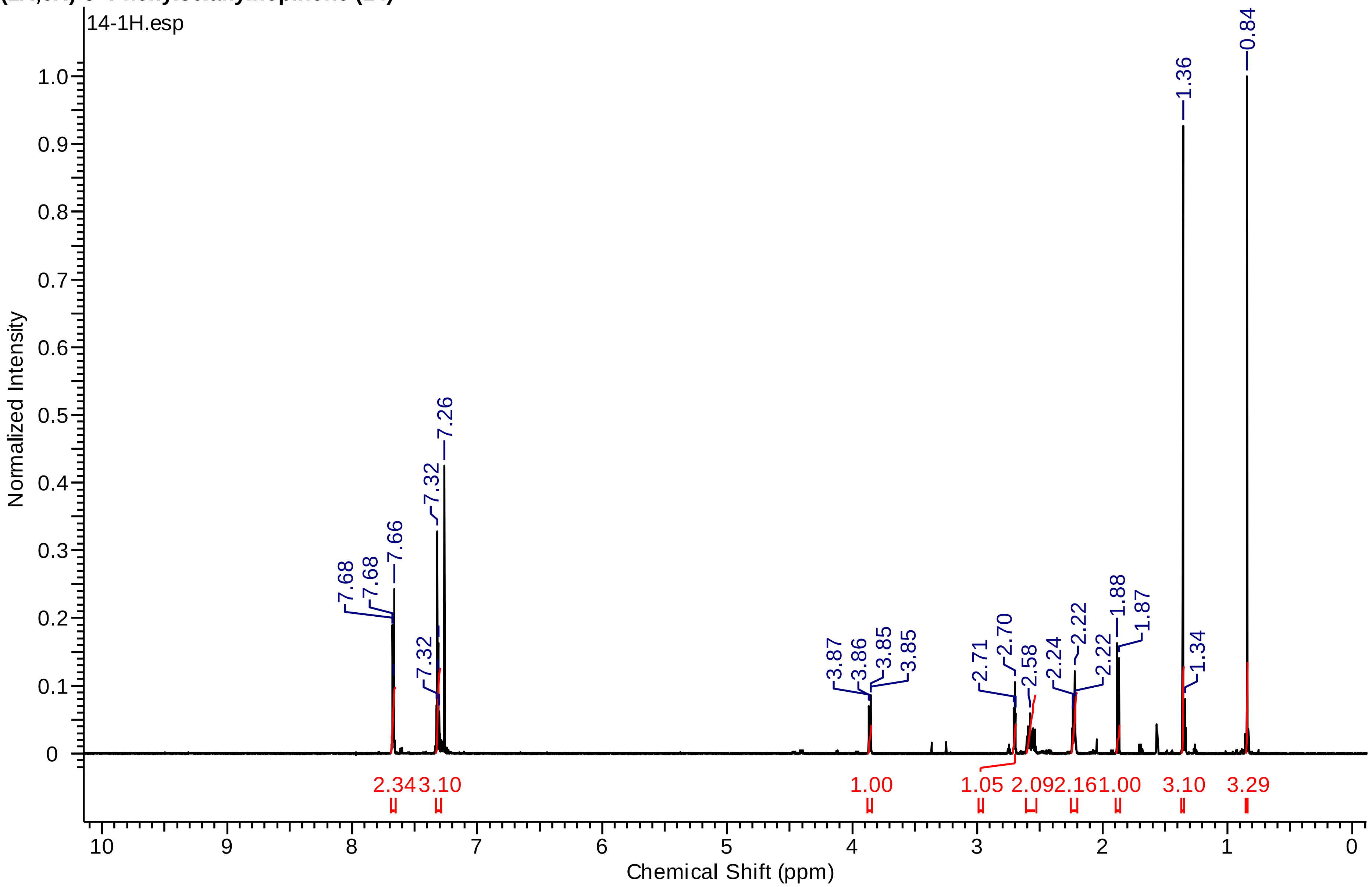

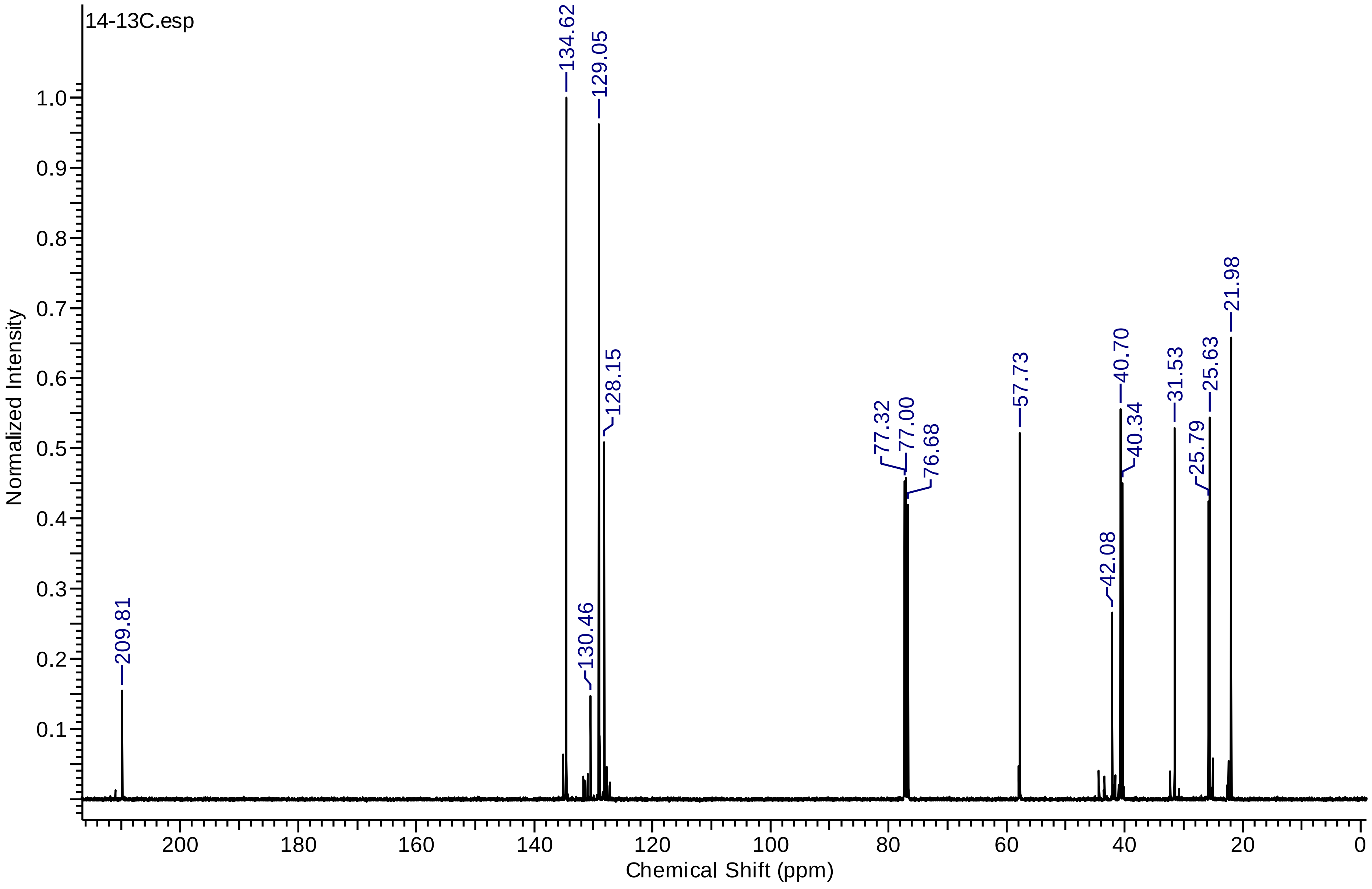

14-13C.esp
209.81
134.62
130.46
129.05
128.15
77.32
77.00
76.68
57.73
42.08
40.70
40.34
31.53
25.79
25.63
21.98
Normalized Intensity
Chemical Shift (ppm)

(1*R*,5*R*)-Apoverbenone (15)

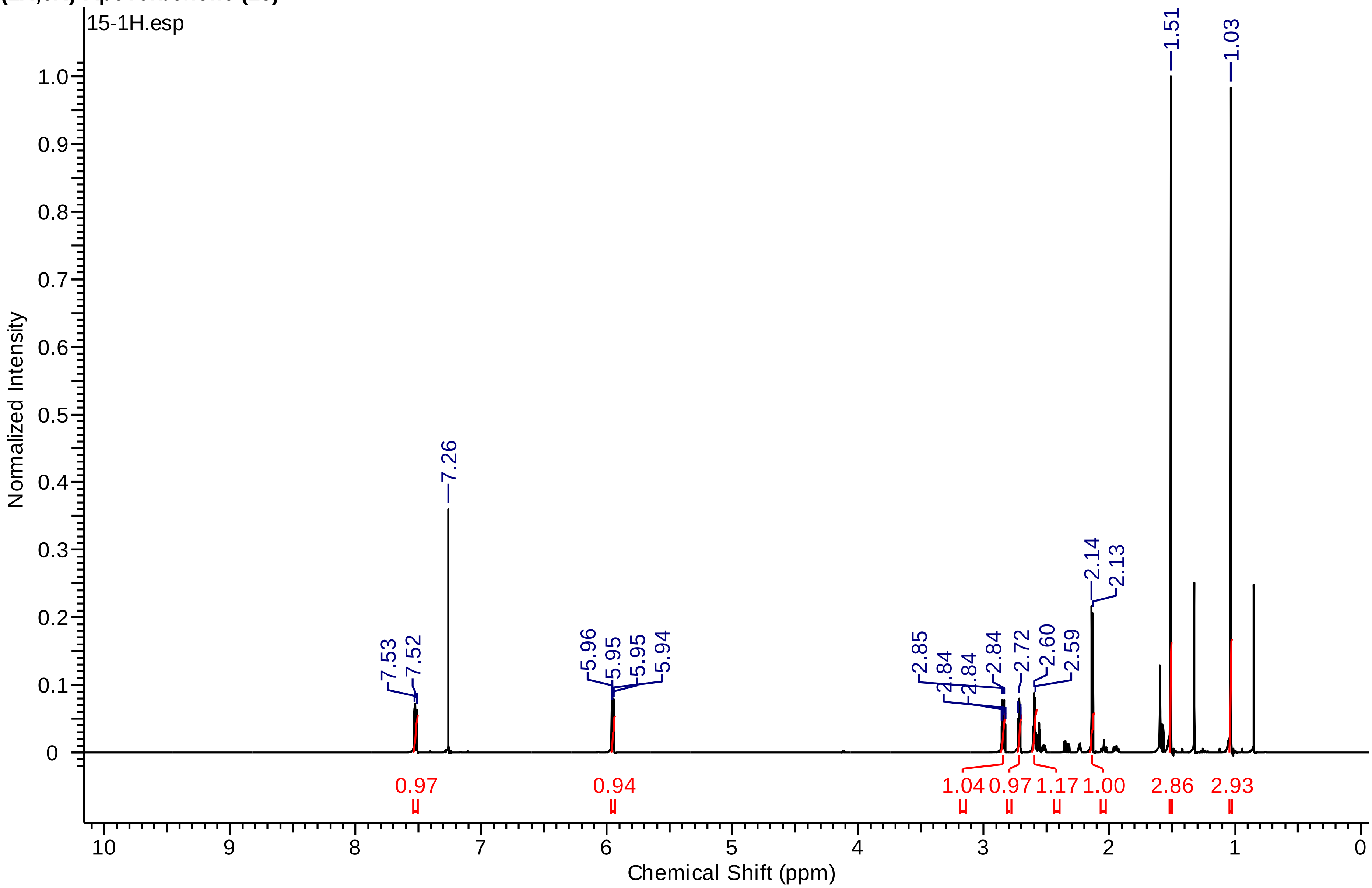

(1*R*,4*R*,5*R*)-(–)-Apopinenol (16)

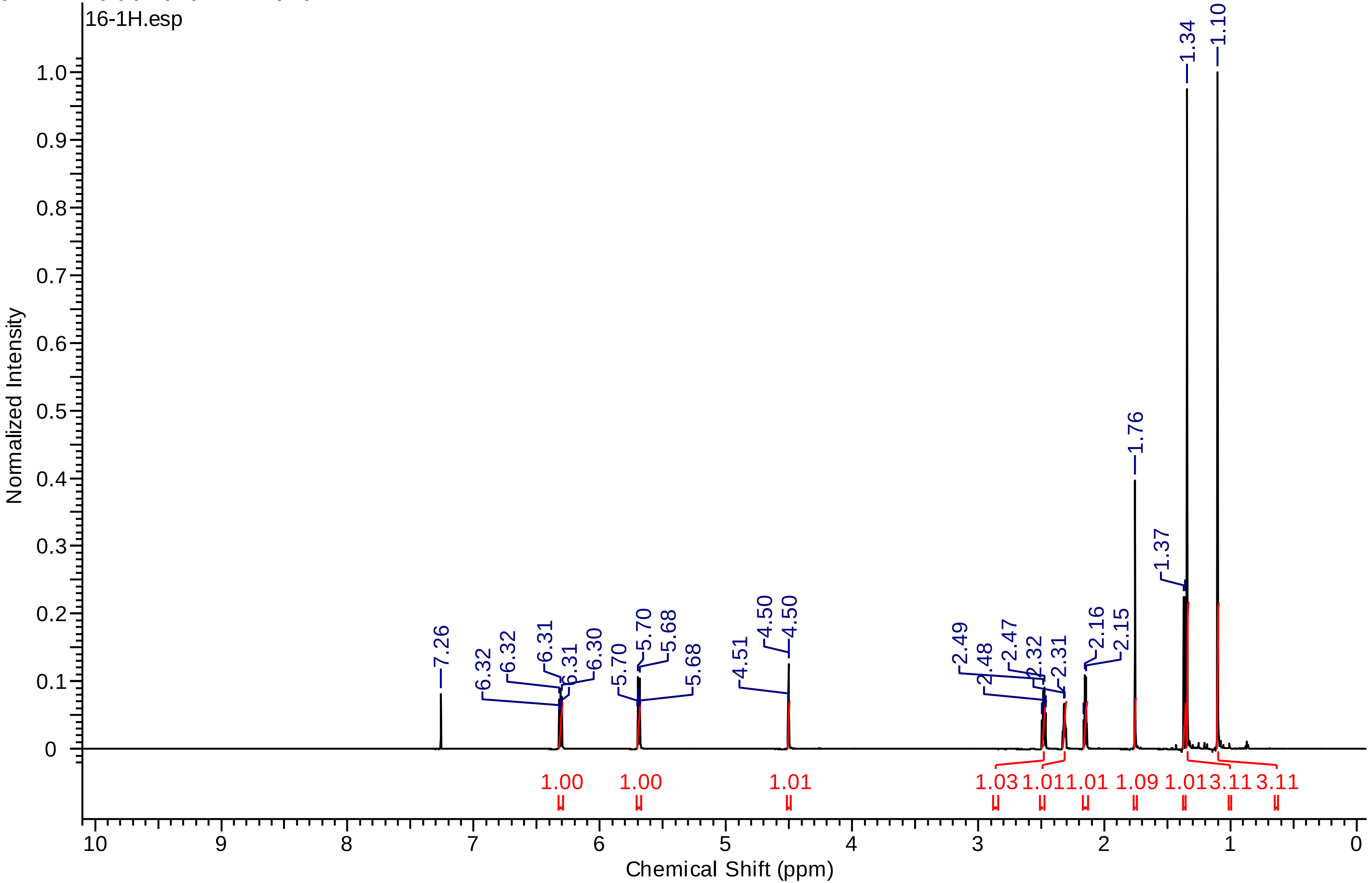

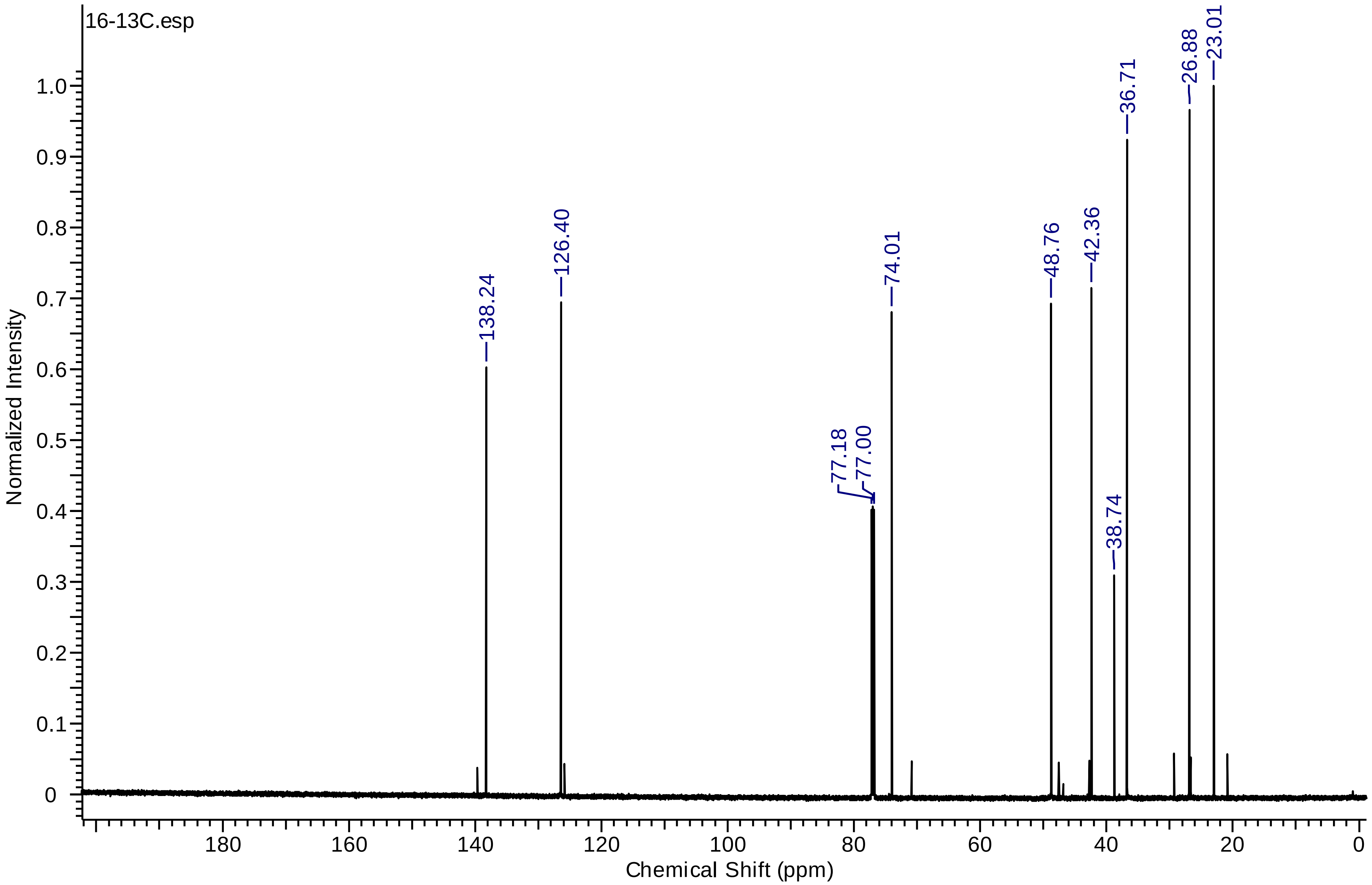
16-13C.esp
138.24
126.40
77.18
77.00
74.01
48.76
42.36
38.74
36.71
26.88
23.01
Normalized Intensity
1.0
0.9
0.8
0.7
0.6
0.5
0.4
0.3
0.2
0.1
0
180
160
140
120
100
80
60
40
20
0
Chemical Shift (ppm)

16 (COSY)

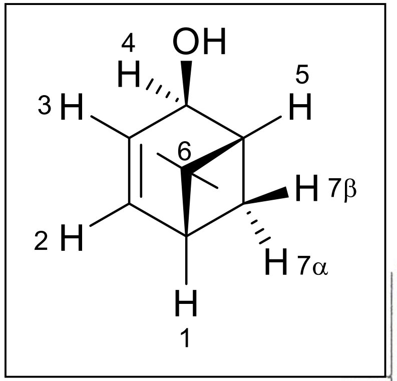

4
OH
H
5
H
3 H
6
H 7β
2 H
H 7α
H
1

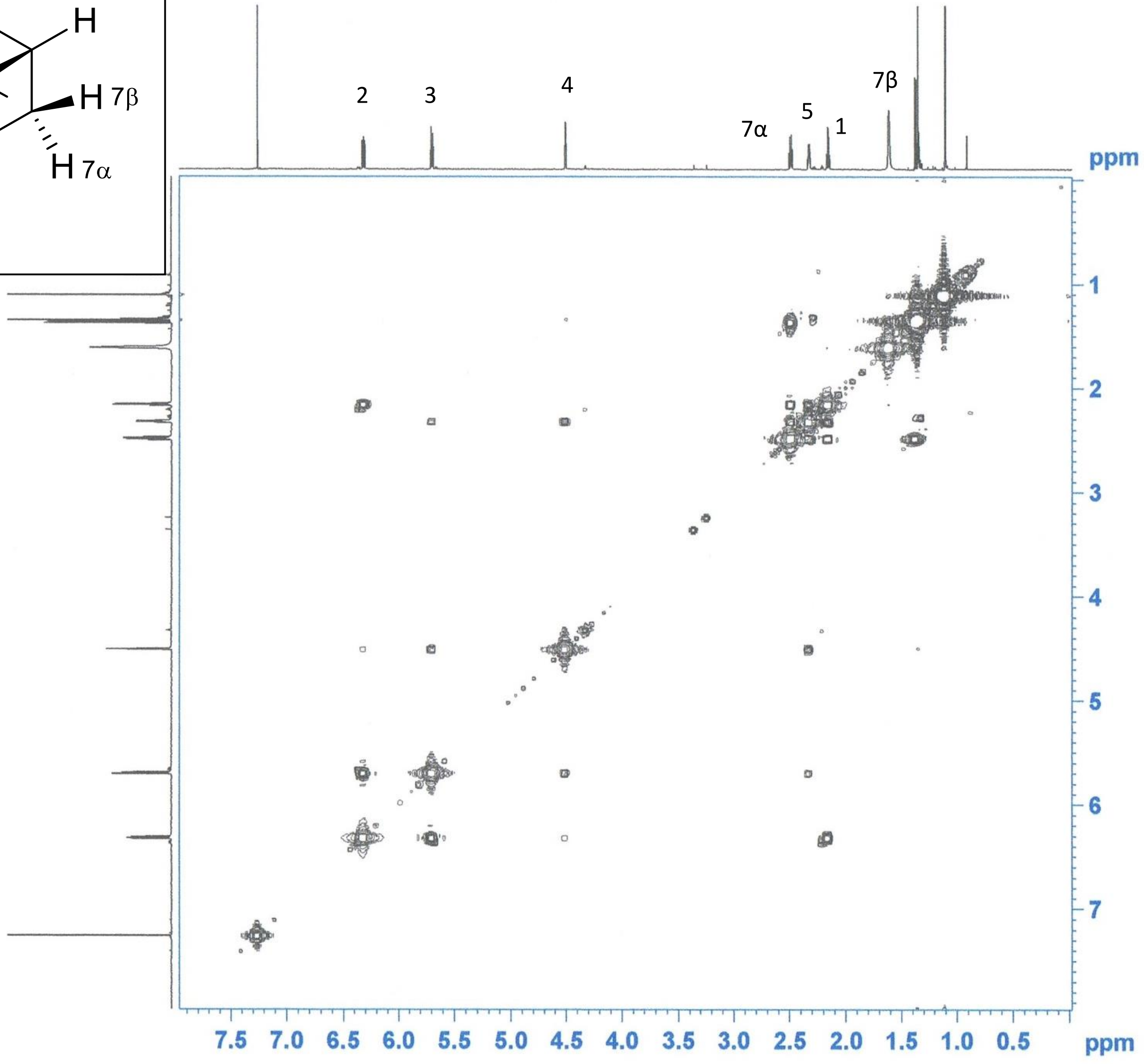

2
3
4
7α
5
1
7β
ppm
1
2
3
4
5
6
7
7.5 7.0 6.5 6.0 5.5 5.0 4.5 4.0 3.5 3.0 2.5 2.0 1.5 1.0 0.5
ppm

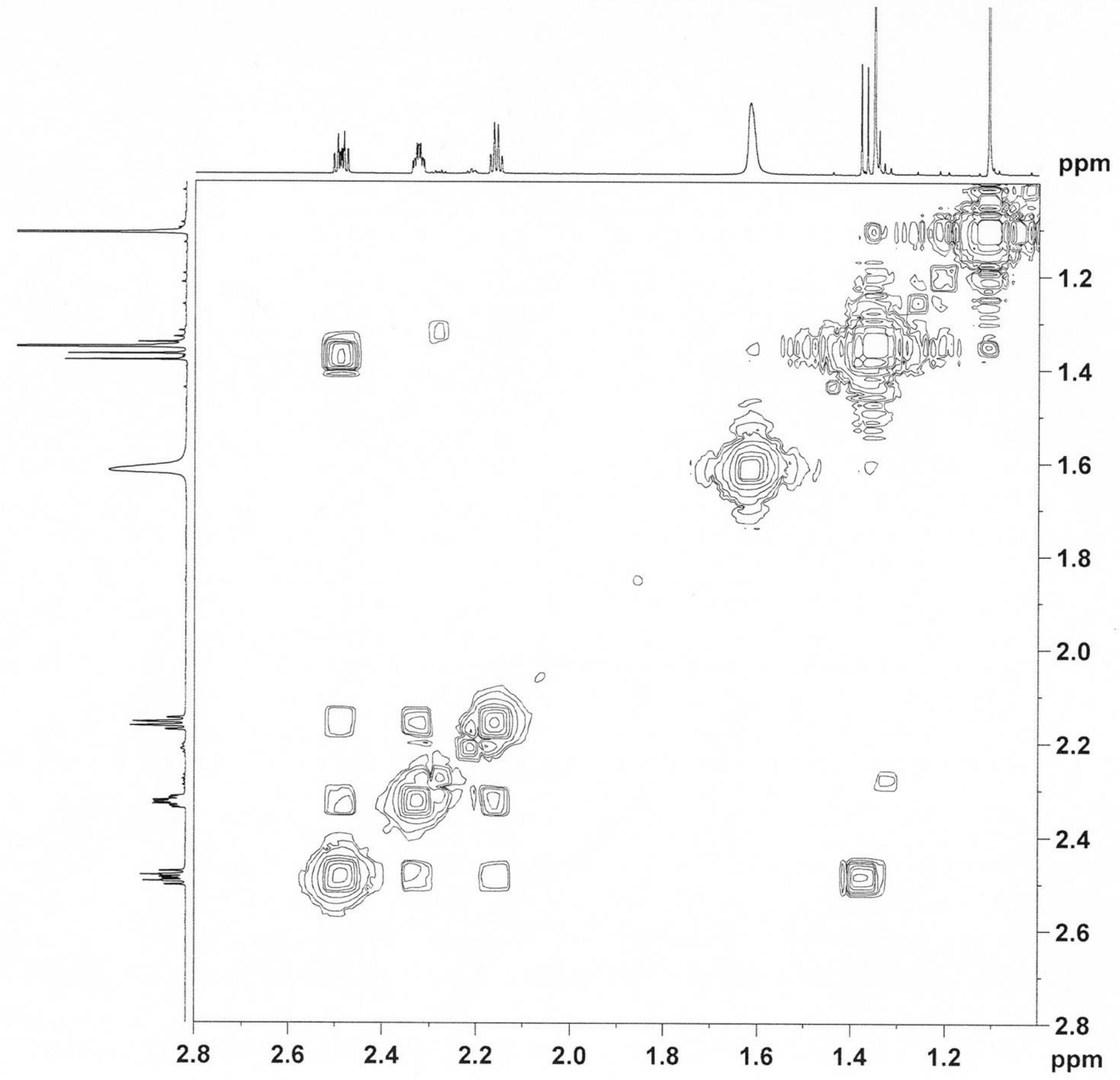
ppm
1.2
1.4
1.6
1.8
2.0
2.2
2.4
2.6
2.8
2.8
2.6
2.4
2.2
2.0
1.8
1.6
1.4
1.2
ppm

16 (NOESY)

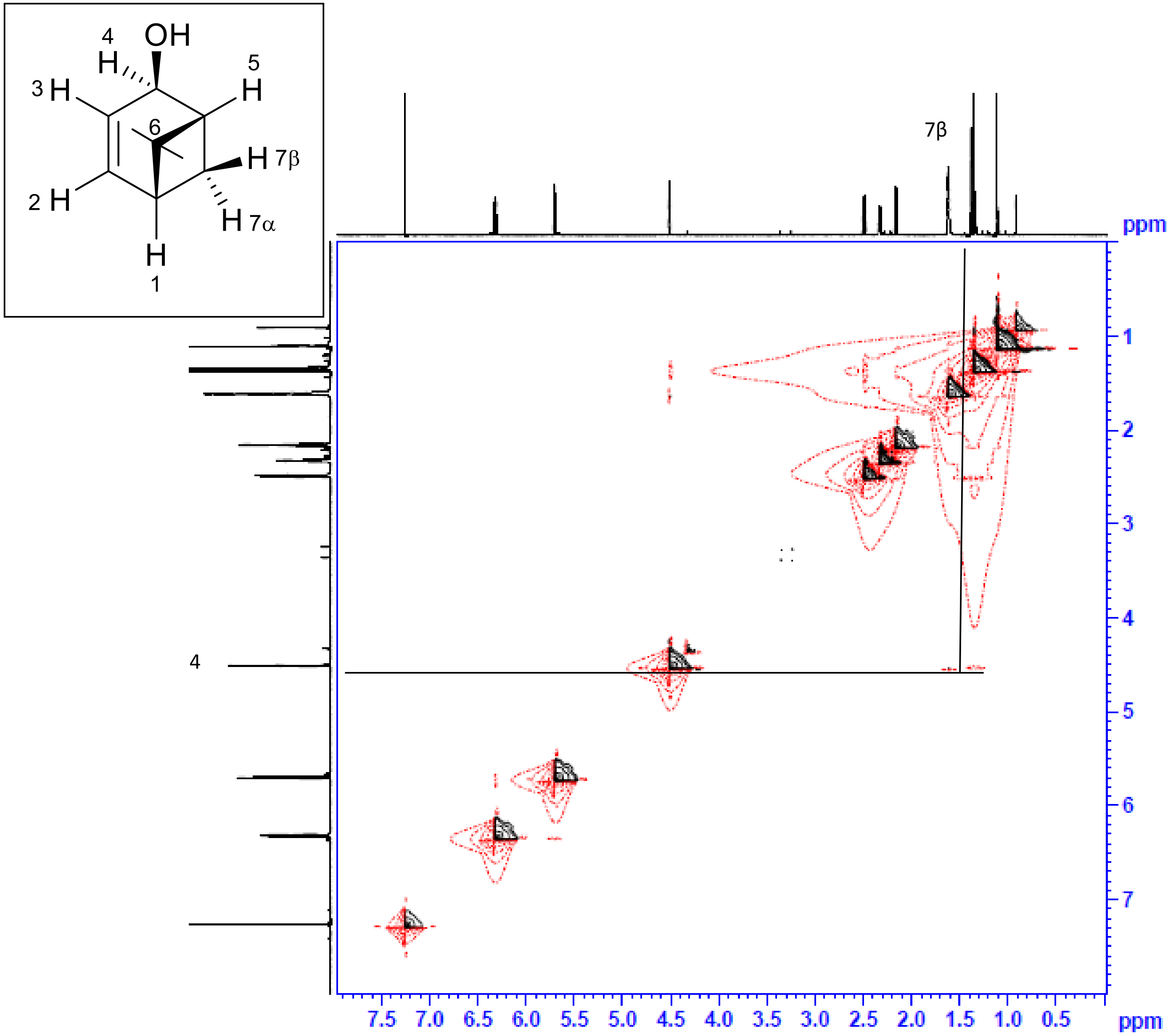

OH
4
H
5
H
3 H
6
H 7β
2 H
H 7α
H
1
7β
ppm
4
1
2
3
4
5
6
7
7.5 7.0 6.5 6.0 5.5 5.0 4.5 4.0 3.5 3.0 2.5 2.0 1.5 1.0 0.5
ppm

16

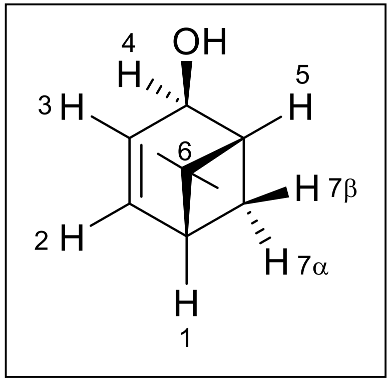
OH
4
H
3 H
5
H
6
H 7β
2 H
H 7α
H
1

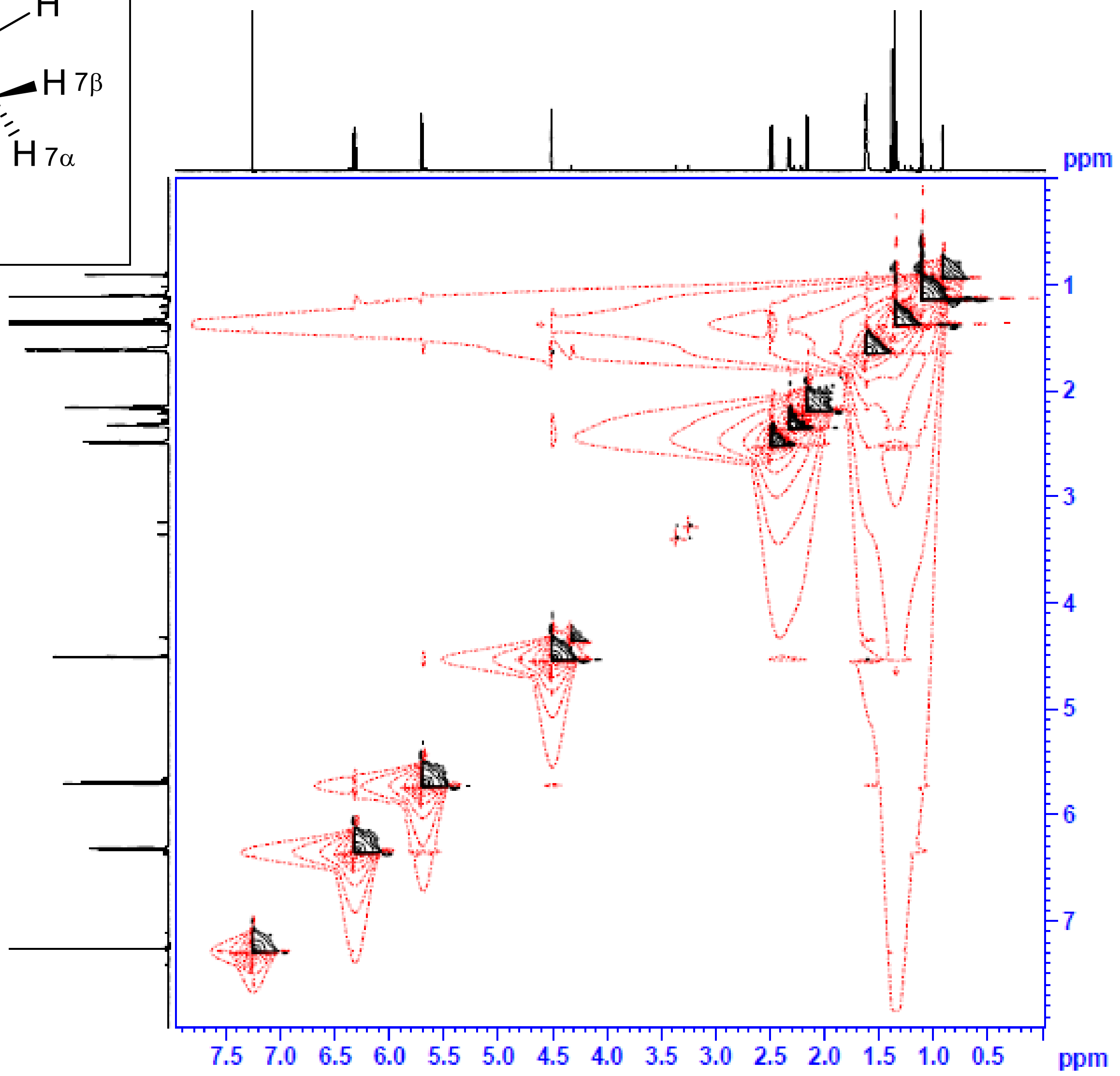
ppm
1
2
3
4
5
6
7
7.5 7.0 6.5 6.0 5.5 5.0 4.5 4.0 3.5 3.0 2.5 2.0 1.5 1.0 0.5
ppm

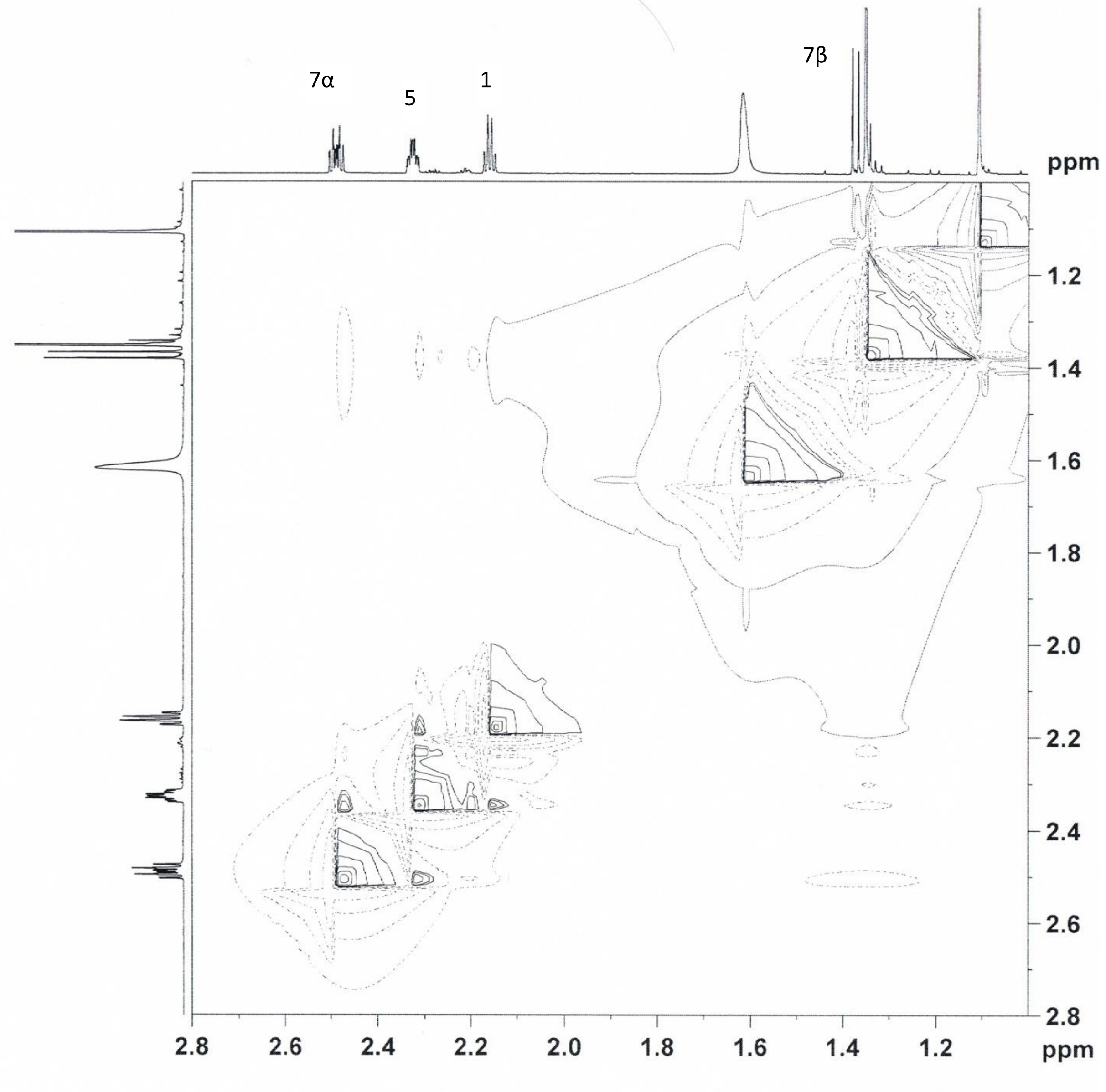
7α
5
1
7β
ppm
1.2
1.4
1.6
1.8
2.0
2.2
2.4
2.6
2.8
2.8
2.6
2.4
2.2
2.0
1.8
1.6
1.4
1.2
ppm

(1*R*,4*S*,5*R*)-(+)-Apopinenol (18)

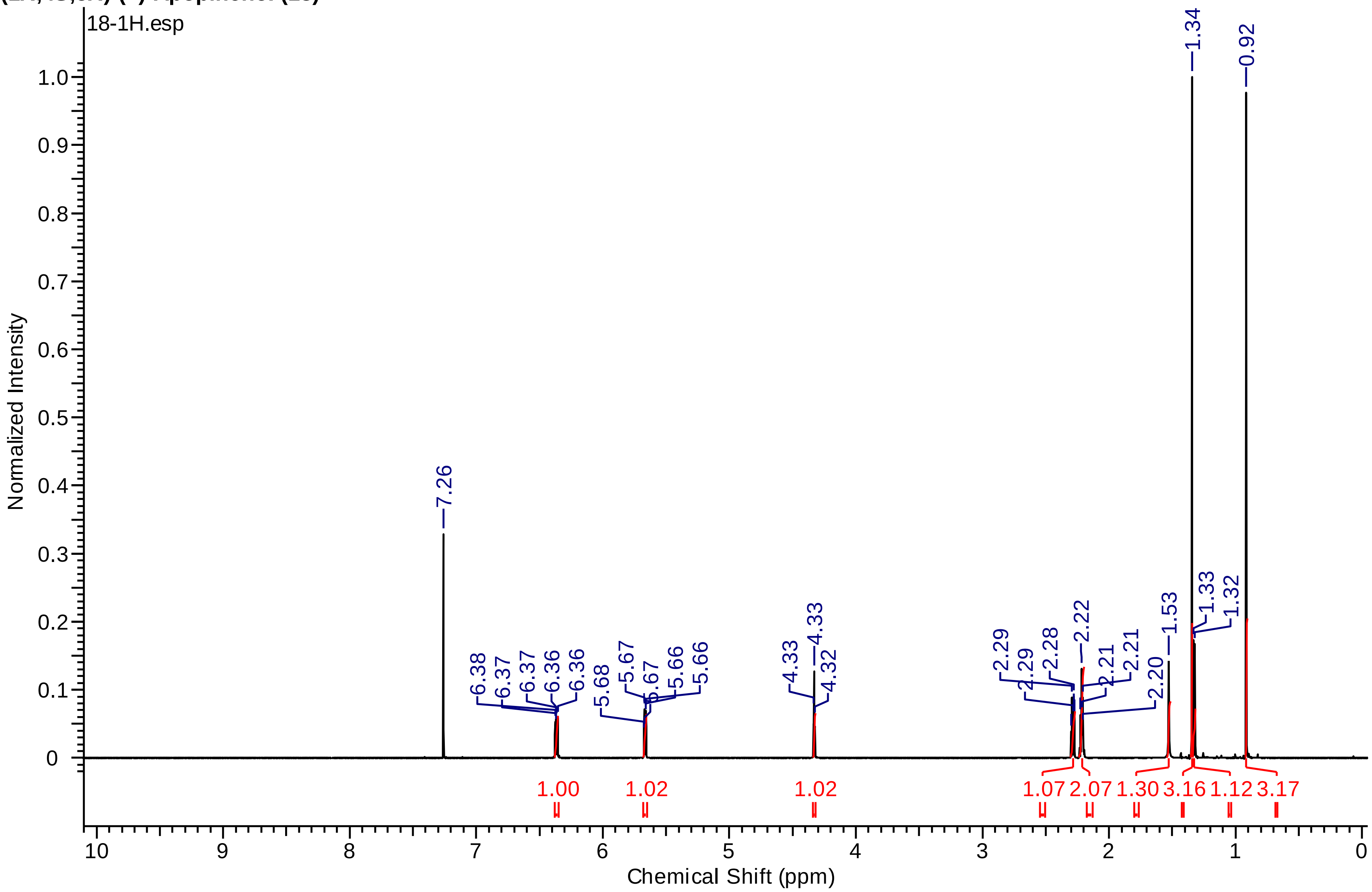

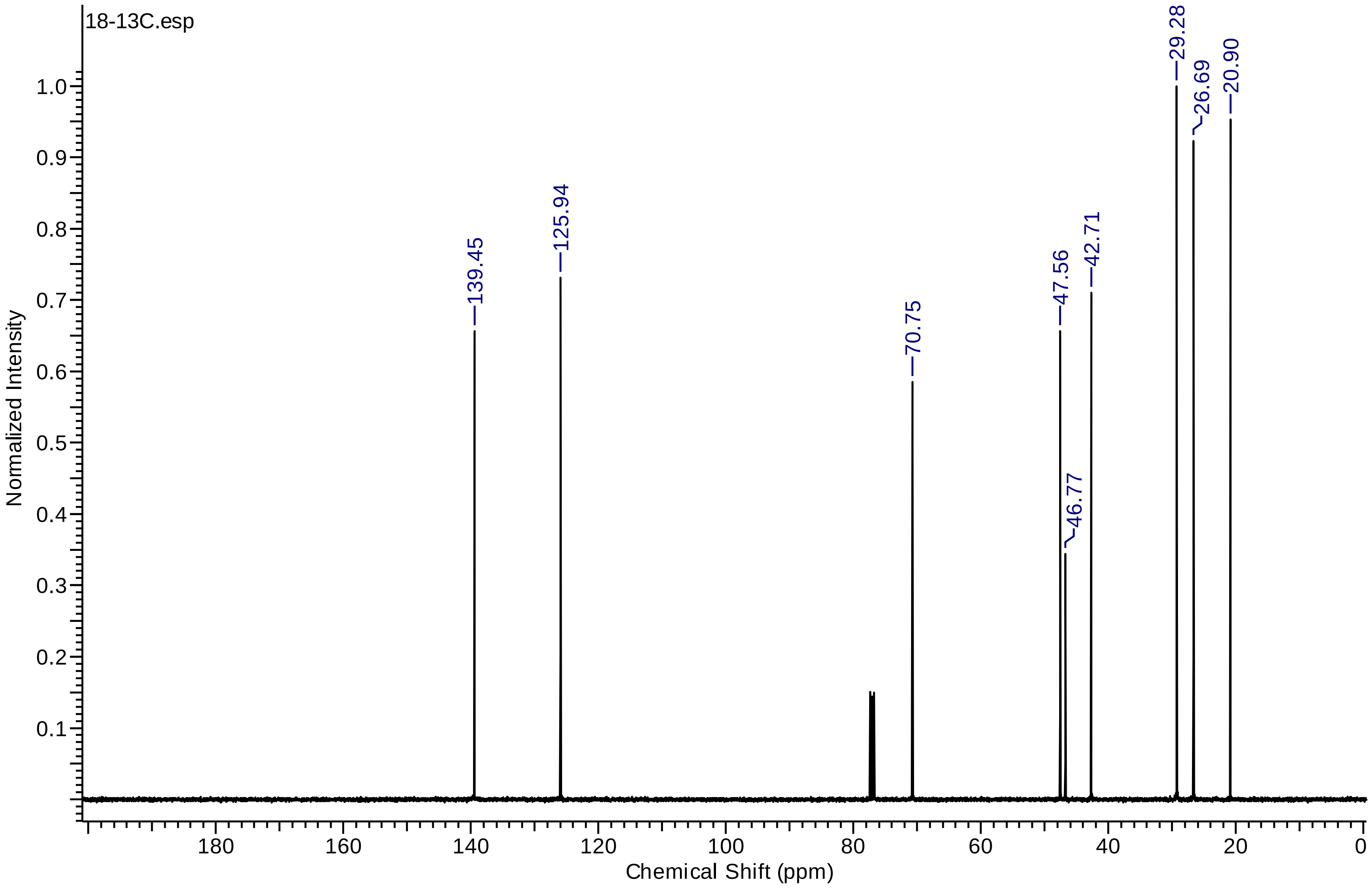
18-13C.esp
139.45
125.94
70.75
47.56
46.77
42.71
29.28
26.69
20.90
Normalized Intensity
1.0
0.9
0.8
0.7
0.6
0.5
0.4
0.3
0.2
0.1
180
160
140
120
100
80
60
40
20
0
Chemical Shift (ppm)

18 (COSY)

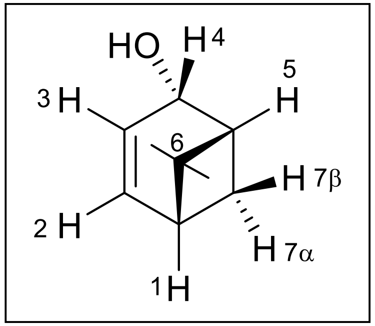
HO
H 4
5
3 H
H
6
H 7β
2 H
H 7α
1 H

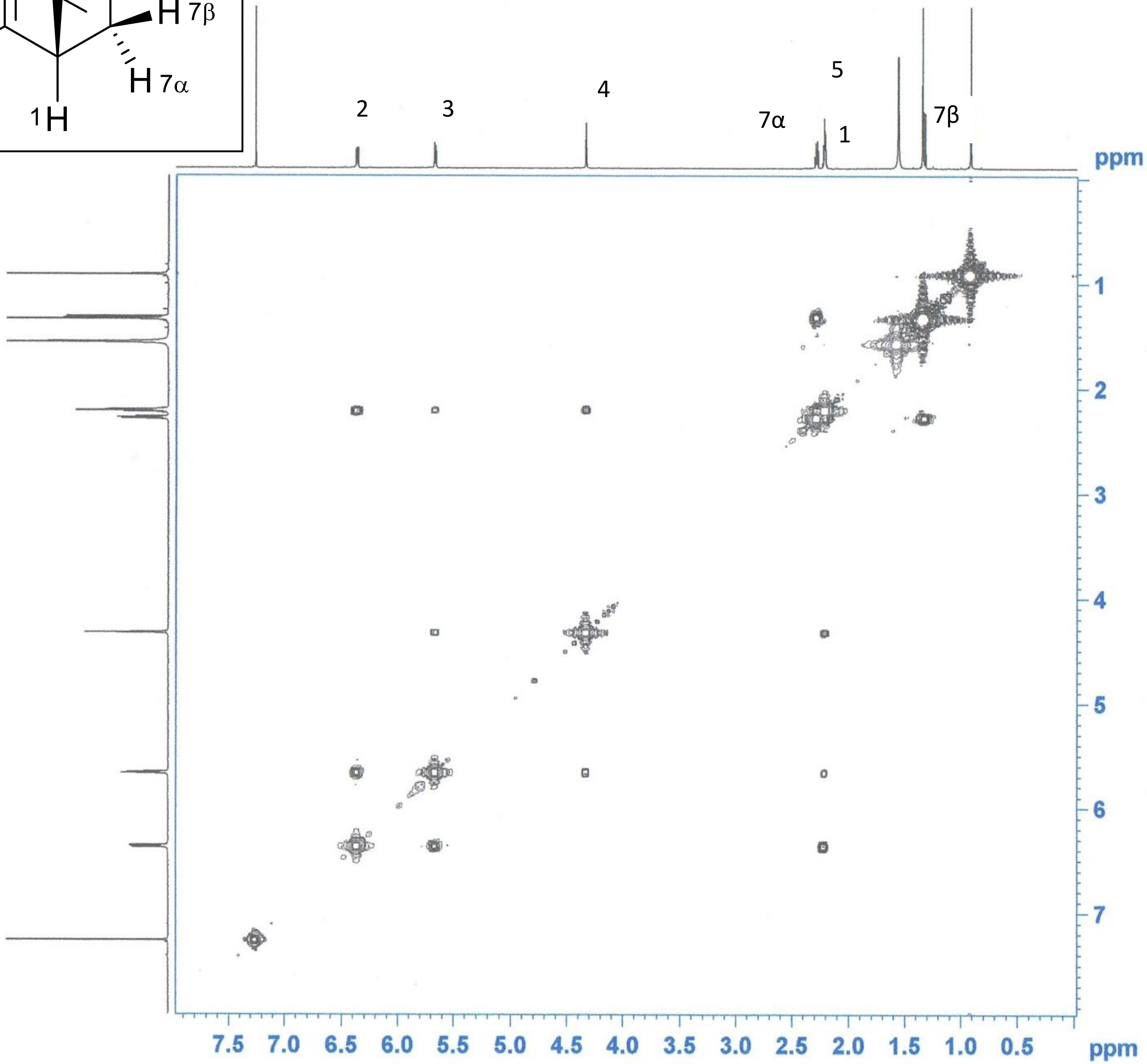
2
3
4
5
7α
1
7β
ppm
7.5 7.0 6.5 6.0 5.5 5.0 4.5 4.0 3.5 3.0 2.5 2.0 1.5 1.0 0.5
ppm
1
2
3
4
5
6
7

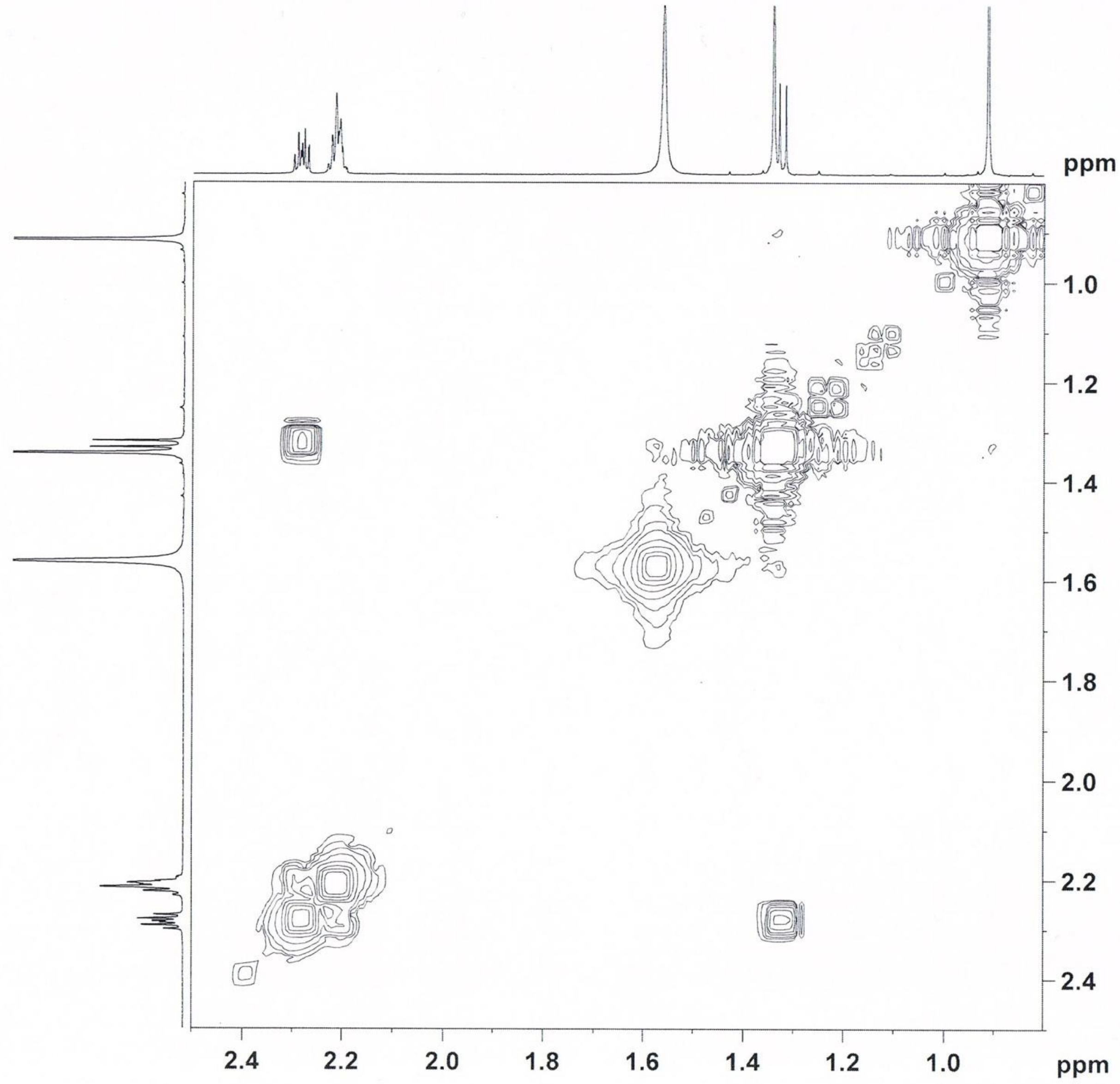
ppm
1.0
1.2
1.4
1.6
1.8
2.0
2.2
2.4
2.4
2.2
2.0
1.8
1.6
1.4
1.2
1.0
ppm

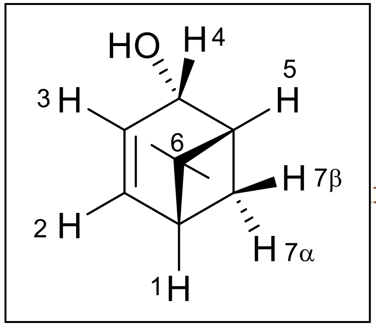
HO
H4
5
H
3 H
6
H 7β
2 H
H 7α
1H

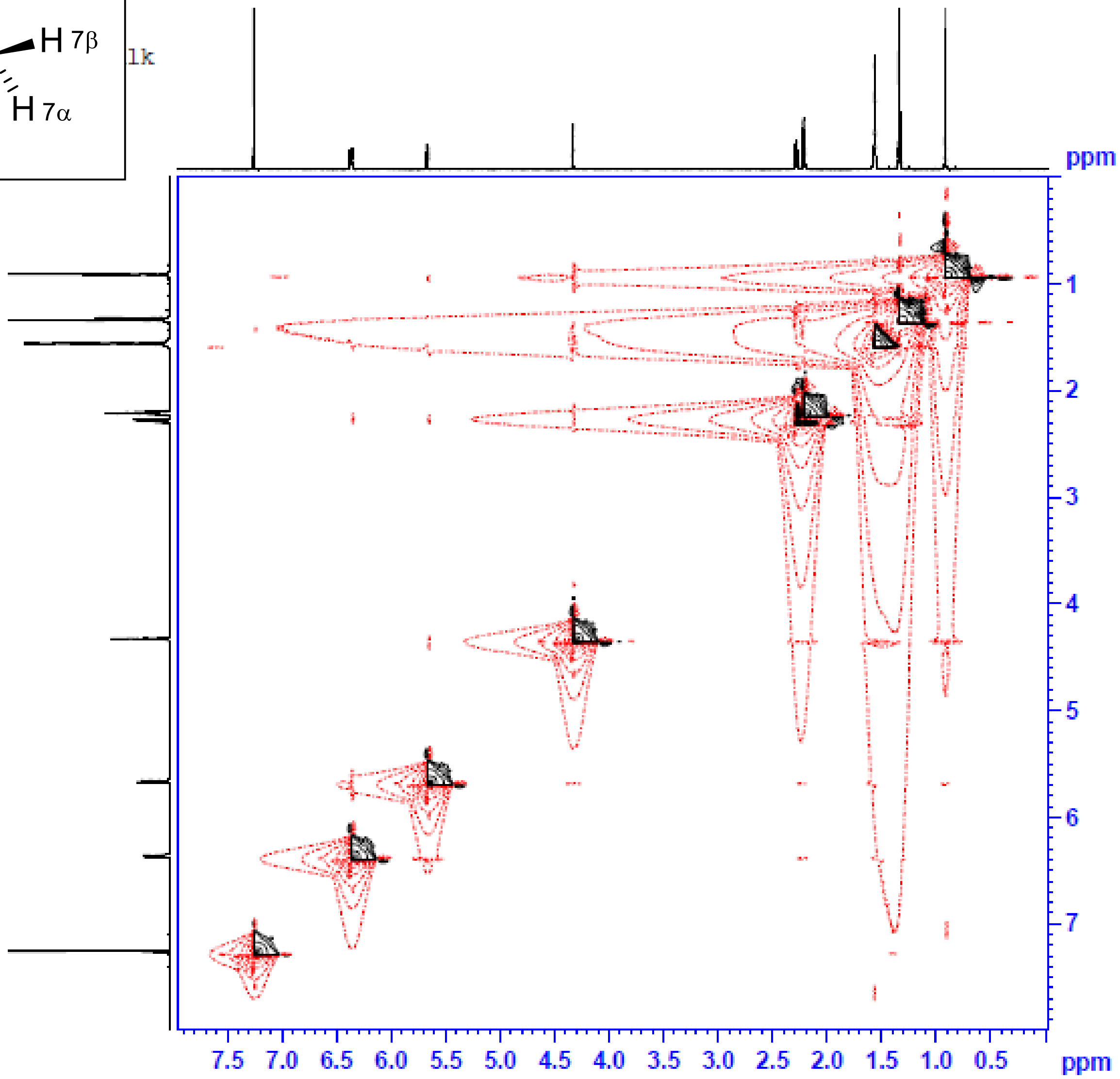
1k
ppm
1
2
3
4
5
6
7
7.5 7.0 6.5 6.0 5.5 5.0 4.5 4.0 3.5 3.0 2.5 2.0 1.5 1.0 0.5
ppm

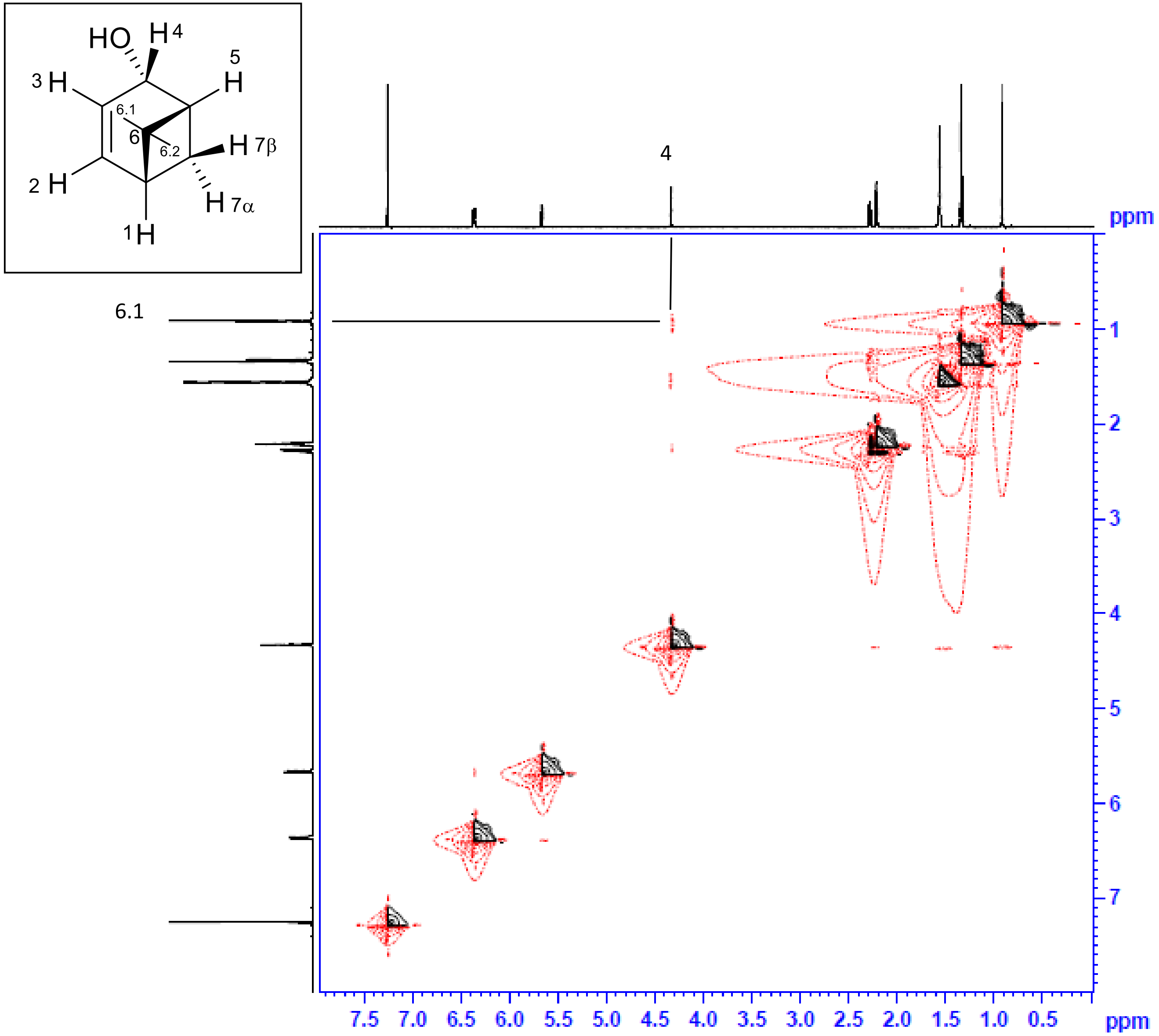
HO
H 4
5
3 H
H
6.1
6
6.2
H 7β
2 H
H 7α
1 H
4
6.1
ppm
7.5 7.0 6.5 6.0 5.5 5.0 4.5 4.0 3.5 3.0 2.5 2.0 1.5 1.0 0.5
1 2 3 4 5 6 7

Diphenyl (((1*R*,2*R*,5*S*)-δ-pinen-3-yl)methyl)phosphine oxide (21)

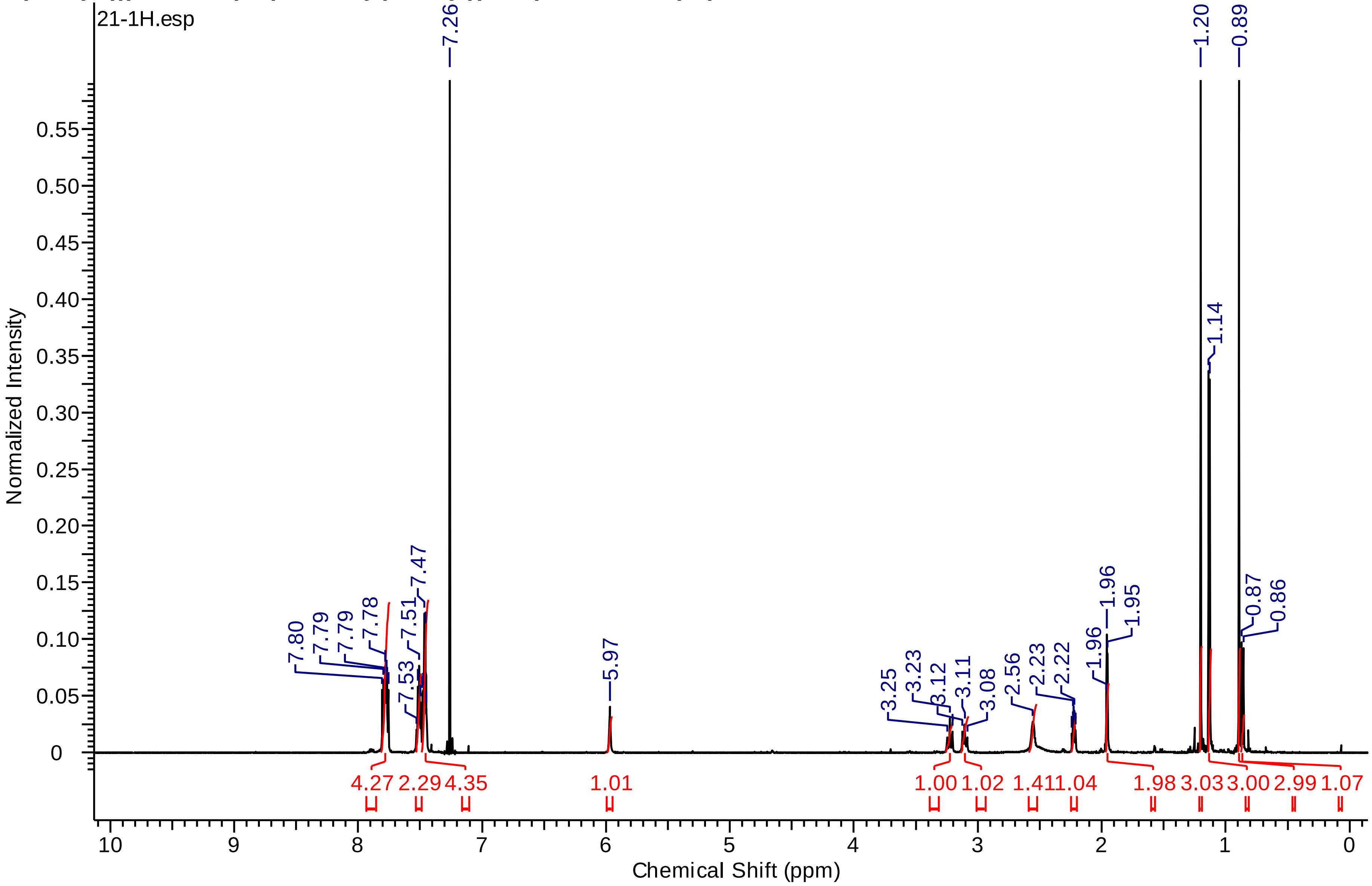

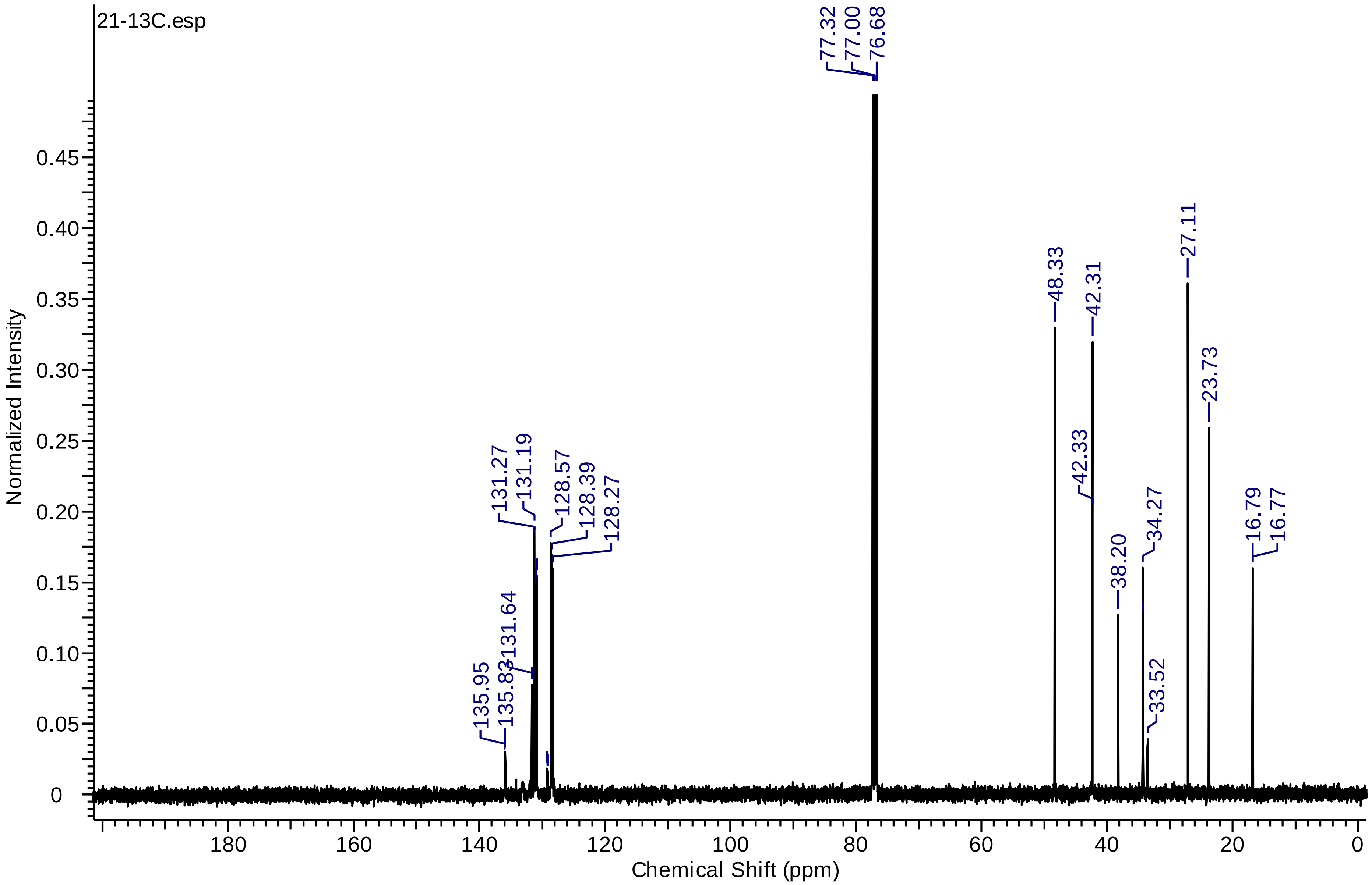

21-13C.esp
77.32
77.00
76.68
48.33
42.31
27.11
23.73
42.33
131.27
131.19
128.57
128.39
128.27
34.27
16.79
16.77
38.20
131.64
135.95
135.83
33.52
Normalized Intensity
0.45
0.40
0.35
0.30
0.25
0.20
0.15
0.10
0.05
0
180
160
140
120
100
80
60
40
20
0
Chemical Shift (ppm)

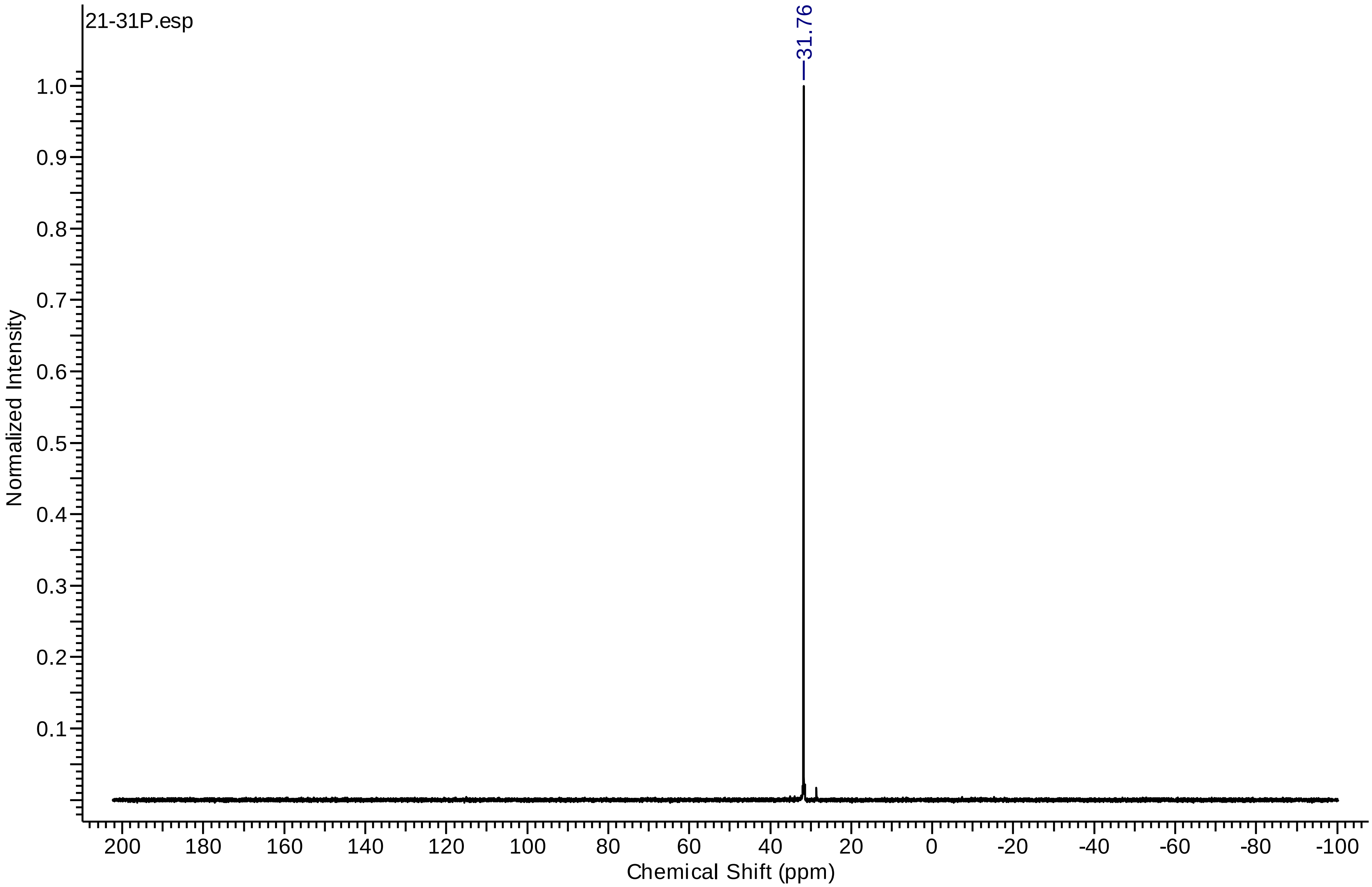
21-31P.esp
31.76
Normalized Intensity
1.0
0.9
0.8
0.7
0.6
0.5
0.4
0.3
0.2
0.1
200 180 160 140 120 100 80 60 40 20 0 -20 -40 -60 -80 -100
Chemical Shift (ppm)

(((1*R*,2*R*,3*R*,4*R*,5*R*)-4-Hydroxypinan-3-yl)methyl)diphenylphosphine oxide (22)

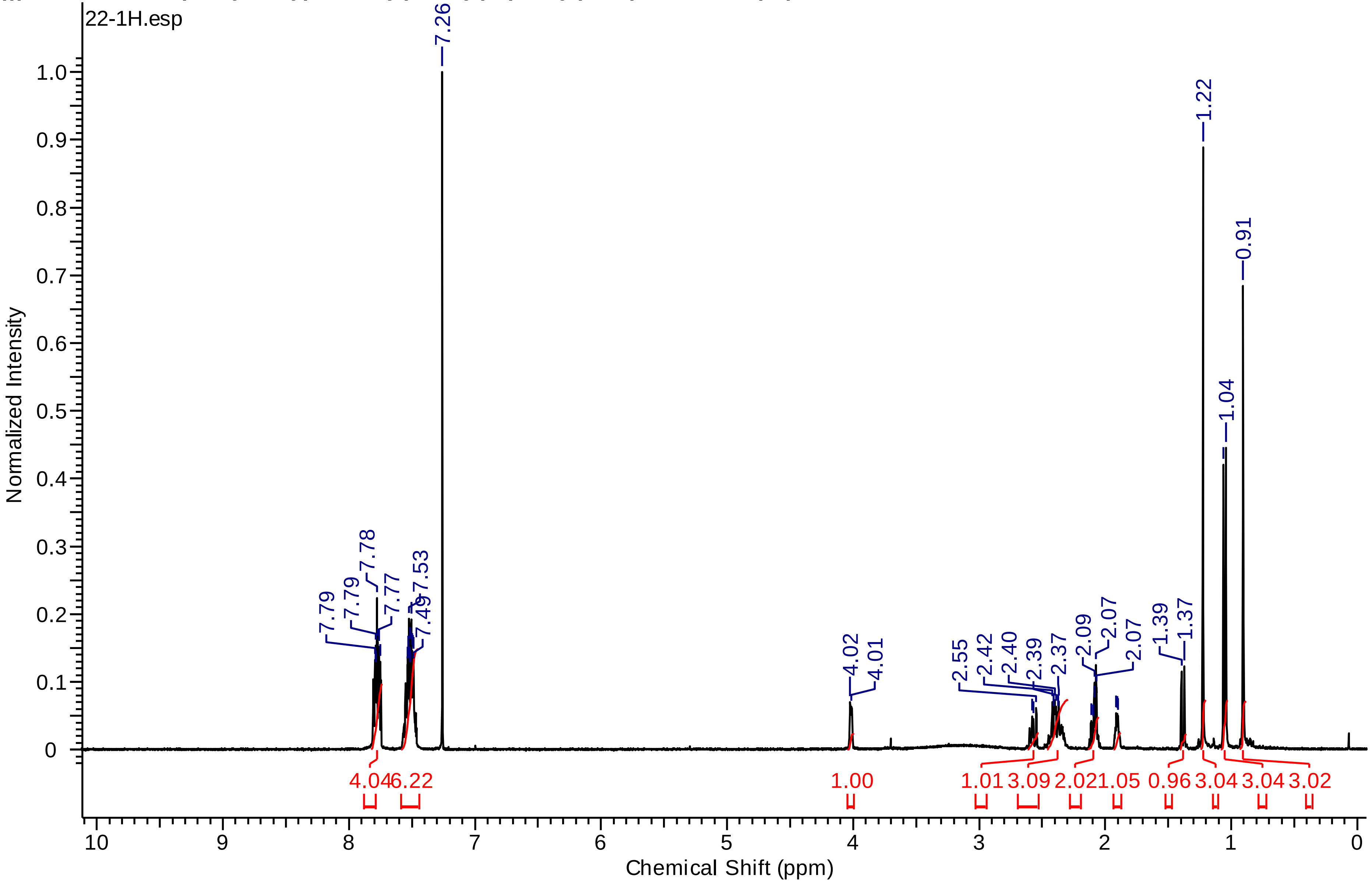

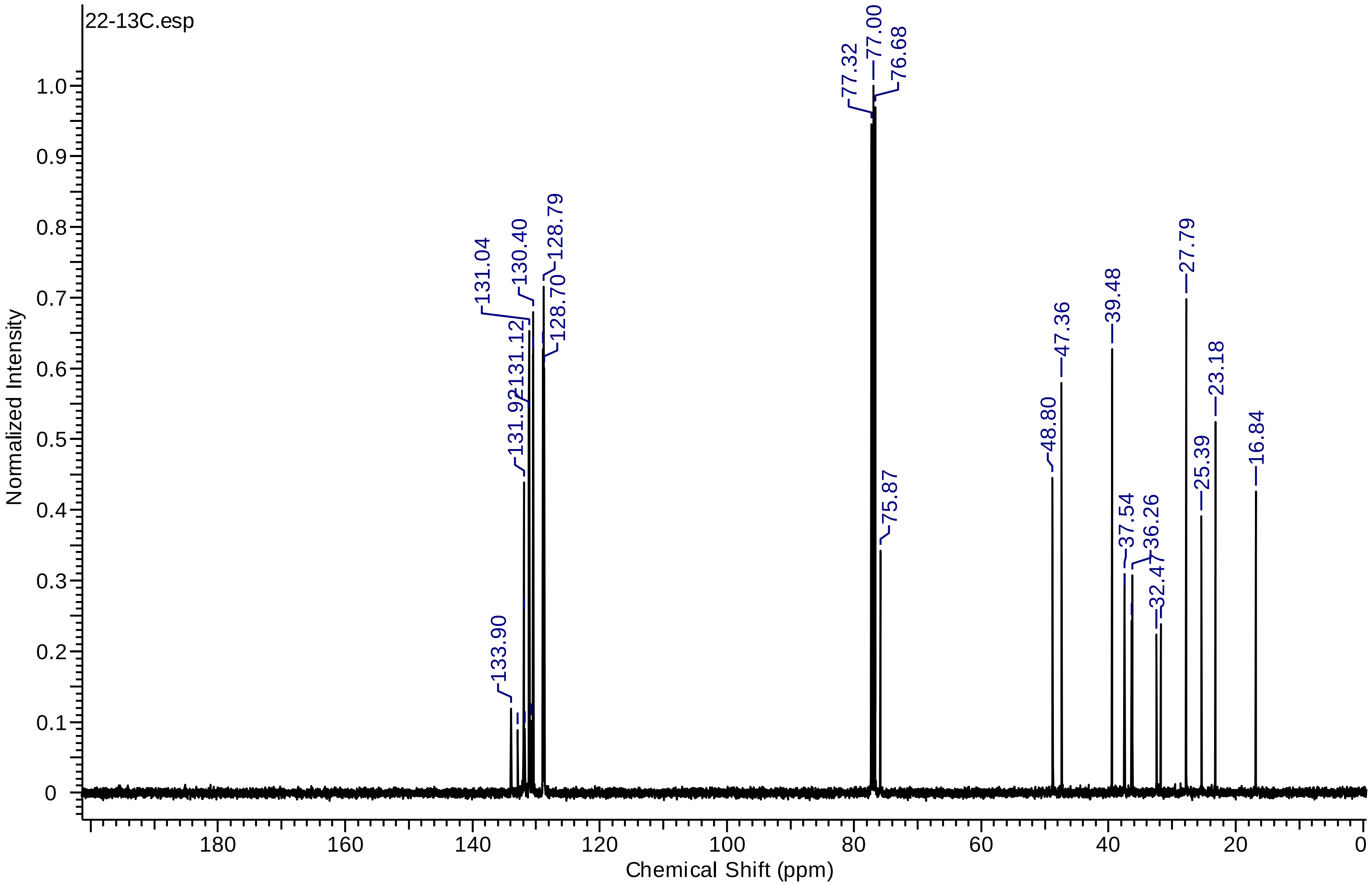
22-13C.esp
Normalized Intensity
Chemical Shift (ppm)
133.90
131.92
131.12
131.04
130.40
128.79
128.70
77.32
77.00
76.68
75.87
48.80
47.36
39.48
37.54
36.26
32.47
27.79
25.39
23.18
16.84

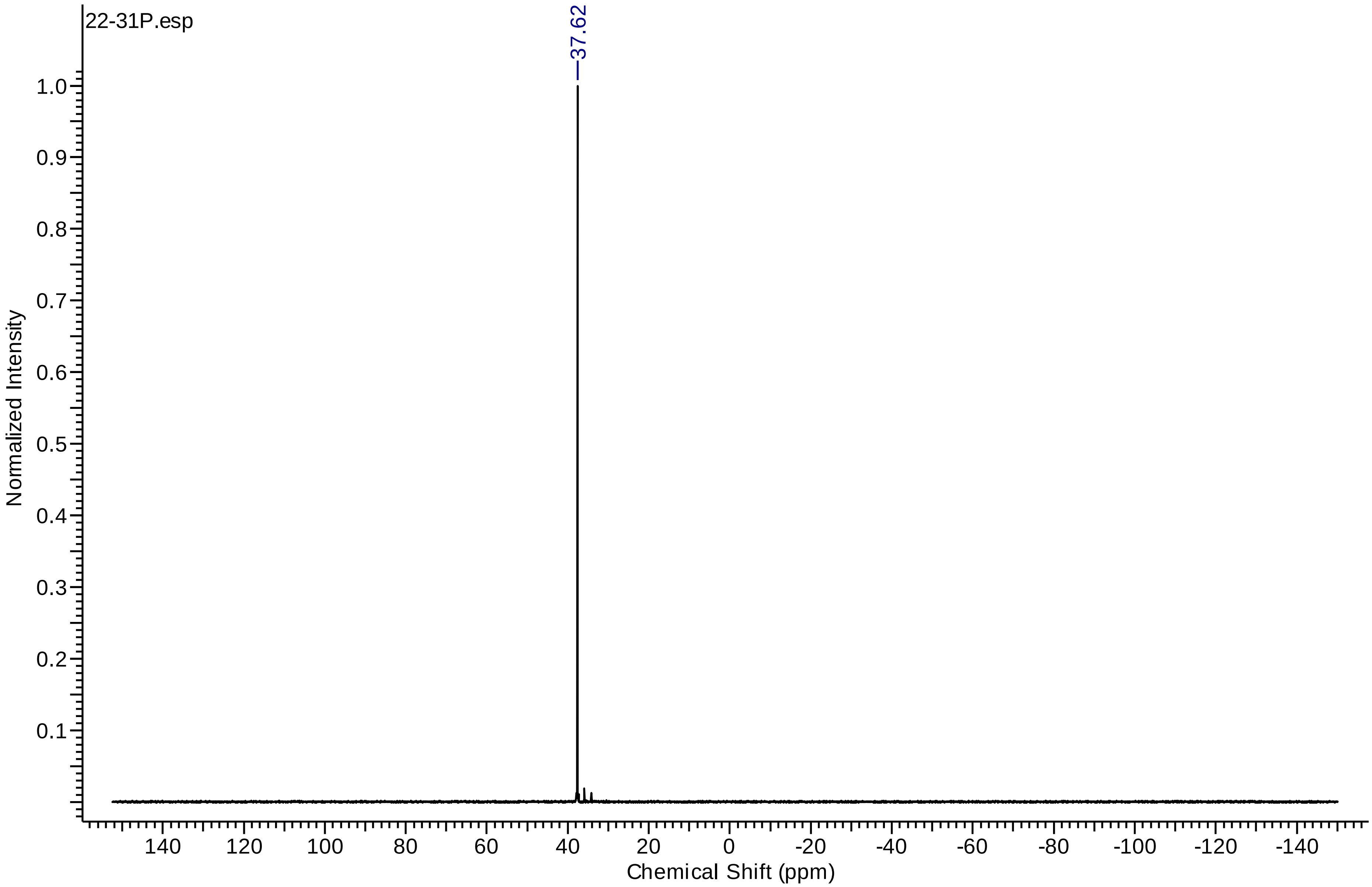

22-31P.esp
37.62
1.0
0.9
0.8
0.7
0.6
0.5
0.4
0.3
0.2
0.1
Normalized Intensity
140
120
100
80
60
40
20
0
-20
-40
-60
-80
-100
-120
-140
Chemical Shift (ppm)

(1*R*,2*R*,3*R*,4*R*,5*R*)-3-((Diphenylphosphanyl)methyl)isoverbanol (23)

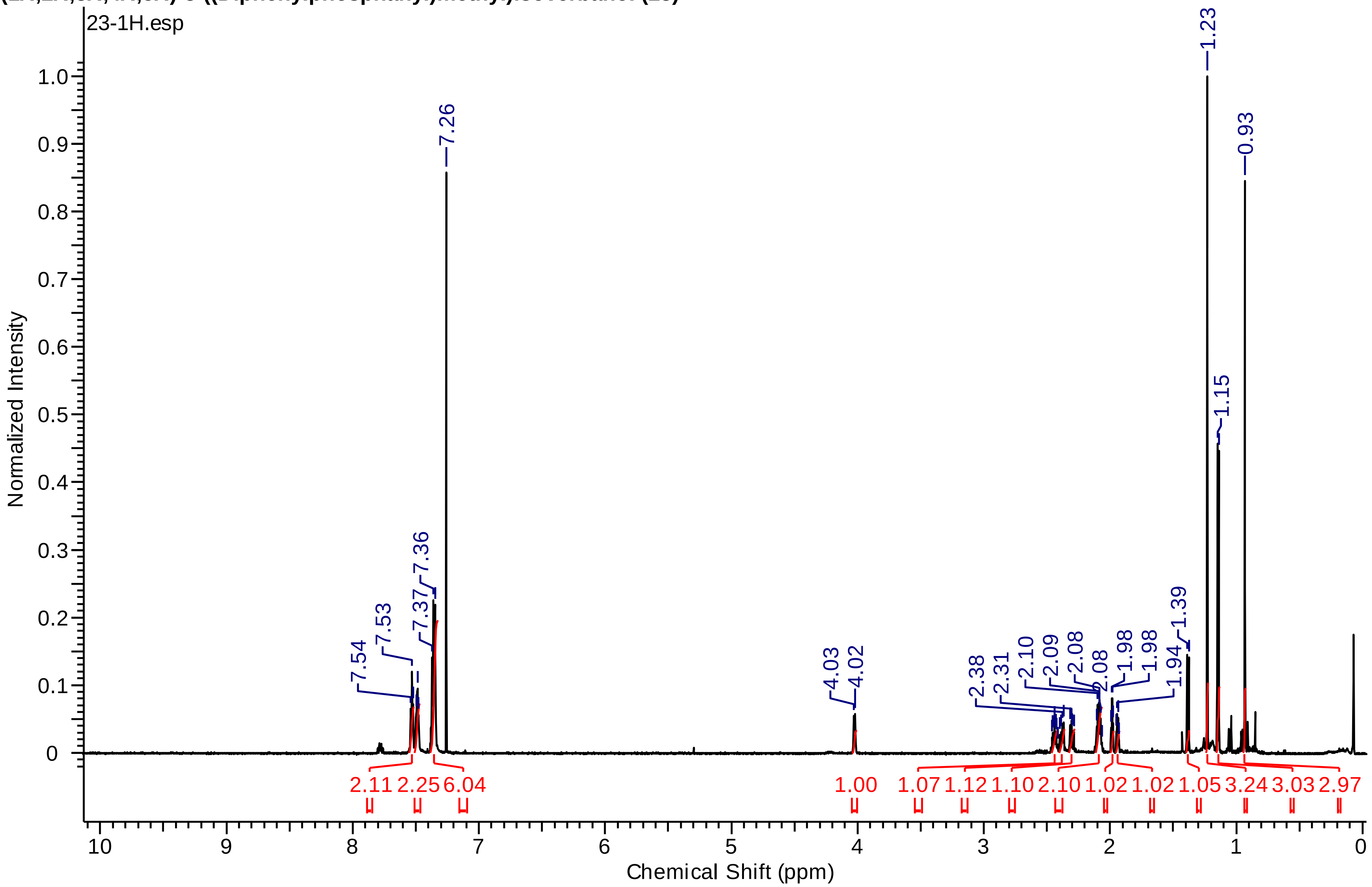

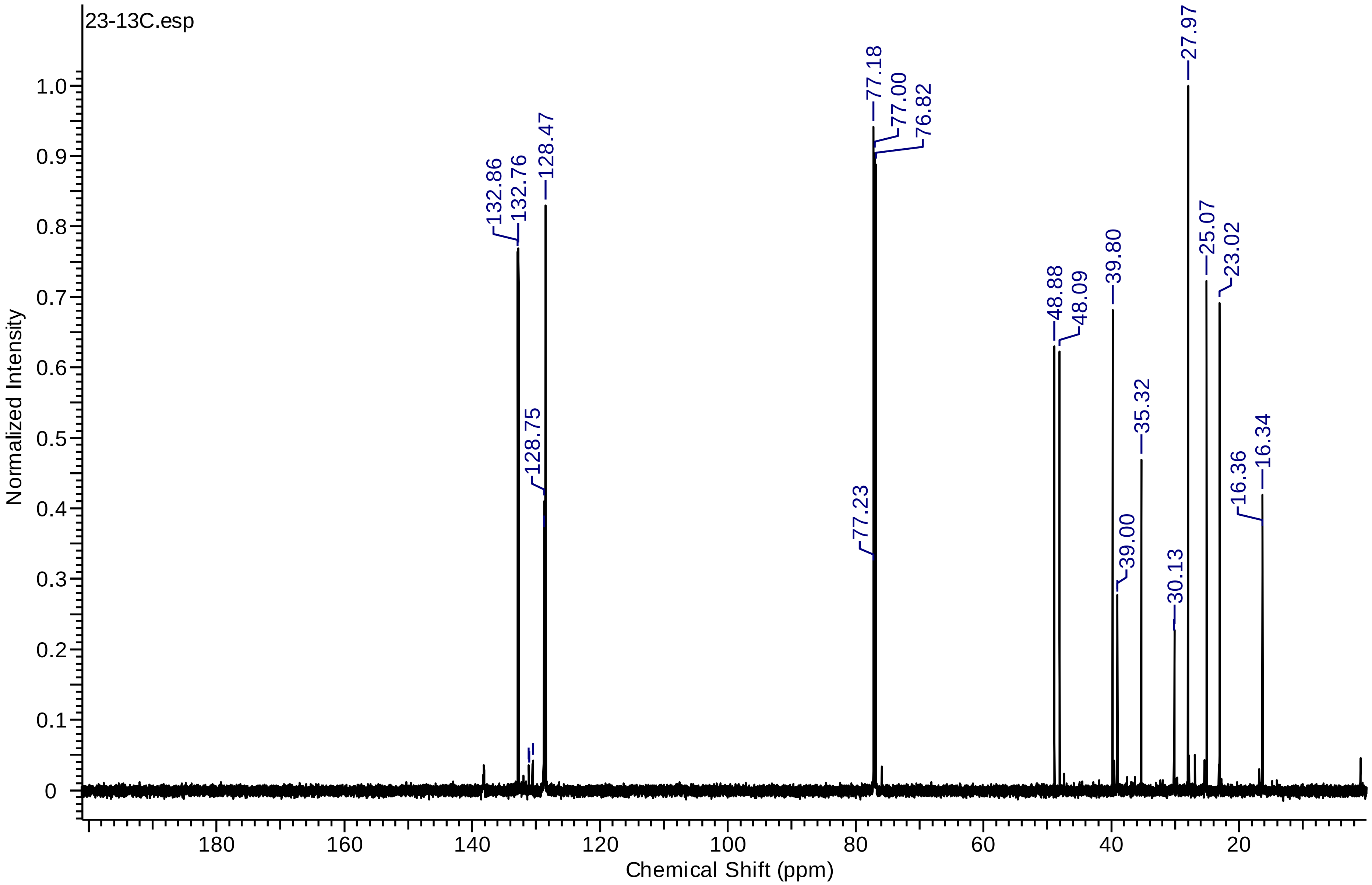
23-13C.esp
132.86
132.76
128.47
128.75
77.18
77.00
76.82
77.23
48.88
48.09
39.80
39.00
35.32
30.13
27.97
25.07
23.02
16.36
16.34
Normalized Intensity
Chemical Shift (ppm)
1.0
0.9
0.8
0.7
0.6
0.5
0.4
0.3
0.2
0.1
0
180
160
140
120
100
80
60
40
20

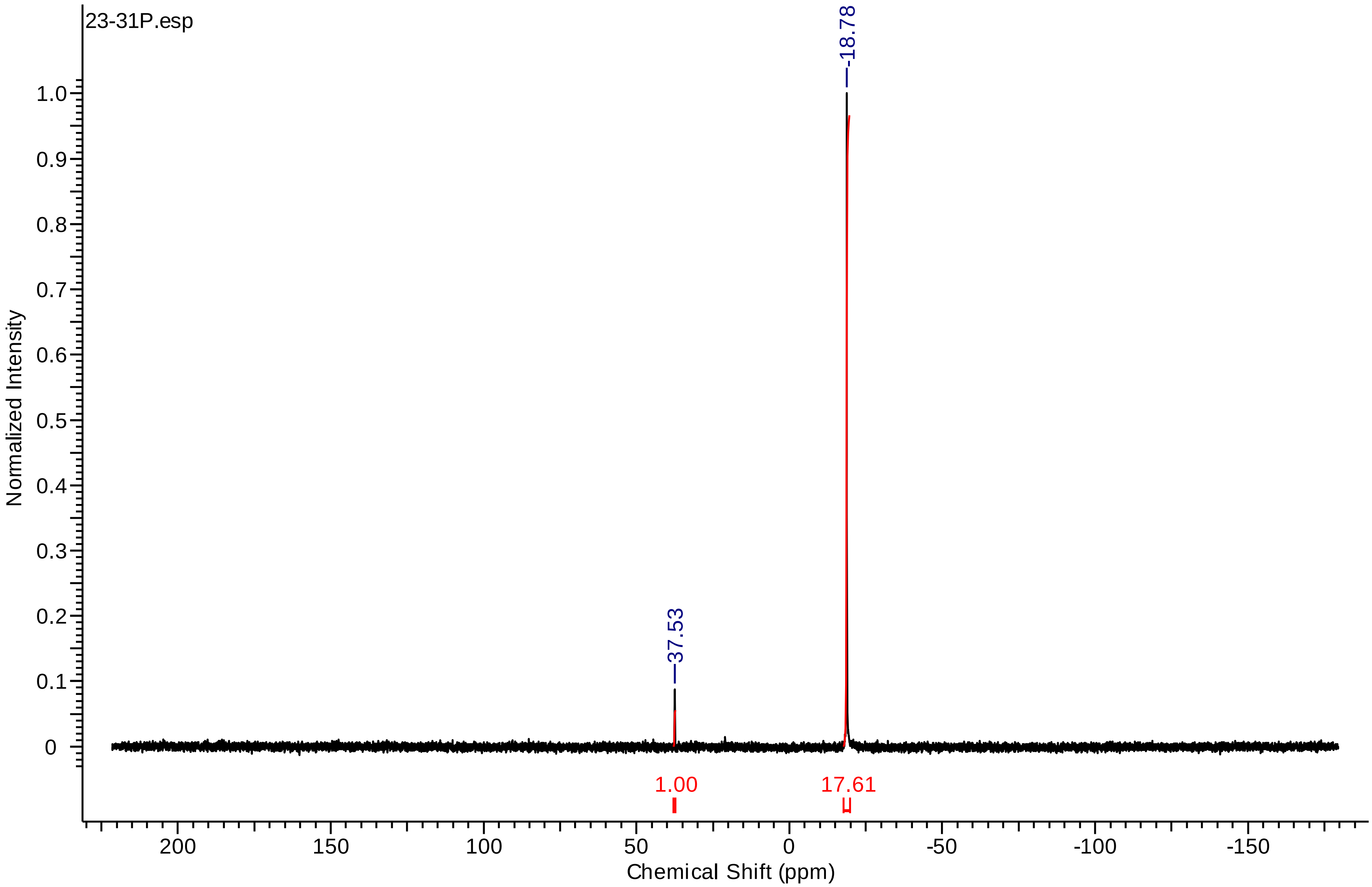

23-31P.esp
-18.78
37.53
1.00
17.61
Normalized Intensity
Chemical Shift (ppm)

Diphenyl (((1*R*,2*S*,5*R*)-δ-pinen-4-yl)methyl)phosphine oxide (26)

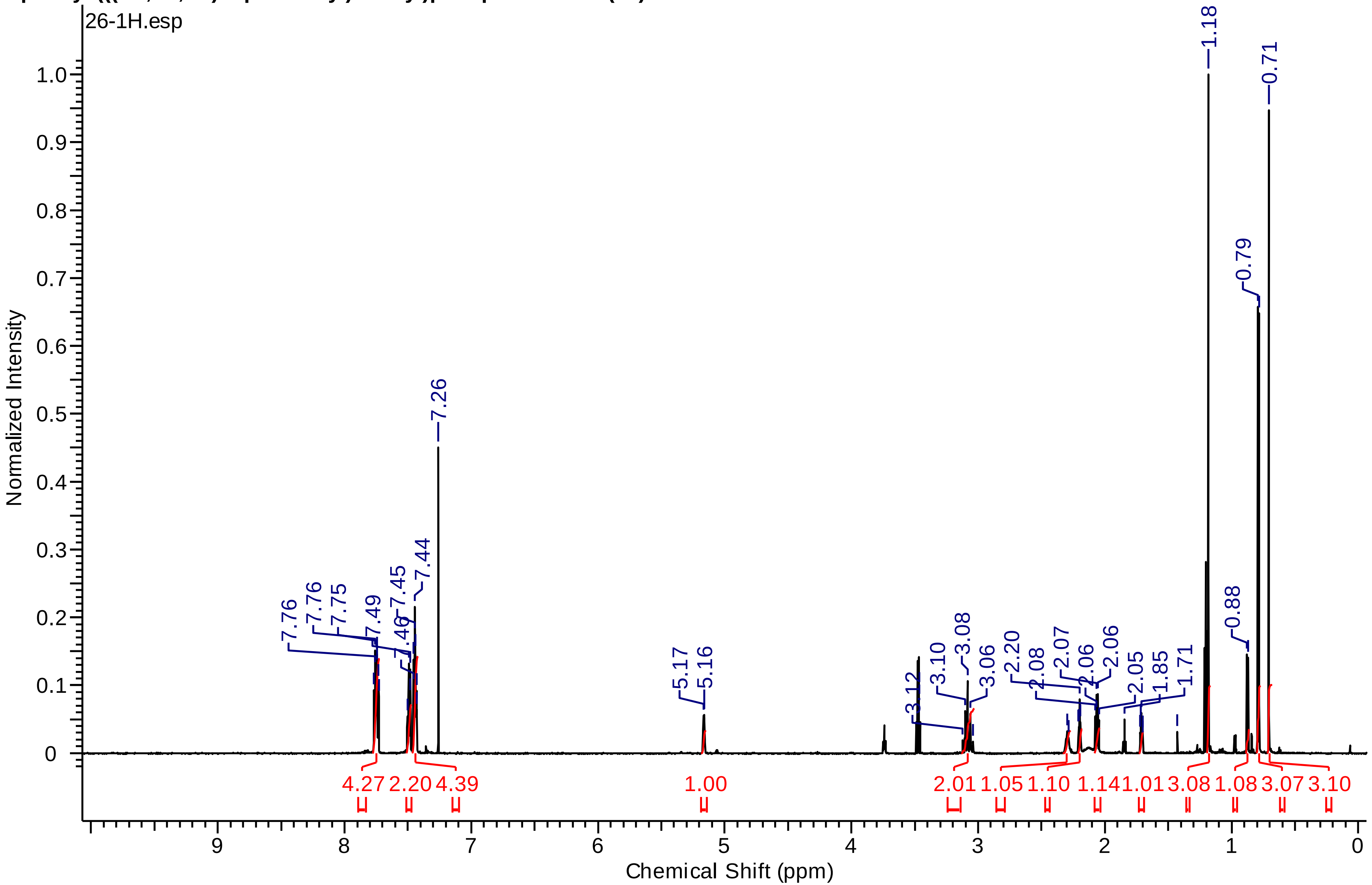

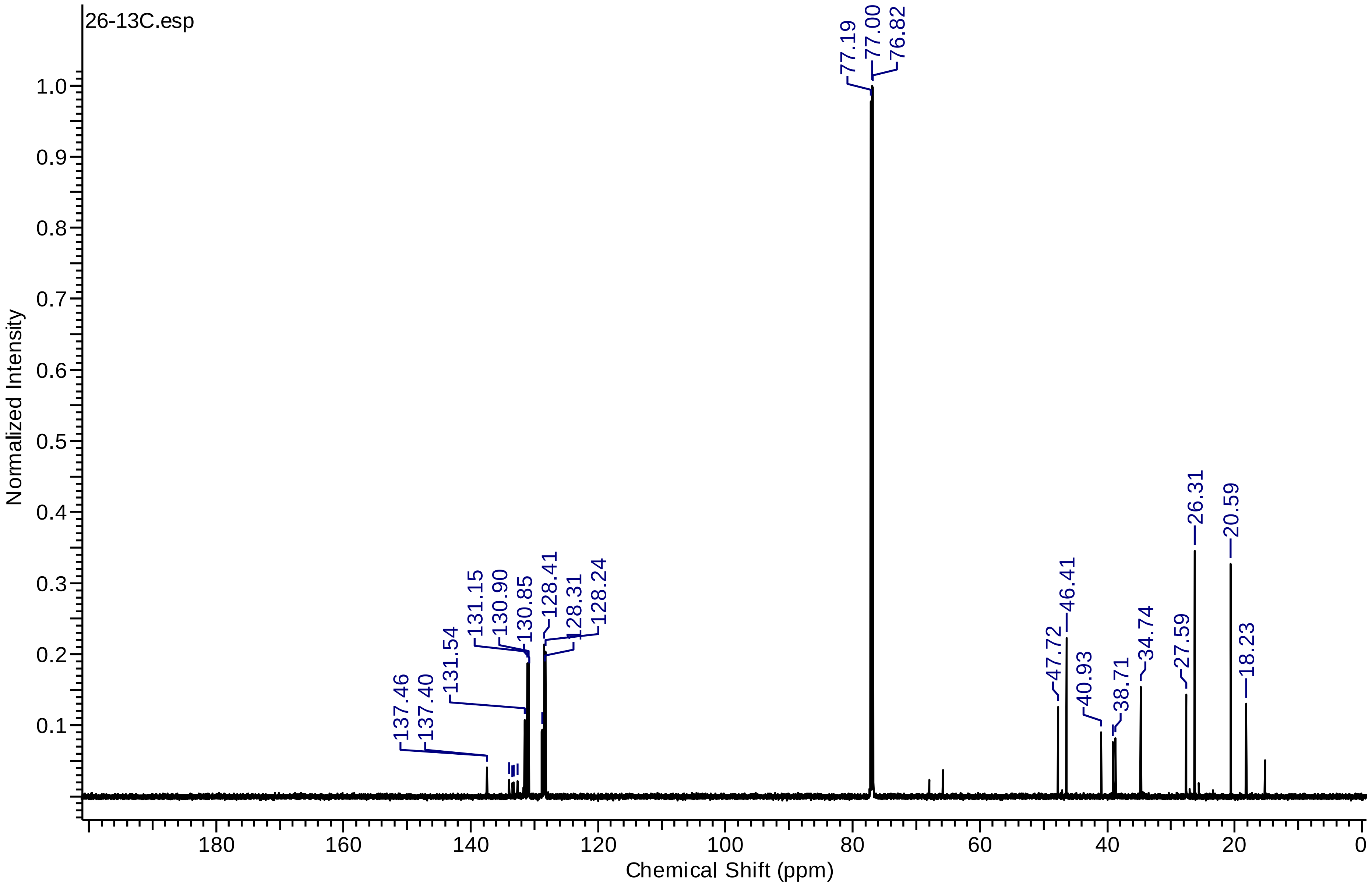
26-13C.esp
77.19
77.00
76.82
137.46
137.40
131.54
131.15
130.90
130.85
128.41
128.31
128.24
47.72
46.41
40.93
38.71
34.74
27.59
26.31
20.59
18.23
Normalized Intensity
Chemical Shift (ppm)

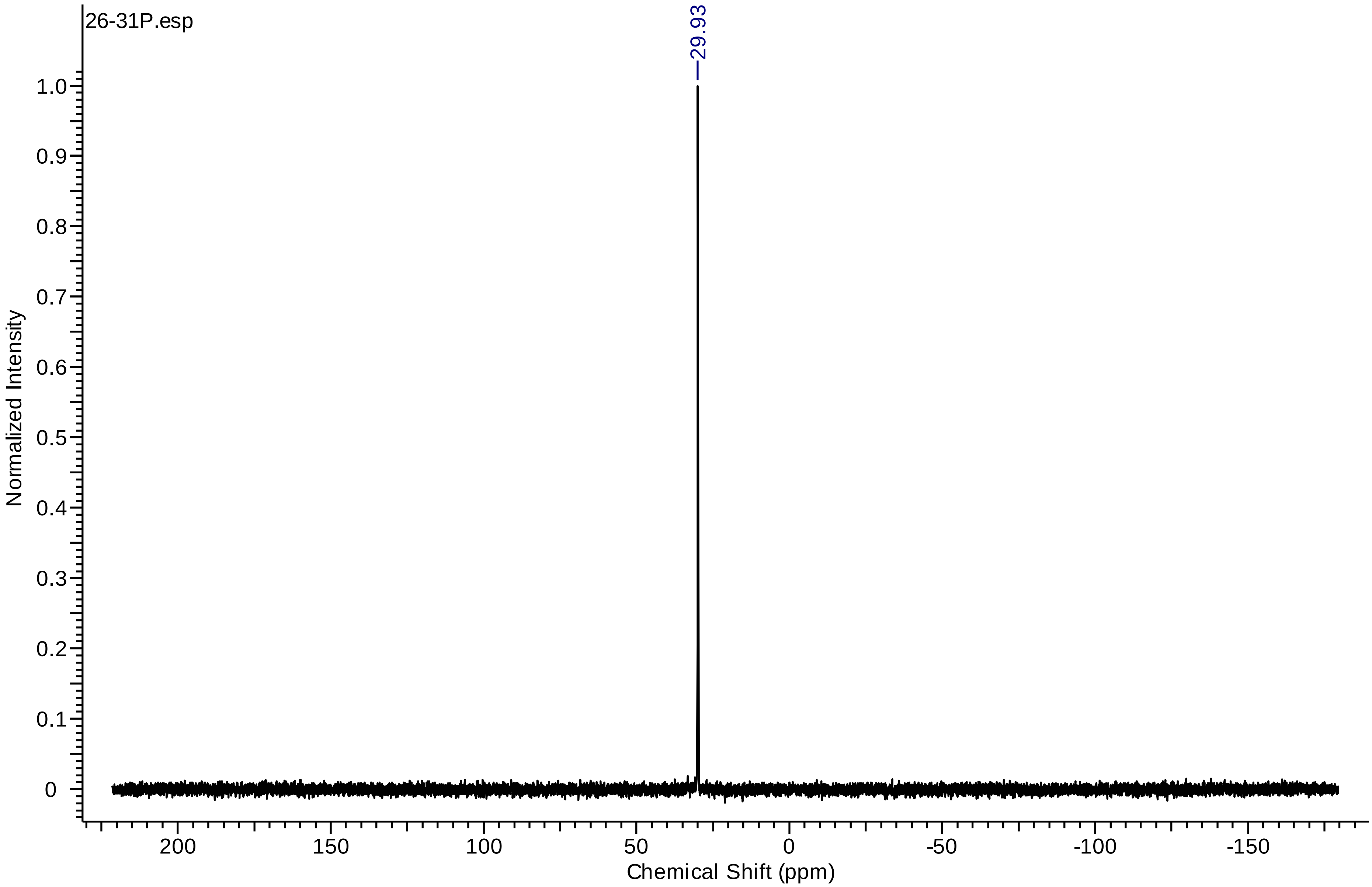
26-31P.esp
29.93
Normalized Intensity
1.0
0.9
0.8
0.7
0.6
0.5
0.4
0.3
0.2
0.1
0
200
150
100
50
0
-50
-100
-150
Chemical Shift (ppm)

(((1*R*,2*R*,3*S*,4*S*,5*S*)-3-Hydroxypinan-4-yl)methyl)diphenylphosphine oxide (27)

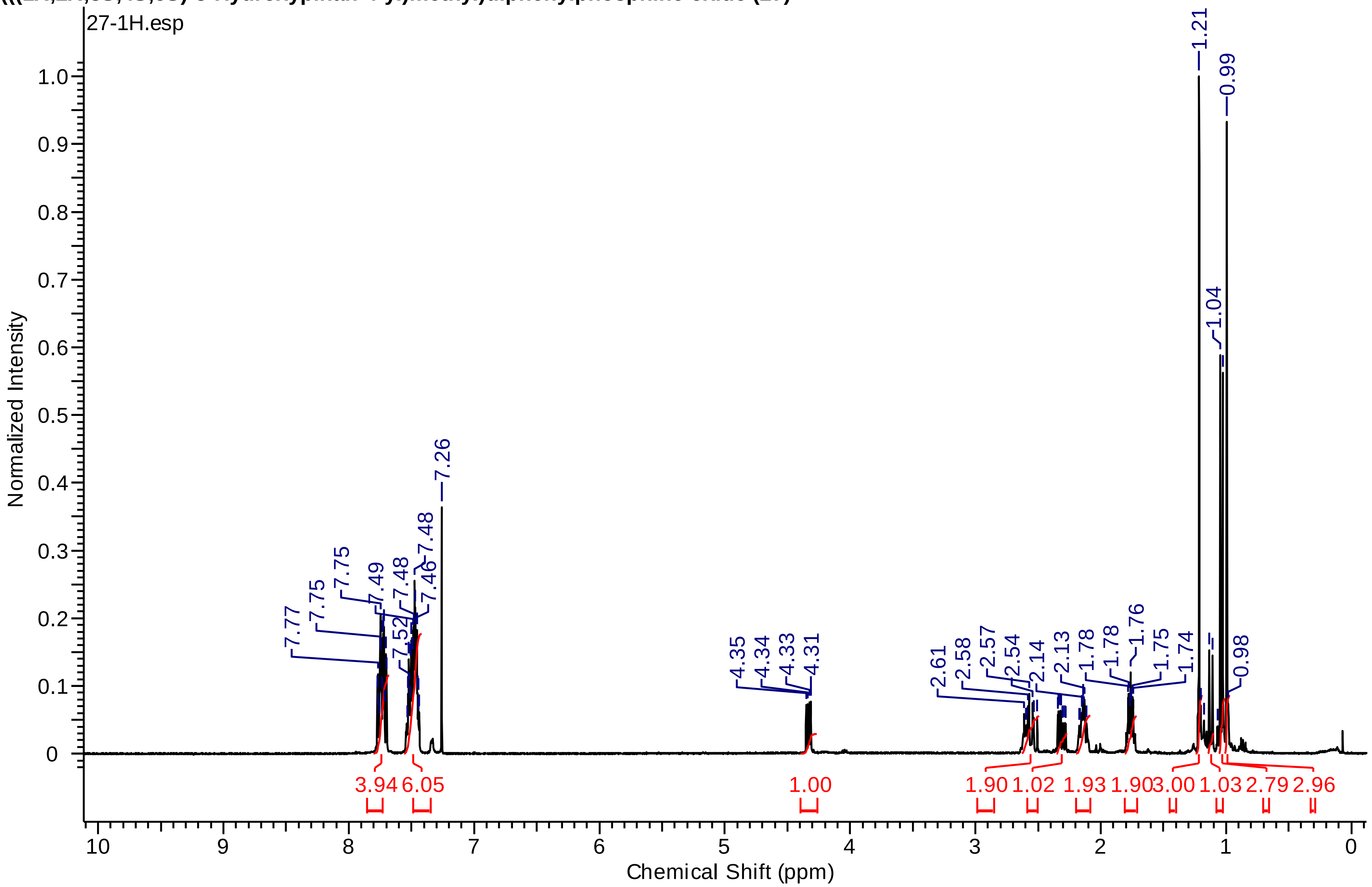

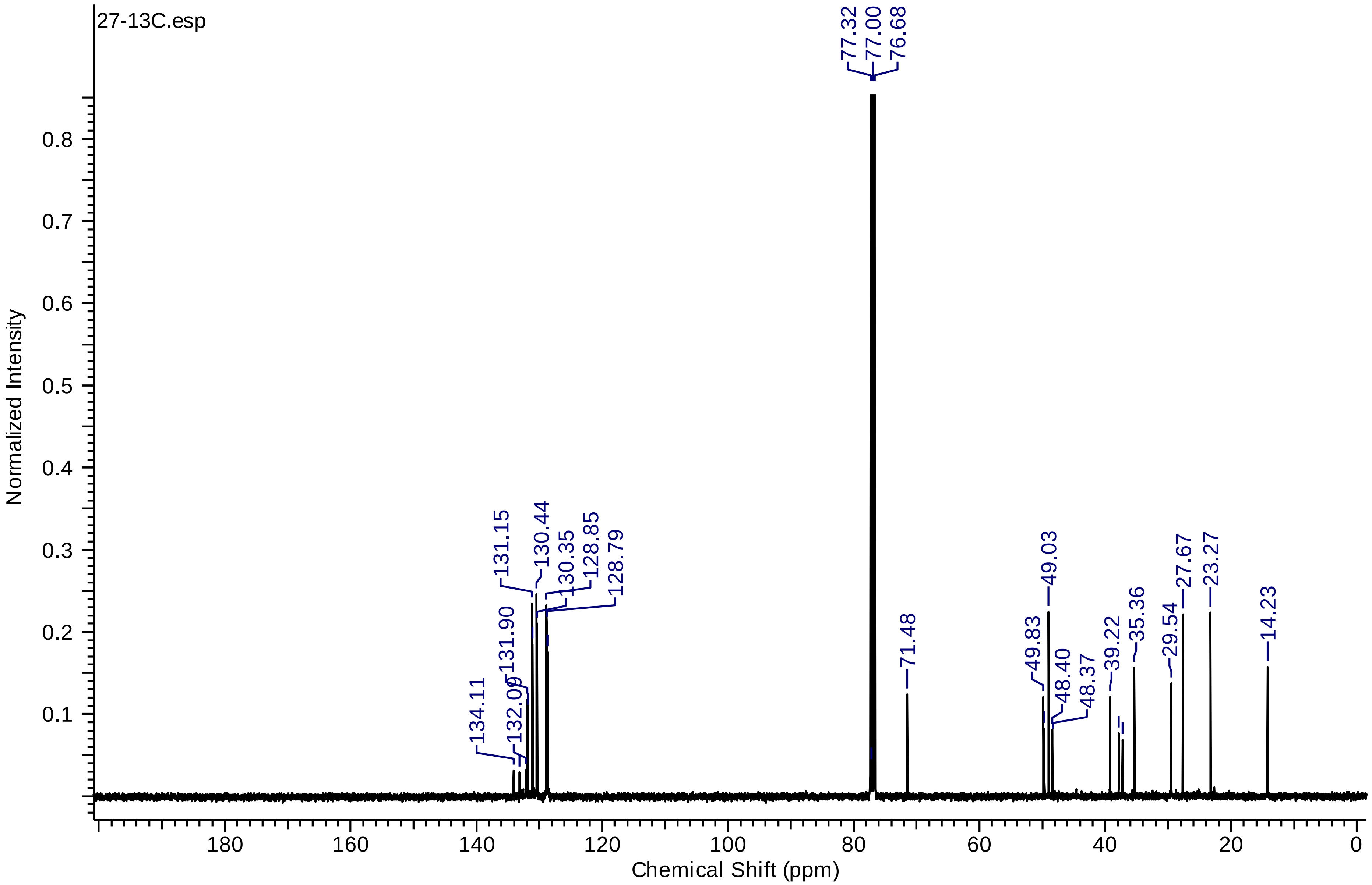
27-13C.esp
77.32
77.00
76.68
131.15
130.44
130.35
128.85
128.79
134.11
131.90
132.09
71.48
49.83
49.03
48.40
48.37
39.22
35.36
29.54
27.67
23.27
14.23
Normalized Intensity
Chemical Shift (ppm)
0.8
0.7
0.6
0.5
0.4
0.3
0.2
0.1
180
160
140
120
100
80
60
40
20
0

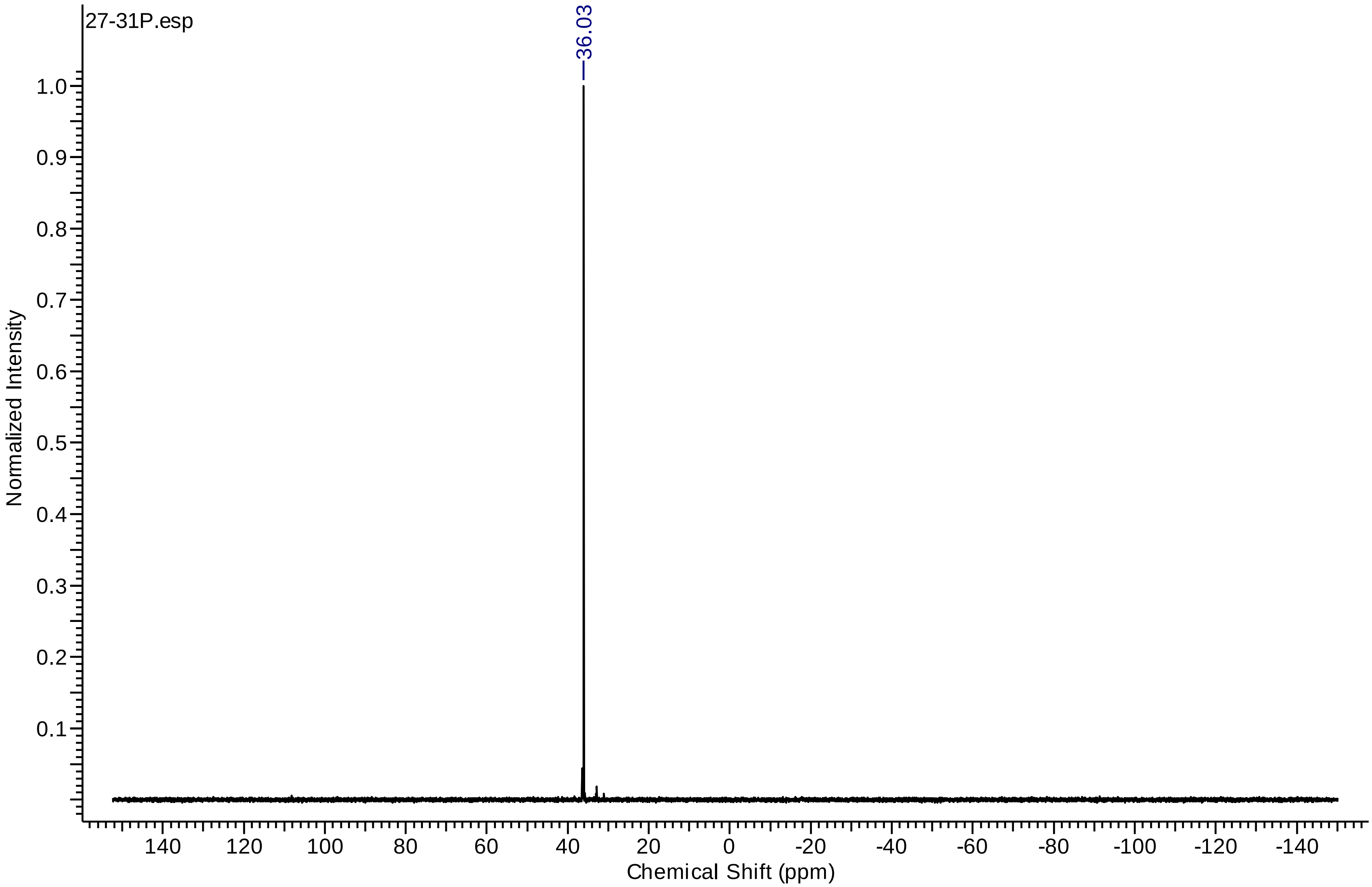
27-31P.esp
36.03
Normalized Intensity
Chemical Shift (ppm)
